# Catalyst-Free Trans-Selective Oxyiodination and Oxychlorination of Alkynes Employing N–X (Halogen) Reagents

**DOI:** 10.3390/molecules28217420

**Published:** 2023-11-03

**Authors:** Jiaqiong Sun, Yunliang Guo, Jiuli Xia, Guangfan Zheng, Qian Zhang

**Affiliations:** 1 School of Environment, Northeast Normal University, Changchun 130117, China; guoyl267@nenu.edu.cn; 2 Key Laboratory of Functional Organic Molecule Design & Synthesis of Jilin Province, Department of Chemistry, Northeast Normal University, Changchun 130024, China; xiajl699@nenu.edu.cn (J.X.); zhangq651@nenu.edu.cn (Q.Z.); 3 State Key Laboratory of Organometallic Chemistry, Shanghai Institute of Organic Chemistry, Chinese Academy of Sciences, 345 Lingling Road, Shanghai 200032, China

**Keywords:** catalyst-free, oxyiodination, oxychlorination, alkynes, N–X (halogen) reagents

## Abstract

β-halogenated enol esters and ethers are versatile building blocks in organic synthesis, which has attracted increasing attention. In this study, we report the facile *trans*-oxyiodination and oxychlorination of alkynes, leading to the direct construction of versatile halogenated enol esters and ethers. This transformation features an easy operation, optimal atomic economy, a strong functional group tolerance, broad substrate scope, and excellent trans-selectivity. Employing highly electrophilic bifunctional N–X (halogen) reagents was the key to achieving broad reaction generality. To our knowledge, this transformation represents the first oxyhalogenation system employing N–X (halogen) reagents as both oxylation and halogenation sources.

## 1. Introduction

**Scheme 1:**
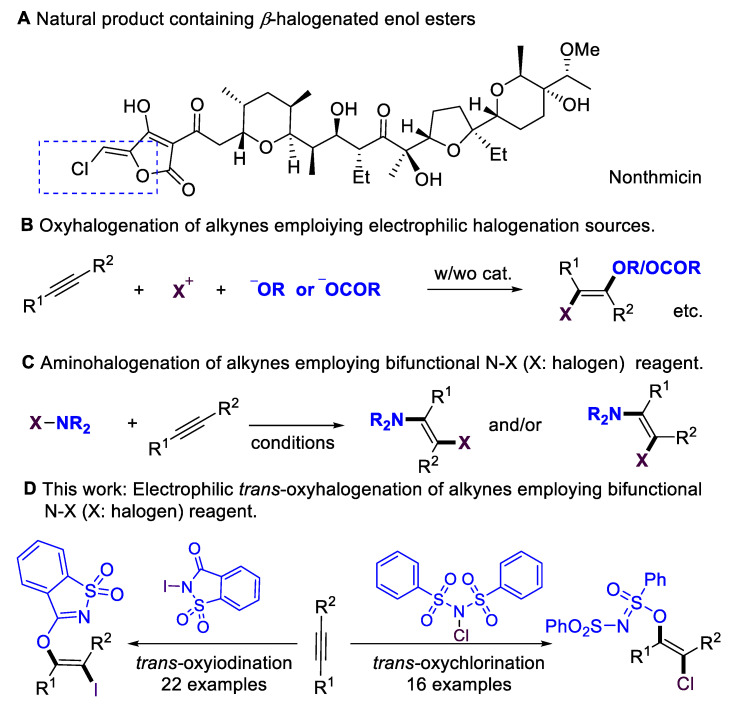
Motivation for oxyhalogenation of alkynes.

Multi-substituted enol esters and ethers have been widely applied in natural products and bioactive molecules (Scheme 1A) [1,2,3,4]; they also serve as versatile building blocks [5,6,7,8,9,10,11] in organic synthesis, medicinal chemistry, and polymer chemistry. Alkynes are fundamental and easy-to-access starting materials in organic synthesis. The stereoselective installation of O-centered functional groups in alkynes has become an attractive alternative for synthesizing enol esters/ethers. Despite the significance of hydroalkoxylation [12,13], the scope of alkynes has mainly been focused on terminal alkynes, which lead to disubstituted enol esters/ethers. Introducing halogen atoms into organic molecules is an important step in organic synthesis [14,15,16,17,18,19,20,21], as halogen groups could serve as versatile synthetic handles for further transformations [22,23,24]. The oxyhalogenation of alkynes is a straightforward route to β-halogenated enol esters and ethers by simultaneously installing O-centered groups and halogen groups into C–C triple bonds. Considerable progress has been achieved in the intramolecular oxyhalogenation of alkynes that were initiated through nucleophilic cyclization, leading to the formation of halogenated O-containing heterocycles. However, there are only a few existing examples of the intermolecular oxyhalogenation of alkynes [25,26,27,28,29,30,31,32] via the employment of electrophilic halogenation reagents and additional acid, alcohol, or phenol nucleophiles (Scheme 1B). Furthermore, intermolecular oxyhalogenation systems without additional nucleophiles have remained largely underdeveloped. This core challenge could be attributed to a lack of efficient bifunctional oxyhalogenation reagents. The development of novel bifunctional reagents, or exploring new reaction modes of existing halogenation reagents, is highly desirable.

On the other hand, N–X (halogen) reagents [14,15,16,17], such as NBS, NIS, or NFSI, are the most significant electrophilic halogenation reagents with extremely important applications in organic synthesis. They also serve as bifunctional reagents in addition to alkynes, delivering amino-halogenation products (Scheme 1C) [33,34,35,36]. In 1999, the Wille and Lüning group [34] achieved the radical amino-bromination of alkynes. In 2011, the Liu group [35] achieved the copper-catalyzed chloramination of terminal alkynes, leading to the regio- and stereoselective formation of (*E*)-β-Chloro-enesulfonamides. In 2014, the Liang and Zhang group [36] accomplished the cis-amino-halogenation of terminal alkynes with N-haloimides via alkynyl halide intermediates. *N*-iodosaccharin is a highly active electrophilic iodination source [37,38,39,40,41,42] due to the saccharin anion that is released; however, in previous reports, this reagent has never been employed as a bifunctional reagent. Inspired by reports that employ saccharin anions as O-centered nucleophilic source [43,44], here, we evaluate the possibility of employing *N*-iodosaccharins as novel bifunctional oxyiodination reagents. Based on our previous works [45,46,47,48,49,50,51,52] on the development of sustainable transformations, we now report on the intermolecular oxyiodination and oxychlorination of alkynes that employ *N*-iodosaccharin or *N*-chlorobenzenesulfonimide (NCBSI) [53,54,55] as bifunctional reagents (Scheme 1D).

## 2. Results and Discussions

We utilized 1-phenyl-1-pentyne (**1a**) and *N*-iodosaccharin (**2a**) as model substrates to test the designed transformation. The oxyiodination of alkyne proceeded when **1a** (0.2 equiv) and **2a** (1.0 equiv) in dichloromethane (DCM) were employed as the solvent, yielding the corresponding product **3a** in a 68% yield (Table 1, Entry 1). A simple adjustment in the ratio of **1a** and **2a** improved the yield of product **3a** by 86% (Entry 2). However, further increasing the amount of **2a** had no noticeable effect on the yield (Entry 3). Alternative solvents were tested (Entries 4–8), and we found that dichloroethane (DCE) and perfluorotoluene (PhCF_3_) exhibited a similar efficiency to DCM, while toluene and acetonitrile (CH_3_CN) provided substantially reduced yields. Methanol (MeOH) proved unsuitable for this transformation. Importantly, the reaction could be conducted under ambient conditions, providing oxyiodination products **3a** in 86% yields (Entry 9). Remarkably, exclusive regioselectivity and stereoselectivity were achieved in all cases.

**Scheme 2:**
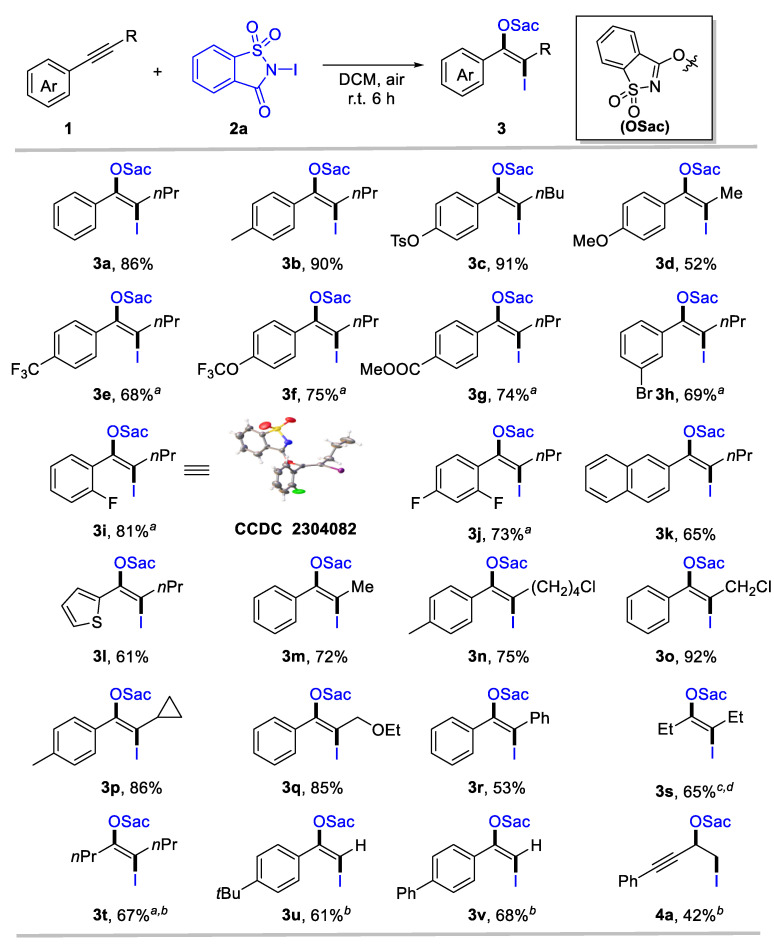
Scope for anti-oxyiodination of alkynes. Reaction conditions: **1** (0.2 mmol), *N*-iodosaccharin **2a** (1.2 equiv), DCM (2 mL), r.t., 6 h. Yield of the isolated product. *^a^* 24 h. *^b^* **2a** (0.2 mmol) and **1** (1.2 equiv) was employed, and yield was calculated based on **2a**.

Considering the easy operation, Table 1 Entry 9 was identified as the standard condition to investigate the generality and limitation of the oxyiodination system. First, the substituent effect of the aryl group for aryl-substituted internal alkynes was investigated. The top section of Scheme 2 reveals that various aromatic internal alkynes bearing electron-donating (e.g., alkyl, tosylate, and methoxy), electron-withdrawing (such as trifluoromethyl, trifluoromethoxy, and ester carbonyl), and halogen groups were well tolerated, affording the desired iodinated enol ethers **3a-3j** in moderate to high yields (52–91%). Notably, electron-donating groups in para-substituted internal aryl alkynes **1-2** and **1-3** exhibited high reactivity (**3b**, 90%; **3c**, 91%). However, strong electron-donating 1-methoxy-4-(prop-1-yn-1-yl)benzene **1-4** provides **3d** only in a moderate yield. Moreover, internal alkynes with an electron-withdrawing capacity at aromatic rings could deliver β-iodinated enol ethers with acceptable yields (**3e-3g**, 68–75%). This indicates a broad substrate scope for alkynes. Meta- and ortho-substituted aryl alkynes were tolerated, forming **3h** and **3i** in 69% and 81% yield, respectively. The structure of **3i** was confirmed through X-ray single crystal diffraction (CCDC 2304082). Even di-substituted internal aryl alkyne was a suitable substrate for this transformation, affording enol ethers **3j** in 73% yields. Naphthalene and heteroaromatics were compatible for this transformation, as identified by the formation of **3k** and **3l**. Then, the scope of aliphatic groups for aryl-substituted internal alkynes was evaluated, revealing the compatibility of alkyl (**3m**), chloride (**3n, 3o**), strained rings (**3p**), and ether (**3q**). These substrates provided the corresponding products with 72–92% yields. In all cases, specific regio- and stereo-specificity were observed. In some cases, such as diaryl (**3r**) or dialkyl-substituted alkynes (**3s**, **3t**), a reduced reaction efficiency was noted, despite their tolerance. In addition, terminal alkynes were used to explore the scope of this system further, delivering the desired enol ethers **3u** and **3v** in 61% and 68% yields, respectively. For 1,3-enynes, oxyiodination was preferred to occur at olefin units, as identified by the formation of **4a**. However, *N*-chlorosaccharin and *N*-bromosaccharin failed to deliver the desired oxyhalogenation products under standard conditions, likely owing to their considerably low electrophilic reactivity.

**Scheme 3:**
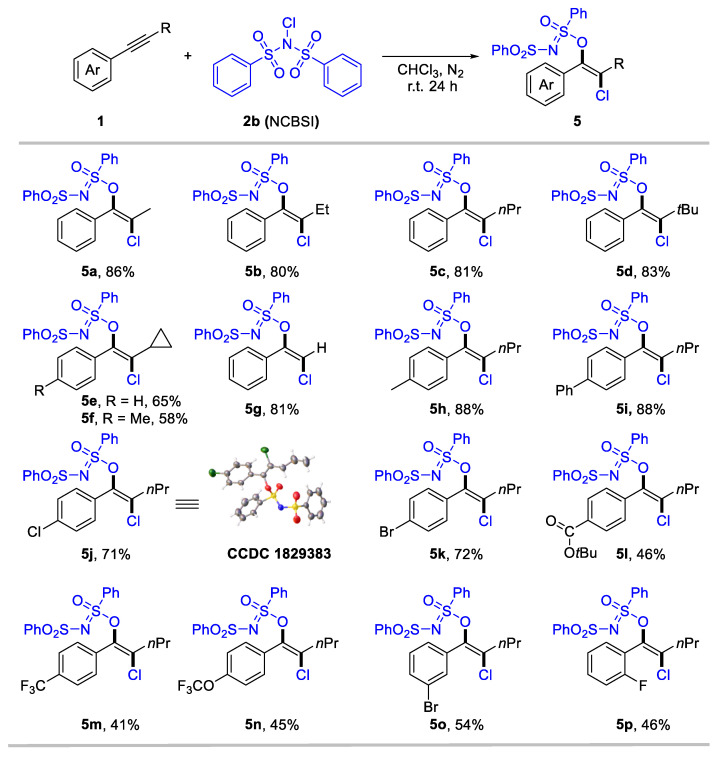
Subtract scope for *anti*-oxychlorination of alkynes. Reaction conditions: **1** (0.2 mmol), NCBSI **2b** (2.0 equiv), CHCl_3_ (2 mL), r.t. for 24 h under nitrogen atmosphere. Yield of isolated products.

We consider that the highly electron-deficient bisphenylsulfonimide may improve the electrophilic activity of N–X (X: halogen) reagents, thereby promoting the formation of halogenated products. Gratefully, when employing *N*-chlorobenzenesulfonimide (NCBSI) as the chlorination reagent, we obtained trans-oxychlorination products in chloroform under a N_2_ atmosphere (details for optimization conditions see Supplementary Materials, Table S1). The scope of the oxychlorination of alkynes was subsequently examined, and results are summarized in Scheme 3. The desired enol sulfinimidates (**5a-5p**) were obtained in moderate-to-high yields with specific anti-selectivity. Internal aryl alkynes bearing different aliphatic substituents, such as alkyl (**5a-5f**), bulky tertiary butyl (**5d**), and cyclopropyl (**5e, 5f**), were tolerated, delivering anti-oxychlorination products in 58–86% yields. Terminal aryl alkyne was also suitable for this conversion, with **5g** achieving an 81% yield. We next investigate the substituent effect of the aryl rings in internal alkynes for oxychlorination system. Aryl alkynes bearing electron-donating (**5h**), phenyl (**5i**), and halogen (**5j-5k**) groups at the para-position, performed effectively (71–88%). Notably, the structure of **5j** was confirmed through X-ray single crystal diffraction (CCDC 1829383). Meanwhile, the electron-withdrawing group, including ester carbonyl (**5l**), trifluoromethyl (**5m**), and trifluoromethoxy (**5n**), that substituted aryl acetylenes generated a slightly low yield (41–46%). Moreover, meta- and ortho-substituted aryl alkynes were applicable for the reaction, delivering **5o** and **5p** in 54% and 46% yield, respectively.

## 3. Mechanistic Investigation

**Scheme 4:**
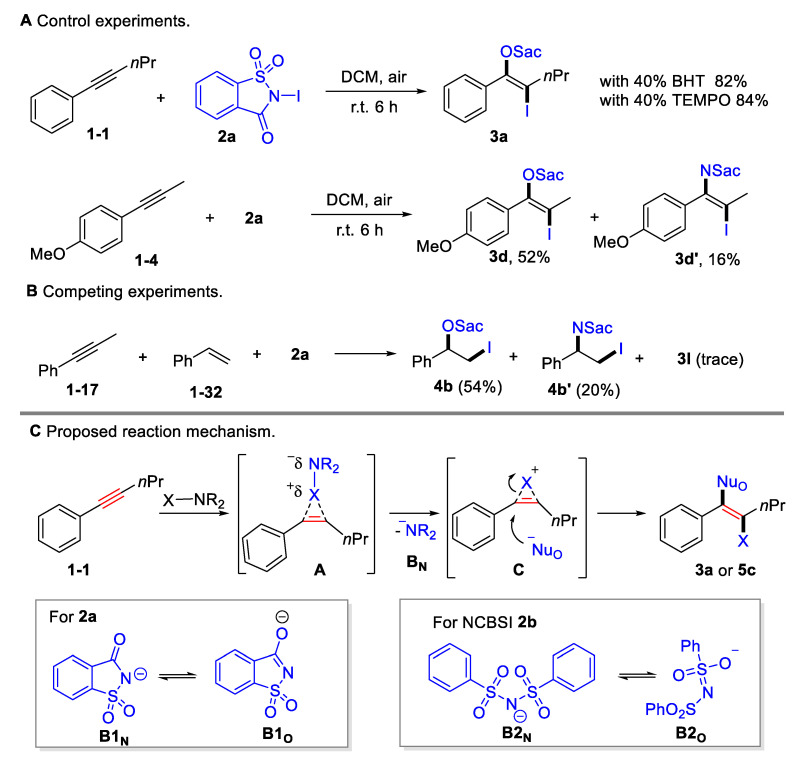
Mechanistic investigation and proposed reaction pathway.

Several preliminary mechanistic studies were conducted in order to shed insight onto this transformation (Scheme 4A upper). The addition of radical scavengers, such as butylated hydroxytoluene (BHT; 40 mol%) or 2,2,6,6-tetramethyl-1-piperidinyloxy (TEMPO; 40 mol%), showed no considerable impact on the yields, indicating that the oxyiodination may not proceed via a radical pathway. Competitive amino iodination products were obtained for 1-methoxy-4-(prop-1-yn-1-yl)benzene **1-4** or styrene **1-32** (Scheme 4A lower, Scheme 4B), indicating that there was equilibration between *N*-centered and *O*-centered nucleophile. Moreover, it was found that styrene **1-32** exhibited a higher reactivity than alkynes **1-17**, which is consistent with their electrophilic reactivity (Scheme 4B). A plausible mechanism was proposed based on the control experiment and on previous reports [45] (Scheme 4C). The process begins with the selective coordination of alkyne triple bonds and halogenating reagents, generating a cyclic halonium onium intermediate **A** by releasing a saccharin anion B1_N_ or benzenesulfonamide anion **B2_N_**. The released **B1_N_** or **B2_N_** could resonate to the *O*-centered nucleophiles **B1_O_** or **B2_O_**. Owing to its steric hindrance, halonium onium intermediate preferred to be opened by *O*-centered nucleophiles, resulting in anti-selective oxyhalogenation.

## 4. Materials and Methods

### 4.1. Materials and Instruments

All chemicals were obtained from commercial sources and were used as they were received, unless otherwise noted. All reactions were carried out using a test tube or a pressure tube. The reactions were monitored with the aid of thin-layer chromatography (TLC) on 0.25 mm precoated silica gel plates. Visualization was carried out with a UV light and a aqueous potassium permanganate stain. Melting points were measured on Büchi B-540 apparatus. NMR spectra were recorded on a 500 or 600 MHz NMR spectrometer in the solvent indicated. The chemical shift is given in dimensionless *δ* values and is frequency-referenced, relative to TMS in ^1^H and ^13^C NMR spectroscopy. HRMS data were obtained on a Bruck microtof. Column chromatography was performed on silica gel (200–300 mesh) using ethyl acetate/hexanes.

### 4.2. The General Procedure for the Synthesis of ***3***

The reaction tube equipped with a magnetic stir bar was charged with **2a** (0.24 mmol, 74.1 mg), DCM (2 mL), and **1** (0.2 mmol). The test tube was then sealed off with a screw cap, and the reaction mixture was stirred at room temperature for 6.0 or 24 h. After the reaction was completed, as indicated by TLC analysis, the mixture was extracted using DCM (2 × 10 mL). The combined organic phases were dried over anhydrous Na_2_SO_4_, and the solvent was evaporated under a vacuum. The residue was purified via column chromatography (petroleum ether/ethyl acetate 10:1 (*v*/*v*)) to provide the corresponding product **3**.

(*E*)-3-((2-iodo-1-phenylpent-1-en-1-yl)oxy)benzo[*d*]isothiazole 1,1-dioxide **3a**

**1-1** (0.2 mmol, 28.8 mg) and **2a** (0.24 mmol, 74.1 mg) were employed. **3a** (PE/EtOAc = 5:1, Rf = 0.3) was purified by column chromatography on silica gel (PE/EtOAc = 10:1), yellow solid (83.7 mg, 86%), crystallization in CDCl_3_, mp. 140–142 °C. NMR spectroscopy: ^1^H NMR (600 MHz, CDCl_3_) δ 7.86 (d, *J* = 7.2 Hz, 1H), 7.82 (d, *J* = 7.8 Hz, 1H), 7.77 (td, *J* = 7.2, 1.2 Hz, 1H), 7.73 (td, *J* = 7.8, 1.2 Hz, 1H), 7.60–7.58 (m, 2H), 7.39–7.35 (m, 3H), 2.57 (t, *J* = 7.2 Hz, 2H), 1.72–1.65 (m, 2H), 0.98 (t, *J* = 7.2 Hz, 3H). ^13^C NMR (150 MHz, CDCl_3_) δ 167.3, 147.0, 143.9, 135.3, 134.3, 133.6, 130.3, 129.8, 128.2, 126.2, 123.3, 122.1, 98.7, 39.8, 22.3, 13.1. Mass spectrometry: HRMS (ESI-TOF) (*m*/*z*): Calcd for C_18_H_16_INNaO_3_S^+^ ([M + Na]^+^), 475.9788, found, 475.9790.

(*E*)-3-((2-iodo-1-(p-tolyl)pent-1-en-1-yl)oxy)benzo[*d*]isothiazole 1,1-dioxide **3b**

**1-2** (0.2 mmol, 31.6 mg) and **2a** (0.24 mmol, 74.1 mg) were employed. **3b** (PE/EtOAc = 5:1, Rf = 0.3) was purified by column chromatography on silica gel (PE/EtOAc = 10:1), white solid (84.5 mg, 90%), crystallization in CDCl_3_, mp. 106–108 °C. NMR spectroscopy: ^1^H NMR (600 MHz, CDCl_3_) δ 7.85 (d, *J* = 7.8 Hz, 1H), 7.81 (d, *J* = 7.8 Hz, 1H), 7.76 (td, *J* = 7.2, 1.2 Hz, 1H), 7.72 (td, *J* = 7.8, 1.2 Hz, 1H), 7.48 (d, *J* = 7.8 Hz, 2H), 7.17 (d, *J* = 7.8 Hz, 2H), 2.57–2.55 (m, 2H), 2.35 (s, 3H), 1.70–1.64 (m, 2H), 0.97 (t, *J* = 7.2 Hz, 3H). ^13^C NMR {^1^H} (150 MHz, CDCl_3_) δ 167.3, 147.1, 143.9, 139.9, 134.3, 133.5, 132.4, 130.2, 128.9, 126.2, 123.3, 122.1, 98.3, 39.9, 22.3, 21.4, 13.1. Mass spectrometry: HRMS (ESI-TOF) (*m*/*z*): Calcd for C_19_H_18_INNaO_3_S^+^ ([M + Na]^+^), 489.9944, found, 489.9950.

(*E*)-4-(1-((1,1-dioxidobenzo[*d*]isothiazol-3-yl)oxy)-2-iodohex-1-en-1-yl)phenyl 4-methylbenzenesulfonate **3c**

**1-3** (0.2 mmol, 65.6 mg) and **2a** (0.24 mmol, 74.1 mg) were employed. **3c** (PE/EtOAc = 5:1, Rf = 0.3) was purified by column chromatography on silica gel (PE/EtOAc = 10:1), white solid (116.3 mg, 91%), crystallization in CDCl_3_, mp. 109–111 °C. NMR spectroscopy: ^1^H NMR (600 MHz, CDCl_3_) δ 7.86 (d, *J* = 7.2 Hz, 1H), 7.80 (dt, *J* = 7.2, 4.2 Hz, 2H), 7.75 (td, *J* = 7.2, 0.6 Hz, 1H), 7.64 (d, *J* = 8.4 Hz, 2H), 7.55–7.50 (m, 2H), 7.29 (d, *J* = 7.8 Hz, 2H), 7.00–6.93 (m, 2H), 2.63–2.55 (m, 2H), 2.42 (s, 3H), 1.63–1.58 (m, 2H), 1.41–1.31 (m, 2H), 0.90 (t, *J* = 7.2 Hz, 3H). ^13^C NMR {^1^H} (150 MHz, CDCl_3_) δ 167.2, 150.2, 145.6, 145.4, 143.8, 134.5, 134.1, 133.7, 131.9, 129.8, 128.5, 125.8, 123.3, 122.3, 122.1, 99.7, 37.7, 30.9, 21.7, 21.6, 13.8. Mass spectrometry: HRMS (ESI-TOF) (*m*/*z*): Calcd for C_26_H_24_INNaO_2_S_2_^+^, ([M + Na]^+^), 659.9982, found, 659.9990.

(*E*)-3-((2-iodo-1-(4-methoxyphenyl)prop-1-en-1-yl)oxy)benzo[*d*]isothiazole 1,1-dioxide **3d**

**1-4** (0.2 mmol, 29.2 mg) and **2a** (0.24 mmol, 74.1 mg) were employed. **3d** (PE/EtOAc = 5:1, Rf = 0.28) was purified by column chromatography on silica gel (PE/EtOAc = 10:1), colorless oil (47.2 mg, 52%). NMR spectroscopy: ^1^H NMR (500 MHz, CDCl_3_) δ 7.87 (d, *J* = 7.3 Hz, 1H), 7.83 (d, *J* = 7.4 Hz, 1H), 7.80–7.75 (m, 1H), 7.73 (td, *J* = 7.5, 1.1 Hz, 1H), 7.53 (d, *J* = 8.8 Hz, 2H), 6.89 (d, *J* = 8.8 Hz, 2H), 3.81 (s, 3H), 2.57 (s, 3H). ^13^C NMR {^1^H} (150 MHz, CDCl_3_) δ 166.8 (C=N), 160.5, 147.2, 143.4, 134.3, 133.6, 131.7, 127.3, 126.2, 123.4, 122.1, 121.7, 113.6, 86.2 (C-I), 53.0, 26.4. Mass spectrometry: HRMS (ESI-TOF) (*m*/*z*): Calcd for C_17_H_14_INNaO_4_S^+^ ([M + Na]^+^), 477.9580, found 477.9594.

(*E*)-2-(2-iodo-1-(4-methoxyphenyl)prop-1-en-1-yl)benzo[d]isothiazol-3(2H)-one 1,1-dioxide **3d′**

**1-4** (0.2 mmol, 29.2 mg) and **2a** (0.24 mmol, 74.1 mg) were employed. **3d′** (PE/EtOAc = 5:1, Rf = 0.32) was purified by column chromatography on silica gel (PE/EtOAc = 10:1), colorless oil (14.6 mg, 16%). NMR spectroscopy: ^1^H NMR (500 MHz, Chloroform-*d*) δ 8.08 (d, *J* = 6.7 Hz, 1H), 7.96–7.79 (m, 3H), 7.48 (d, *J* = 8.8 Hz, 2H), 6.88 (d, *J* = 8.8 Hz, 2H), 3.80 (s, 3H), 2.71 (s, 3H). ^13^C NMR {^1^H} (150 MHz, CDCl_3_) δ 159.9, 157.2 (C=O), 138.1, 135.0, 134.4, 131.6, 131.3, 126.7, 125.6, 121.1, 113.5, 109.7 (C-I), 54.4, 30.5. Mass spectrometry: HRMS (ESI-TOF) (*m*/*z*): Calcd for C_17_H_14_INNaO_4_S^+^ ([M + Na]^+^), 477.9580, found 477.9580.

(*E*)-3-((2-iodo-1-(4-(trifluoromethyl)phenyl)pent-1-en-1-yl)oxy)benzo[*d*]isothiazole **3e**

**1-5** (0.2 mmol, 42.4 mg) and **2a** (0.24 mmol, 74.1 mg) were employed. **3e** (PE/EtOAc = 5:1, Rf = 0.3) was purified by column chromatography on silica gel (PE/EtOAc = 10:1), yellow oil (70.8 mg, 68%). NMR spectroscopy: ^1^H NMR (500 MHz, CDCl_3_) δ 7.87 (d, *J* = 7.5 Hz, 1H), 7.82 (d, *J* = 7.5 Hz, 1H), 7.79 (t, *J* = 7.5 Hz, 1H), 7.76–7.72 (m, 3H), 7.64 (d, *J* = 8.0 Hz, 2H), 2.59 (t, *J* = 7.5 Hz, 2H), 1.73–1.63 (m, 2H), 0.98 (t, *J* = 7.5 Hz, 3H). ^13^C NMR {^1^H} (150 MHz, CDCl_3_) δ 167.3, 145.4, 143.8, 138.8, 134.6, 133.7, 131.5 (q, *J* = 33.0 Hz), 130.9, 125.8, 125.3 (q, *J* = 4.5 Hz), 123.7 (q, *J* = 271.5 Hz), 123.2, 122.2, 100.1, 39.8, 22.3, 13.1. ^19^F NMR (565 MHz, CDCl_3_) δ −62.92. Mass spectrometry: HRMS (ESI-TOF) (*m*/*z*): Calcd for C_19_H_15_F_3_INNaO_3_S^+^, ([M + Na]^+^), 543.9662, found, 543.9661.

(*E*)-3-((2-iodo-1-(4-(trifluoromethoxy)phenyl)pent-1-en-1-yl)oxy)benzo[*d*]isothiazole 1,1-dioxide **3f**

**1-6** (0.2 mmol, 25.6 mg) and **2a** (0.24 mmol, 74.1 mg) were employed. **3f** (PE/EtOAc = 5:1, Rf = 0.3) was purified by column chromatography on silica gel (PE/EtOAc = 10:1), yellow oil (80.9 mg, 75%). NMR spectroscopy: ^1^H NMR (600 MHz, CDCl_3_) δ 7.87 (d, *J* = 7.8 Hz, 1H), 7.82 (d, *J* = 7.2 Hz, 1H), 7.79 (t, *J* = 7.8 Hz, 1H), 7.74 (t, *J* = 7.8 Hz, 1H), 7.64 (d, *J* = 8.4 Hz, 2H), 7.22 (d, *J* = 8.4 Hz, 2H), 2.58 (t, *J* = 7.2 Hz, 2H), 1.71–1.65 (m, 2H), 0.97 (t, *J* = 7.2 Hz, 3H). ^13^C NMR {^1^H} (150MHz, CDCl_3_) δ 167.3, 149.9, 145.6, 143.8, 134.5, 133.8, 133.7, 132.2, 125.9, 123.3, 122.2, 120.4, 120.3 (q, *J* = 256.5 Hz) 99.8, 39.8, 22.3, 13.1. ^19^F NMR (565 MHz, CDCl_3_) δ −57.64. Mass spectrometry: HRMS (ESI-TOF) (*m*/*z*): Calcd for C_19_H_25_F_3_INNaO_4_S^+^, ([M + Na]^+^), 559.9611, found, 559.9624.

methyl (*E*)-4-(1-((1,1-dioxidobenzo[*d*]isothiazol-3-yl)oxy)-2-iodopent-1-en-1-yl)benzoate **3g**

**1-7** (0.2 mmol, 40.4 mg) and **2a** (0.24 mmol, 74.1 mg) were employed. **3g** (PE/EtOAc = 5:1, Rf = 0.3) was purified by column chromatography on silica gel (PE/EtOAc = 10:1), yellow oil (75.6 mg, 74%). NMR spectroscopy: ^1^H NMR (600 MHz, CDCl_3_) δ 8.05 (d, *J* = 8.4 Hz, 2H), 7.86 (d, *J* = 7.2 Hz, 1H), 7.84 (d, *J* = 7.2 Hz, 1H), 7.79 (t, *J* = 7.2 Hz, 1H), 7.75 (t, *J* = 7.2 Hz, 1H), 7.69 (d, *J* = 8.4 Hz, 2H), 3.91 (s, 3H), 2.60 (t, *J* = 7.2 Hz, 2H), 1.73–1.64 (m, 2H), 0.98 (t, *J* = 7.2 Hz, 3H). ^13^C NMR {^1^H} (150 MHz, CDCl_3_) δ 167.3, 166.3, 145.8, 143.7, 139.5, 134.5, 133.7, 131.0, 130.4, 129.5, 125.8, 123.2, 122.2, 99.7, 52.2, 39.8, 22.2, 13.1. Mass spectrometry: HRMS (ESI-TOF) (*m*/*z*): Calcd for C_20_H_18_INNaO_5_S^+^ ([M + Na]^+^), 533.9843, found, 533.9848.

(*E*)-3-((1-(3-bromophenyl)-2-iodopent-1-en-1-yl)oxy)benzo[*d*]isothiazole 1,1-dioxide **3h**

**1-12** (0.2 mmol, 44.4 mg) and **2a** (0.24 mmol, 74.1 mg) were employed. **3h** (PE/EtOAc = 5:1, Rf = 0.3) was purified by column chromatography on silica gel (PE/EtOAc = 10:1), yellow solid (73.2 mg, 69%), crystallization in CDCl_3_, mp. 111–113 °C. NMR spectroscopy: ^1^H NMR (600 MHz, CDCl_3_) δ 7.86 (d, *J* = 7.8Hz, 1H), 7.82 (d, *J* = 7.8 Hz, 1H), 7.79 (td, *J* = 7.8, 1.2 Hz, 1H), 7.74 (td, *J* = 7.2, 1.2 Hz, 1H), 7.70 (t, *J* = 1.8 Hz, 1H), 7.59–7.57 (m, 1H), 7.50–7.48 (m, 1H), 7.26–7.25 (m, 1H), 2.58–2.56 (m, 2H), 1.70–1.64 (m, 2H), 0.97 (q, *J* = 7.8 Hz, 3H).^13^C NMR {^1^H} (150 MHz, CDCl_3_) δ 167.3, 145.4, 143.8, 137.2, 134.5, 133.6, 132.8, 132.7, 129.8, 129.5, 125.9, 123.3, 122.2, 122.0, 99.9, 39.8, 22.3, 13.1. Mass spectrometry: HRMS (ESI-TOF) (*m*/*z*): Calcd for C_18_H_15_BrINNaO_3_S^+^ ([M + Na]^+^), 553.8893, found, 553.8899.

(*E*)-3-((1-(2-fluorophenyl)-2-iodopent-1-en-1-yl)oxy)benzo[*d*]isothiazole **3i**

**1-13** (0.2 mmol, 32.4 mg) and **2a** (0.24 mmol, 74.1 mg) were employed. **3i** (PE/EtOAc = 5:1, Rf = 0.3) was purified by column chromatography on silica gel (PE/EtOAc = 10:1), white solid (76.1 mg, 81%), crystallization in CDCl_3_, mp. 106–108 °C. NMR spectroscopy: ^1^H NMR (600 MHz, CDCl_3_) δ 7.86 (d, *J* = 7.8 Hz, 1H), 7.82 (d, *J* = 7.8 Hz, 1H), 7.77 (td, *J* = 7.2, 0.6 Hz, 1H), 7.72 (td, *J* = 7.8, 0.6 Hz, 1H), 7.67 (td, *J* = 7.2, 1.8 Hz, 1H), 7.40–7.36 (m, 1H), 7.19 (td, *J* = 7.8, 0.6 Hz, 1H), 7.09 (t, *J* = 9.6 Hz, 1H), 2.60 (t, *J* = 7.2 Hz, 2H), 1.72–1.66 (m, 2H), 0.99 (t, *J* = 7.2 Hz, 3H). ^13^C NMR {^1^H} (150 MHz, CDCl_3_) δ 167.2, 159.9 (d, *J* = 250.0 Hz), 159.1, 143.8, 142.4, 134.4, 133.6 (d, *J* = 21.0 Hz), 132.0 (d, *J* = 8.1 Hz), 126.0, 124.0 (d, *J* = 3.5 Hz), 123.4, 123.3, 122.1, 115.8 (d, *J* = 21.0 Hz), 102.5, 39.5, 22.2, 12.9. Mass spectrometry: HRMS (ESI-TOF) (*m*/*z*): Calcd for C_18_H_15_FINNaO_3_S^+^ ([M + Na]^+^), 493.9694, found, 493.9677.

(*E*)-3-((1-(2,4-difluorophenyl)-2-iodopent-1-en-1-yl)oxy)benzo[*d*]isothiazole 1,1-dioxide **3j**

**1-14** (0.2 mmol, 36.0 mg) and **2a** (0.24 mmol, 74.1 mg) were employed. **3j** (PE/EtOAc = 5:1, Rf = 0.3) was purified by column chromatography on silica gel (PE/EtOAc = 10:1), white solid (71.6 mg, 73%), crystallization in CDCl_3_, mp. 109–111 °C. NMR spectroscopy: ^1^H NMR (600 MHz, CDCl_3_) δ 7.86 (d, *J* = 7.8 Hz, 1H), 7.82 (d, *J* = 7.8 Hz, 1H), 7.79 (td, *J* = 7.2, 1.2 Hz, 1H), 7.74 (td, *J* = 7.2, 1.2 Hz, 1H), 7.67 (td, *J* = 8.4, 6.6 Hz, 1H), 6.93 (td, *J* = 8.0, 2.4 Hz, 1H), 6.84 (td, *J* = 8.4, 2.4 Hz, 1H), 2.60 (t, *J* = 7.2 Hz, 2H), 1.71–1.63 (m, 2H), 0.99 (t, *J* = 7.2 Hz, 3H). ^13^C NMR {^1^H} (150 MHz, CDCl_3_) δ 167.2, 164.0 (dd, *J* = 253.1, 11.9 Hz), 160.5 (dd, *J* = 253.4, 12.1 Hz), 143.7, 141.6, 135.0 (dd, *J* = 10.1, 3.2 Hz), 134.5, 133.7, 125.9, 123.4, 122.1, 119.7 (dd, *J* = 15.1, 3.6 Hz), 111.5 (dd, *J* = 21.3, 3.5 Hz), 104.3 (t, *J* = 25.1 Hz), 103.3, 39.4, 22.2, 12.9. ^19^F NMR (565 MHz, CDCl_3_) δ −105.54–−105.48 (m, 1F), −107.30–−105.25 (m, 1F). Mass spectrometry: HRMS (ESI-TOF) (*m*/*z*): Calcd for C_18_H_14_F_2_INNaO_3_S^+^ ([M + Na]^+^), 511.9599, found, 511.9610.

(*E*)-3-((2-iodo-1-(naphthalen-2-yl)pent-1-en-1-yl)oxy)benzo[*d*]isothiazole 1,1-dioxide **3k**

**1-15** (0.2 mmol, 38.8 mg) and **2a** (0.24 mmol, 74.1 mg) were employed. **3k** (PE/EtOAc = 5:1, Rf = 0.3) was purified by column chromatography on silica gel (PE/EtOAc = 10:1), white solid (65.6 mg, 65%), crystallization in CDCl_3_, mp. 146–148 °C. NMR spectroscopy: ^1^H NMR (600 MHz, CDCl_3_) δ 8.10 (s, 1H), 7.86–7.80 (m, 5H), 7.74–7.70 (m, 2H), 7.67 (dd, *J* = 8.4, 1.2 Hz, 1H), 7.51–7.47 (m, 2H), 2.62 (t, *J* = 7.2 Hz, 2H), 1.74–1.68 (m, 2H), 1.01 (t, *J* = 7.2 Hz, 3H). ^13^C NMR {^1^H} (150 MHz, CDCl_3_) δ 167.4, 146.9, 143.7, 134.3, 133.6, 133.5, 132.6, 132.5, 130.3, 128.5, 127.9, 127.7, 127.2, 127.1, 126.4, 126.0, 123.3, 122.1, 99.1, 39.9, 22.3, 13.1. Mass spectrometry: HRMS (ESI-TOF) (*m*/*z*): Calcd for C_22_H_18_INNaO_3_S^+^ ([M + Na]^+^), 525.9944, found, 525.9949.

(*E*)-3-((2-iodo-1-(thiophen-2-yl)pent-1-en-1-yl)oxy)benzo[*d*]isothiazole 1,1-dioxide **3l**

**1-16** (0.2 mmol, 30.0 mg) and **2a** (0.24 mmol, 74.1 mg) were employed. **3l** (PE/EtOAc = 5:1, Rf = 0.3) was purified by column chromatography on silica gel (PE/EtOAc = 10:1), browm solid (56.3 mg, 61%), crystallization in CDCl_3_, mp. 103–105 °C. NMR spectroscopy: ^1^H NMR (600 MHz, CDCl_3_) δ 7.89 (d, *J* = 7.8 Hz, 1H), 7.86 (d, *J* = 7.8 Hz, 1H), 7.80 (td, *J* = 7.8, 1.2 Hz, 1H), 7.76 (td, *J* = 7.8, 1.2 Hz, 1H), 7.49 (dd, *J* = 4.2, 1.2 Hz, 1H), 7.38 (dd, *J* = 4.8, 1.2 Hz, 1H), 7.03 (dd, *J* = 4.8, 4.2 Hz, 1H), 2.61–2.59 (m, 2H), 1.71–1.64 (m, 2H), 0.95 (t, *J* = 7.2 Hz, 3H). ^13^C NMR {^1^H} (150 MHz, CDCl_3_) δ 167.4, 144.0, 141.5, 135.6, 134.5, 133.7, 131.1, 127.9, 126.8, 125.9, 123.3, 122.2, 99.9, 40.7, 22.5, 13.1. Mass spectrometry: HRMS (ESI-TOF) (*m*/*z*): Calcd for C_16_H_14_INNaO_3_S_2_^+^ ([M + Na]^+^), 481.9352, found, 481.9355.

(*E*)-3-((2-iodo-1-phenylprop-1-en-1-yl)oxy)benzo[*d*]isothiazole 1,1-dioxide **3m**

**1-17** (0.2 mmol, 42.7 mg) and **2a** (0.24 mmol, 74.1 mg) were employed. **3m** (PE/EtOAc = 5:1, Rf = 0.3) was purified by column chromatography on silica gel (PE/EtOAc = 10:1), white solid (60.9 mg, 72%), crystallization in CDCl_3_, mp. 154–156 °C. NMR spectroscopy: ^1^H NMR (600 MHz, CDCl_3_) δ 7.85 (d, *J* = 7.8 Hz, 1H), 7.82 (d, *J* = 7.8 Hz, 1H), 7.76 (td, *J* = 7.2, 1.2 Hz, 1H), 7.72 (td, *J* = 7.8, 1.2 Hz, 1H), 7.61–7.59 (m, 2H), 7.39–7.36 (m, 3H), 2.59 (s, 3H). ^13^C NMR {^1^H} (150 MHz, CDCl_3_) δ 167.2, 147.1, 143.8, 135.0, 134.4, 133.6, 130.1, 129.8, 128.2, 126.0, 123.4, 122.1, 89.0, 27.3. Mass spectrometry: HRMS (ESI-TOF) (*m*/*z*): Calcd for C_16_H_12_INNaO_3_S^+^ ([M + Na]^+^), 447.9475, found, 447.9468.

(*E*)-3-((6-chloro-2-iodo-1-(p-tolyl)hex-1-en-1-yl)oxy)benzo[*d*]isothiazole 1,1-dioxide **3n**

**1-20** (0.2 mmol, 41.3 mg) and **2a** (0.24 mmol, 74.1 mg) were employed. **3n** (PE/EtOAc = 5:1, Rf = 0.3) wa purified by column chromatography on silica gel (PE/EtOAc = 10:1), yellow oil (77.3 mg, 75%). NMR spectroscopy: ^1^H NMR (600 MHz, CDCl_3_) δ 7.85 (d, *J* = 7.2 Hz, 1H), 7.82 (d, *J* = 7.2 Hz, 1H), 7.77 (td, *J* = 7.2, 1.2 Hz, 1H), 7.72 (td, *J* = 7.2, 1.8 Hz, 1H), 7.48 (d, *J* = 8.4 Hz, 2H), 7.18 (d, *J* = 7.8 Hz, 2H), 3.55 (t, *J* = 6.0 Hz, 2H), 2.63–2.62 (m, 2H), 2.35 (s, 3H), 1.84–1.82 (m, 4H). ^13^C NMR {^1^H} (150 MHz, CDCl_3_) δ 167.3, 147.4, 143.8, 130.0, 134.4, 133.6, 132.2, 130.1, 128.9, 126.0, 123.3, 122.1, 97.2, 44.6, 37.3, 31.2, 26.2, 21.4. Mass spectrometry: HRMS (ESI-TOF) (*m*/*z*): Calcd for C_20_H_19_ClINNaO_3_S^+^ ([M + Na]^+^), 537.9711, found, 537.9705.

(*E*)-3-((3-chloro-2-iodo-1-phenylprop-1-en-1-yl)oxy)benzo[*d*]isothiazole 1,1-dioxide **3o**

**1-21** (0.2 mmol, 30.0 mg) and **2a** (0.24 mmol, 74.1 mg) were employed. **3o** (PE/EtOAc = 5:1, Rf = 0.3) was purified by column chromatography on silica gel (PE/EtOAc = 10:1), white solid (84.4 mg, 92%), crystallization in CDCl_3_, mp. 146–148 °C. NMR spectroscopy: ^1^H NMR (600 MHz, CDCl_3_) δ 7.88 (t, *J* = 7.8 Hz, 2H), 7.80–7.79 (m, 1H), 7.76 (dt, *J* = 7.2, 1.2 Hz, 1H), 7.64–7.62 (m, 2H), 7.41–7.39 (m, 3H), 4.53 (s, 2H).^13^C NMR {^1^H} (150 MHz, CDCl_3_) δ 167.2, 150.5, 143.8, 134.6, 133.8, 133.8, 130.6, 129.9, 128.4, 125.6, 123.5, 122.3, 90.5, 48.3. Mass spectrometry: HRMS (ESI-TOF) (*m*/*z*): Calcd for C_16_H_11_ClINNaO_3_S_2_^+^ ([M + Na]^+^), 480.9085, found 481.9071.

(*E*)-3-((2-cyclopropyl-2-iodo-1-(p-tolyl)vinyl)oxy)benzo[*d*]isothiazole 1,1-dioxide **3p**

**1-23** (0.2 mmol, 42.7 mg) and **2a** (0.24 mmol, 74.1 mg) were employed. **3p** (PE/EtOAc = 5:1, Rf = 0.3) was purified by column chromatography on silica gel (PE/EtOAc = 10:1), white solid (80.4 mg, 86%), crystallization in CDCl_3_, mp. 103–105 °C. NMR spectroscopy: ^1^H NMR (600 MHz, CDCl_3_) δ 7.86 (d, *J* = 7.2 Hz, 1H), 7.84 (d, *J* = 7.8 Hz, 1H), 7.76 (t, *J* = 7.2 Hz, 1H), 7.72 (t, *J* = 7.8 Hz, 1H), 7.48 (d, *J* = 7.8 Hz, 2H), 7.18 (d, *J* = 7.8 Hz, 2H), 2.35 (s, 3H), 1.54–1.49 (m, 1H), 0.83 (d, *J* = 6.6 Hz, 4H). ^13^C NMR {^1^H} (150 MHz, CDCl_3_) δ 167.3, 147.8, 143.9, 139.8, 134.2, 133.5, 132.9, 130.1, 128.9, 126.4, 123.4, 122.1, 103.3, 21.4, 16.9, 10.0. Mass spectrometry: HRMS (ESI-TOF) (*m*/*z*): Calcd for C_19_H_16_INNaO_3_S^+^ ([M + Na]^+^), 487.9788, found, 487.9790.

(*E*)-3-((3-ethoxy-2-iodo-1-phenylprop-1-en-1-yl)oxy)benzo[*d*]isothiazole 1,1-dioxide **3q**

**1-24** (0.2 mmol, 32.0 mg) and **2a** (0.24 mmol, 74.1 mg) were employed. **3q** (PE/EtOAc = 5:1, Rf = 0.3) was purified by column chromatography on silica gel (PE/EtOAc = 10:1), white solid (80.0 mg, 85%), crystallization in CDCl_3_, mp. 162–164 °C. NMR spectroscopy: ^1^H NMR (600 MHz, CDCl_3_) δ 7.87 (d, *J* = 7.8 Hz, 1H), 7.83 (d, *J* = 7.8 Hz, 1H), 7.78 (td, *J* = 7.8, 1.2 Hz, 1H), 7.73 (td, *J* = 7.2, 1.2 Hz, 1H), 7.63–7.60 (m, 2H), 7.41–7.38 (m, 3H), 4.33 (s, 2H), 3.54 (q, *J* = 7.2 Hz, 2H), 1.20 (t, *J* = 7.2 Hz, 3H). ^13^C NMR {^1^H} (150 MHz, CDCl_3_) δ 167.4, 148.9, 143.9, 134.7, 134.5, 133.6, 130.1, 130.0, 128.3, 126.0, 123.3, 122.2, 95.2, 71.9, 66.0, 15.0. Mass spectrometry: HRMS (ESI-TOF) (*m*/*z*): Calcd for C_18_H_16_INNaO_4_S^+^ ([M + Na]^+^), 491.9737, found 491.9750.

(*E*)-3-((2-iodo-1,2-diphenylvinyl)oxy)benzo[*d*]isothiazole 1,1-dioxide **3r**

**1-25** (0.2 mmol, 35.6 mg) and **2a** (0.24 mmol, 74.1 mg) were employed. **3r** (PE/EtOAc = 5:1, Rf = 0.3) was purified by column chromatography on silica gel (PE/EtOAc = 10:1), white solid (51.3 mg, 53%), crystallization in CDCl_3_, mp. 172–174 °C. NMR spectroscopy: ^1^H NMR (600 MHz, CDCl_3_) δ 7.76 (d, *J* = 7.2 Hz, 3H), 7.66 (t, *J* = 7.2 Hz, 1H), 7.57 (t, *J* = 7.2 Hz, 1H), 7.52 (d, *J* = 7.2 Hz, 1H), 7.48 (d, *J* = 7.2 Hz, 2H), 7.44–7.40 (m, 3H), 7.28–7.26 (m, 2H), 7.17 (t, *J* = 7.2 Hz, 1H). ^13^C NMR {^1^H} (150 MHz, CDCl_3_) δ 167.7, 147.5, 143.6, 139.6, 134.8, 134.1, 133.3, 130.2, 130.1, 128.8, 128.42, 128.39, 128.2, 125.9, 123.1, 121.9, 91.2. Mass spectrometry: HRMS (ESI-TOF) (*m*/*z*): Calcd for C_21_H_14_INNaO_3_S^+^ ([M + Na]^+^), 509.9631, found, 509.9638.

(*E*)-3-((4-iodohex-3-en-3-yl)oxy)benzo[*d*]isothiazole 1,1-dioxide **3s**

**1-26** (0.24 mmol, 19.7 mg) and **2a** (0.2 mmol, 61.8 mg) were employed. **3s** (PE/EtOAc = 5:1, Rf = 0.3) was purified by column chromatography on silica gel (PE/EtOAc = 10:1), yellow oil (51.0 mg, 65%). NMR spectroscopy: ^1^H NMR (600 MHz, CDCl_3_) δ 7.92 (d, *J* = 7.2 Hz, 1H), 7.82 (t, *J* = 7.2 Hz, 2H), 7.77 (t, *J* = 7.2 Hz, 1H), 2.79 (q, *J* = 7.2 Hz, 2H), 2.42 (q, *J* = 7.2 Hz, 2H), 1.12 (t, *J* = 7.2 Hz, 3H), 1.07 (t, *J* = 7.2 Hz, 3H). ^13^C NMR {^1^H} (150 MHz, CDCl_3_) δ 167.3, 149.7, 143.9, 134.4, 133.6, 126.1, 123.3, 122.1, 97.8, 31.2, 28.9, 13.9, 10.9. Spectrometry: HRMS (ESI-TOF) (*m*/*z*): Calcd for C_13_H_14_INNaO_3_S^+^ ([M+ Na]^+^), 413.9631, found, 413.9638.

(*E*)-3-((5-iodooct-4-en-4-yl)oxy)benzo[*d*]isothiazole 1,1-dioxide **3t**

**1-27** (0.24 mmol, 26.4 mg) and **2a** (0.2 mmol, 61.8 mg) were employed. **3t** (PE/EtOAc = 5:1, Rf = 0.3) was purified by column chromatography on silica gel (PE/EtOAc = 10:1), white solid (55.9 mg, 67%), crystallization in CDCl_3_, mp. 58–60 °C. NMR spectroscopy: ^1^H NMR (600 MHz, CDCl_3_) δ 7.92 (d, *J* = 7.8 Hz, 1H), 7.83–7.80 (m, 2H), 7.76 (t, *J* = 7.2 Hz, 1H), 2.78 (t, *J* = 7.2 Hz, 2H), 2.39 (t, *J* = 7.2 Hz, 2H), 1.62–1.53 (m, 4H), 0.99 (t, *J* = 7.2 Hz, 3H), 0.88 (t, *J* = 7.8 Hz, 3H). ^13^C NMR {^1^H} (150 MHz, CDCl_3_) δ 167.1, 149.6, 143.9, 134.4, 133.6, 126.2, 123.2, 122.2, 97.1, 39.2, 36.9, 22.2, 20.1, 13.4, 12.9. Spectrometry: HRMS (ESI-TOF) (*m*/*z*): Calcd for C_15_H_18_INNaO_3_S^+^ ([M+ Na]^+^), 441.9944, found, 441.9930.

(*E*)-3-((1-(4-(*tert*-butyl)phenyl)-2-iodovinyl)oxy)benzo[*d*]isothiazole 1,1-dioxide **3u**

**1-29** (0.24 mmol, 37.9 mg) and **2a** (0.2 mmol, 61.8 mg) were employed. **3u** (PE/EtOAc = 5:1, Rf = 0.3) was purified by column chromatography on silica gel (PE/EtOAc = 10:1), white solid (56.9 mg, 61%), crystallization in CDCl_3_, mp. 151–153 °C. NMR spectroscopy: ^1^H NMR (600 MHz, CDCl_3_) δ 7.88 (d, *J* = 7.8 Hz, 1H), 7.84 (d, *J* = 7.8 Hz, 1H), 7.78 (td, *J* = 7.2, 1.8 Hz, 1H), 7.74 (td, *J* = 7.8, 1.2 Hz, 1H), 7.64 (d, *J* = 8.4 Hz, 2H), 7.42 (d, *J* = 9.0 Hz, 2H), 6.77 (s, 1H), 1.32 (s, 9H). ^13^C NMR {^1^H} (150 MHz, CDCl_3_) δ 167.9, 153.6, 151.7, 143.6, 134.4, 133.6, 129.4, 128.9, 126.5, 125.4, 123.4, 122.1, 68.7, 34.9, 31.1. Mass Spectrometry: HRMS (ESI-TOF) (*m*/*z*): Calcd for C_19_H_18_INNaO_3_S^+^ ([M + Na]^+^), 489.9944, found, 489.9953.

(*E*)-3-((1-([1,1′-biphenyl]-4-yl)-2-iodovinyl)oxy)benzo[*d*]isothiazole 1,1-dioxide **3v**

**1-30** (0.24 mmol, 42.7 mg) and **2a** (0.2 mmol, 61.8 mg) were employed. **3v** (PE/EtOAc = 5:1, Rf = 0.3) was purified by column chromatography on silica gel (PE/EtOAc = 10:1), white solid (66.1 mg, 68%), crystallization in CDCl_3_, mp. 161–162 °C. NMR spectroscopy: ^1^H NMR (600 MHz, CDCl_3_) δ 7.88 (d, *J* = 7.5 Hz, 1H), 7.86 (d, *J* = 7.5 Hz, 1H), 7.78–7.74 (m, 4H), 7.64 (d, *J* = 8.1 Hz, 2H), 7.60 (d, *J* = 7.9 Hz, 2H), 7.44 (t, *J* = 7.6 Hz, 2H), 7.37 (t, *J* = 7.2 Hz, 1H), 6.85 (s, 1H). ^13^C NMR {^1^H} (150 MHz, CDCl_3_) δ 167.9, 151.5, 143.6, 143.1, 140.1, 134.4, 133.6, 131.2, 129.6, 128.9, 127.9, 127.2, 127.1, 126.4, 123.4, 122.2, 69.5. Mass Spectrometry: HRMS (ESI-TOF) (*m*/*z*): Calcd for C_21_H_14_INNaO_3_S^+^ ([M + Na]^+^), 509.9631, found, 509.9640.

3-((1-iodo-4-phenylbut-3-yn-2-yl)oxy)benzo[*d*]isothiazole 1,1-dioxide **4a**

**1-31** (0.24 mmol, 30.7 mg) and **2a** (0.2 mmol, 61.8 mg) were employed. **4a** (PE/EtOAc = 5:1, Rf = 0.32) was purified by column chromatography on silica gel (PE/EtOAc = 10:1), colorless oil (36.4 mg, 42%). NMR spectroscopy: ^1^H NMR (500 MHz, CDCl_3_) δ 7.91 (d, *J* = 7.6 Hz, 1H), 7.85 (d, *J* = 7.5 Hz, 1H), 7.80 (t, *J* = 7.5 Hz, 1H), 7.75 (t, *J* = 7.5 Hz, 1H), 7.51 (d, *J* = 6.6 Hz, 2H), 7.41–7.31 (m, 3H), 6.04 (t, *J* = 5.8 Hz, 1H), 3.73 (d, *J* = 5.9 Hz, 2H). ^13^C NMR {^1^H} (150 MHz, CDCl_3_) δ 167.91, 143.66, 134.42, 133.61, 132.17, 129.55, 128.44, 126.51, 123.72, 122.10, 120.98, 89.09, 82.67, 71.25, 3.99. HRMS (ESI-TOF) (*m*/*z*): C_17_H_12_INNaO_3_S^+^ Calcd for, ([M + Na]^+^), 459.9480 found 459.9474.

The reaction tube equipped with a magnetic stir bar was charged with **1-32** (0.2 mmol, 20.8 mg), **1-17** (0.2 mmol, 23.2 mg), **2a** (0.2 mmol, 61.8 mg), and DCM (2 mL). The test tube was then sealed off with a screw cap, and the reaction mixture was stirred at room temperature for 6.0 or 24 h. After the reaction was completed, as indicated by TLC analysis, the mixture was extracted with DCM (2 × 10 mL). The combined organic phases were dried over anhydrous Na_2_SO_4_, and the solvent was evaporated under a vacuum. The residue was purified by column chromatography (petroleum ether/ethyl acetate 15:1 (*v*/*v*)) to give the corresponding product **4b** (36.4 mg, 54%) and **4b′** (16.2 mg, 20%).

3-(2-iodo-1-phenylethoxy)benzo[*d*]isothiazole 1,1-dioxide **4b**

**4b** (PE/EtOAc = 5:1, Rf = 0.28), colorless oil. NMR spectroscopy: ^1^H NMR (500 MHz, CDCl_3_) δ 7.91–7.82 (m, 2H), 7.79–7.72 (m, 2H), 7.51–7.38 (m, 5H), 6.28–6.16 (m, 1H), 3.77 (dd, *J* = 10.8, 7.6 Hz, 1H), 3.66 (dd, *J* = 10.8, 5.7 Hz, 1H). ^13^C NMR {^1^H} (150 MHz, CDCl_3_) δ 168.1, 143.5, 136.1, 134.3, 133.5, 129.7, 128.9, 126.8, 126.7, 123.4, 122.0, 82.7, 5.5. HRMS (ESI-TOF) (*m*/*z*): C_15_H_12_INNaO_3_S^+^ Calcd for, ([M + Na]^+^), 435.9475 found 435.9485.

2-(2-iodo-1-phenylethyl)benzo[*d*]isothiazol-3(2H)-one 1,1-dioxide **4b′**

**4b′** (PE/EtOAc = 5:1, Rf = 0.32), colorless oil. NMR spectroscopy: ^1^H NMR (500 MHz, CDCl_3_) δ 8.04 (d, *J* = 6.8 Hz, 1H), 7.90–7.80 (m, 3H), 7.60 (d, *J* = 6.6 Hz, 2H), 7.47–7.31 (m, 3H), 5.42 (t, *J* = 8.2 Hz, 1H), 4.32 (dd, *J* = 10.5, 8.5 Hz, 1H), 4.03 (dd, *J* = 10.5, 7.8 Hz, 1H). ^13^C NMR {^1^H} (150 MHz, CDCl_3_) δ 158.6, 137.3, 135.6, 134.9, 134.4, 129.2, 128.8, 128.5, 127.0, 125.3, 120.9, 59.1, 2.3. HRMS (ESI-TOF) (*m*/*z*): C_15_H_12_INNaO_3_S^+^ Calcd for, ([M + Na]^+^), 435.9475 found 435.9468.

### 4.3. The General Procedure for the Synthesis of ***5***

In a nitrogen-filled glove box, a flame-dried screw cap reaction tube equipped with a magnetic stir bar was charged with NCBSI (0.4 mmol, 132.4 mg), CHCl_3_ (3 mL), and **1** (0.2 mmol). The test tube was then sealed off with a screw cap and removed from the glove box, and the reaction mixture was stirred at room temperature for 24 h. After the reaction was completed, as indicated by TLC analysis, the mixture was extracted using DCM (3 × 5.0 mL). The combined organic phases were dried over anhydrous Na_2_SO_4_, and the solvent was evaporated under a vacuum. The residue was purified via column chromatography (petroleum ether/ethyl acetate 30:1 (*v*/*v*)) to provide the corresponding product **5**.

(*E*)-2-chloro-1-phenylprop-1-en-1-yl *N*-phenylsulfonylbenzenesulfonimidate **5a**

**1-17** (0.2 mmol, 23.2 mg) and **2b** (0.4 mmol, 132.4 mg) were employed. **5a** (PE/EtOAc = 10:1, Rf = 0.3) was purified by column chromatography on silica gel (PE/EtOAc = 30:1), white solid (76.7 mg, 86%), crystallization in CDCl_3_, mp. 87–88 °C. NMR spectroscopy: ^1^H NMR (600 MHz, CDCl_3_) *δ* 7.96 (d, *J* = 7.2 Hz, 2H), 7.60 (d, *J* = 7.8 Hz, 2H), 7.54 (t, *J* = 7.2 Hz, 1H), 7.48–7.44 (m, 3H), 7.25 (d, *J* = 7.8 Hz, 2H), 7.17–7.14 (m, 2H), 7.13 (t, *J* = 7.8 Hz, 1H), 7.06 (t, *J* = 7.8 Hz, 2H), 2.41 (s, 3H). ^13^C NMR {^1^H} (150 MHz, CDCl_3_) *δ* 142.8, 142.3, 135.9, 134.3, 132.5, 131.1, 129.5, 129.5, 129.0, 128.8, 128.7, 127.9, 127.7, 126.7, 21.8. HRMS (ESI-TOF) (*m*/*z*): Calcd for C_21_H_18_ClNNaO_4_S_2_^+^, ([M + Na]^+^), 470.0258, found 470.0265.

(*E*)-2-chloro-1-phenylbut-1-en-1-yl *N*-phenylsulfonylbenzenesulfonimidate **5b**

**1-18** (0.2 mmol, 26.0 mg) and **2b** (0.4 mmol, 132.4 mg) were employed. **5b** (PE/EtOAc = 10:1, Rf = 0.3) was purified by column chromatography on silica gel (PE/EtOAc = 30:1), white solid (74.0 mg, 80%), crystallization in CDCl_3_, mp. 98–99 °C. NMR spectroscopy: ^1^H NMR (600 MHz, CDCl_3_) δ 7.96 (d, *J* = 7.2 Hz, 2H), 7.60 (d, *J* = 7.2 Hz, 2H), 7.54 (t, *J* = 7.2 Hz, 1H), 7.48–7.44 (m, 3H), 7.27–7.23 (m, 2H), 7.17–7.14 (m, 2H), 7.13 (t, *J* = 7.2 Hz, 1H), 7.06 (t, *J* = 7.2 Hz, 2H), 2.89–2.83 (m, 1H), 2.72–2.66 (m, 1H), 1.21 (t, *J* = 7.2 Hz, 3H). ^13^C NMR {^1^H} (125 MHz, CDCl_3_) δ 142.8, 141.4, 136.0, 135.4, 134.3, 132.5, 131.2, 129.7, 129.0, 128.8, 128.7, 127.9, 127.7, 126.7, 27.3, 11.9. HRMS (ESI-TOF) (*m*/*z*): Calcd for C_22_H_20_ClNNaO_4_S_2_^+^, ([M + Na]^+^), 484.0414, found 484.0426.

(*E*)-2-chloro-1-phenylpent-1-en-1-yl *N*-phenylsulfonylbenzenesulfonimidate **5c**

**1-1** (0.2 mmol, 28.8 mg) and **2b** (0.4 mmol, 132.4 mg) were employed. **5c** (PE/EtOAc = 10:1, Rf = 0.3) was purified by column chromatography on silica gel (PE/EtOAc = 30:1), white solid (76.6 mg, 81%), crystallization in CDCl_3_, mp. 101–102 °C. NMR spectroscopy: ^1^H NMR (600 MHz, CDCl_3_) δ 7.98–7.94 (m, 2H), 7.59 (dd, *J* = 8.4, 0.6 Hz, 2H), 7.55–7.51 (m, 1H), 7.48–7.44 (m, 3H), 7.26–7.23 (m, 2H), 7.17–7.14 (m, 2H), 7.14–7.11 (m, 1H), 7.05 (t, *J* = 7.2 Hz, 2H), 2.78–2.67 (m, 2H), 1.73–1.61 (m, 2H), 0.99 (t, *J* = 7.2 Hz, 3H). ^13^C NMR {^1^H} (150 MHz, CDCl_3_) δ 142.9, 142.1, 136.2, 134.3, 134.0, 132.4, 131.2, 129.7, 129.0, 128.8, 128.7, 127.9, 127.7, 126.7, 35.3, 20.5, 13.3. HRMS (ESI-TOF) (*m*/*z*): Calcd for C_23_H_22_ClNNaO_4_S_2_^+^, ([M + Na]^+^), 498.0571, found 498.0571.

(*E*)-2-chloro-3,3-dimethyl-1-phenylbut-1-en-1-yl *N*-phenylsulfonylbenzenesulfonimidate **5d**

**1-19** (0.2 mmol, 31.6 mg) and **2b** (0.4 mmol, 132.4 mg) were employed. **5d** (PE/EtOAc = 10:1, Rf = 0.33) was purified by column chromatography on silica gel (PE/EtOAc = 30:1), white solid (81.5 mg, 83%), crystallization in CDCl_3_, mp. 101–102 °C. NMR spectroscopy: ^1^H NMR (600 MHz, CDCl_3_) δ 7.91–7.88 (m, 2H), 7.73–7.69 (m, 2H), 7.53–7.50 (m, 2H), 7.43 (t, *J* = 7.6 Hz, 2H), 7.34 (t, *J* = 7.6 Hz, 2H), 7.26 (d, *J* = 7.2 Hz, 1H), 7.21 (d, *J* = 7.2 Hz, 2H), 7.14 (t, *J* = 7.2 Hz, 2H), 1.02 (s, 9H). ^13^C NMR {^1^H} (150 MHz, CDCl_3_) δ 142.9, 141.9, 140.6, 137.9, 134.0, 132.7, 132.2, 131.2, 129.8, 128.8, 128.5, 127.8, 127.7, 126.8, 38.2, 30.5. HRMS (ESI-TOF) (*m*/*z*): Calcd for C_24_H_24_ClNNaO_4_S_2_^+^, ([M + Na]^+^), 512.0727, found 512.0723.

(*E*)-2-chloro-2-cyclopropyl-1-phenylvinyl *N*-phenylsulfonylbenzenesulfonimidate **5e**

**1-22** (0.2 mmol, 28.4 mg) and **2b** (0.4 mmol, 132.4 mg) were employed. **5e** (PE/EtOAc = 10:1, Rf = 0.26) was purified by column chromatography on silica gel (PE/EtOAc = 30:1), yellow solid (61.1 mg, 65%), crystallization in CDCl_3_, mp. 99–100 °C. NMR spectroscopy: ^1^H NMR (600 MHz, CDCl_3_) δ 7.96 (dd, *J* = 7.8, 1.2 Hz, 2H), 7.65–7.62 (m, 2H), 7.55–7.53 (m, 1H), 7.49–7.45 (m, 3H), 7.26 (t, *J* = 7.8 Hz, 2H), 7.18–7.15 (m, 2H), 7.13–7.11 (m, 1H), 7.07 (t, *J* = 7.2 Hz, 2H), 2.64–2.60 (m, 1H), 0.97–0.93 (m, 1H), 0.92–0.86 (m, 2H), 0.82–0.78 (m, 1H). ^13^C NMR {^1^H} (150 MHz, CDCl_3_) δ 142.9, 142.1, 136.1, 135.2, 134.3, 132.4, 131.8, 129.6, 128.8, 128.7, 128.0, 127.7, 126.7, 13.2, 6.5, 6.2. HRMS (ESI-TOF) (*m*/*z*): Calcd for C_23_H_20_ClNNaO_4_S_2_^+^, ([M + Na]^+^), 496.0414, found 496.0413.

(*E*)-2-chloro-2-cyclopropyl-1-(p-tolyl)vinyl *N*-(phenylsulfonyl)benzenesulfonimidate **5f**

**1-23** (0.2 mmol, 31.2 mg) and **2b** (0.4 mmol, 132.4 mg) were employed. **5f** (PE/EtOAc = 10:1, Rf = 0.28) was purified by column chromatography on silica gel (PE/EtOAc = 30:1), yellow oil (56.7 mg, 58%). NMR spectroscopy: ^1^H NMR (500 MHz, CDCl_3_) δ 7.96 (d, *J* = 7.5 Hz, 2H), 7.64 (d, *J* = 7.5 Hz, 2H), 7.54 (t, *J* = 7.5 Hz, 1H), 7.51–7.42 (m, 3H), 7.28 (t, *J* = 7.5 Hz, 2H), 7.05 (d, *J* = 8.0 Hz, 2H), 6.87 (d, *J* = 8.0 Hz, 2H), 2.60–2.55 (m, 1H), 2.24 (s, 3H), 0.97–0.81 (m, 3H), 0.80–0.74 (m, 1H). ^13^C NMR {^1^H} (150 MHz, CDCl_3_) δ 143.0, 142.3, 138.9, 136.3, 134.6, 134.1, 132.4, 129.6, 129.0, 128.8, 128.7, 128.4, 128.1, 126.8, 21.3, 13.2, 6.5, 6.1. C_24_H_22_ClNNaO_4_S_2_^+^ Calcd for, ([M + Na]^+^), 510.0571, found 510.0588.

(*E*)-2-chloro-1-phenylvinyl *N*-phenylsulfonylbenzenesulfonimidate **5g**

**1-28** (0.2 mmol, 20.4 mg) and **2b** (0.4 mmol, 132.4 mg) were employed. **5g** (PE/EtOAc = 10:1, Rf = 0.25) was purified by column chromatography on silica gel (PE/EtOAc = 30:1), white solid (69.8 mg, 81%), crystallization in CDCl_3_, mp. 66–67 °C. NMR spectroscopy: ^1^H NMR (600 MHz, CDCl_3_) δ 8.01 (d, *J* = 7.8 Hz, 2H), 7.72 (d, *J* = 7.2 Hz, 2H), 7.58–7.53 (m, 2H), 7.50 (t, *J* = 7.8 Hz, 2H), 7.36–7.30 (m, 4H), 7.23 (t, *J* = 7.2 Hz, 1H), 7.16 (t, *J* = 7.8 Hz, 2H), 6.68 (s, 1H). ^13^C NMR {^1^H} (150 MHz, CDCl_3_) δ 147.2, 142.7, 135.2, 134.7, 132.6, 129.8, 129.8, 129.0, 128.8, 128.6, 128.0, 128.0, 126.8, 116.0. HRMS (ESI-TOF) (*m*/*z*): Calcd for C_20_H_16_ClNNaO_4_S_2_^+^, ([M + Na]^+^), 456.0101, found 456.0102.

(*E*)-2-chloro-1-(p-tolyl)pent-1-en-1-yl *N*-phenylsulfonylbenzenesulfonimidate **5h**

**1-2** (0.2 mmol, 31.6 mg) and **2b** (0.4 mmol, 132.4 mg) were employed. **5h** (PE/EtOAc = 10:1, Rf = 0.3) was purified by column chromatography on silica gel (PE/EtOAc = 30:1), colorless liquid (86.2 mg, 88%). NMR spectroscopy: ^1^H NMR (600 MHz, CDCl_3_) δ 7.95 (d, *J* = 7.2 Hz, 2H), 7.60 (dd, *J* = 8.4, 1.2 Hz, 2H), 7.53 (t, *J* = 7.2 Hz, 1H), 7.49–7.44 (m, 3H), 7.26 (d, *J* = 8.4 Hz, 2H), 7.04 (d, *J* = 7.8 Hz, 2H), 6.85 (d, *J* = 8.4 Hz, 2H), 2.72–2.63 (m, 2H), 2.23 (s, 3H), 1.70–1.59 (m, 2H), 0.98 (t, *J* = 7.2 Hz, 3H). ^13^C NMR {^1^H} (150 MHz, CDCl_3_) δ 142.9, 142.3, 139.1, 136.4, 134.0, 133.3, 132.4, 129.7, 128.8, 128.7, 128.3, 127.9, 126.7, 35.3, 21.3, 20.5, 13.3. HRMS (ESI-TOF) (*m*/*z*): Calcd for C_24_H_24_ClNNaO_4_S_2_^+^, ([M + Na]^+^), 512.0727, found 512.0727.

(*E*)-1-([1,1′-biphenyl]-4-yl)-2-chloropent-1-en-1-yl *N*-phenylsulfonylbenzenesulfonimidate **5i**

**1-9** (0.2 mmol, 44.0 mg) and **2b** (0.4 mmol, 132.4 mg) were employed. **5i** (PE/EtOAc = 10:1, Rf = 0.32) was purified by column chromatography on silica gel (PE/EtOAc = 30:1), white solid (96.7 mg, 88%), crystallization in CDCl_3_, mp. 99–100 °C. NMR spectroscopy: ^1^H NMR (600 MHz, CDCl_3_) δ 7.97 (d, *J* = 7.2 Hz, 2H), 7.62 (d, *J* = 7.2 Hz, 2H), 7.53 (t, *J* = 7.2 Hz, 1H), 7.47–7.42 (m, 7H), 7.38–7.35 (m, 1H), 7.26–7.21 (m, 6H), 2.82–2.71 (m, 2H), 1.76–1.65 (m, 2H), 1.01 (t, *J* = 7.2 Hz, 3H). ^13^C NMR {^1^H} (150 MHz, CDCl_3_) δ 142.8, 142.0 141.7, 140.1, 136.3, 134.1, 134.0, 132.5, 130.2, 130.1, 128.9, 128.8, 128.7, 128.0, 127.8, 126.9, 126.7, 126.3, 35.4, 20.5, 13.3. HRMS (ESI-TOF) (*m*/*z*): Calcd for C_29_H_26_ClNNaO_4_S_2_^+^, ([M + Na]^+^), 574.0884, found 574.0893.

(*E*)-2-chloro-1-(4-chlorophenyl)pent-1-en-1-yl *N*-phenylsulfonylbenzenesulfonimidate **5j**

**1-10** (0.2 mmol, 35.6 mg) and **2b** (0.4 mmol, 132.4 mg) were employed. **5j** (PE/EtOAc = 10:1, Rf = 0.32) was purified by column chromatography on silica gel (PE/EtOAc = 30:1), white solid (72.7 mg, 71%), crystallization in CDCl_3_, mp. 104–105 °C. NMR spectroscopy: ^1^H NMR (600 MHz, CDCl_3_) δ 7.94 (d, *J* = 7.2 Hz, 2H), 7.63 (d, *J* = 7.2 Hz, 2H), 7.57–7.53 (m, 2H), 7.47 (t, *J* = 7.8 Hz, 2H), 7.32 (t, *J* = 7.2 Hz, 2H), 7.11 (d, *J* = 8.4 Hz, 2H), 7.03 (d, *J* = 8.4 Hz, 2H), 2.75–2.63 (m, 2H), 1.69–1.59 (m, 2H), 0.98 (t, *J* = 7.2 Hz, 3H). ^13^C NMR {^1^H} (150 MHz, CDCl_3_) δ 142.7, 141.0, 136.2, 135.0, 134.5, 134.4, 132.5, 131.1, 129.8, 129.0, 128.7, 127.9, 127.9, 126.7, 35.3, 20.5, 13.2. HRMS (ESI-TOF) (*m*/*z*): Calcd for C_23_H_21_Cl_2_NNaO_4_S_2_^+^, ([M + Na]^+^), 532.0181, found 532.0185.

(*E*)-2-chloro-1-(4-bromophenyl)pent-1-en-1-yl *N*-phenylsulfonylbenzenesulfonimidate **5k**

**1-11** (0.2 mmol, 44.4 mg) and **2b** (0.4 mmol, 132.4 mg) were employed. **5k** (PE/EtOAc = 10:1, Rf = 0.32) was purified by column chromatography on silica gel (PE/EtOAc = 30:1), yellow solid (79.4 mg, 72%), crystallization in CDCl_3_, mp. 101–102 °C. NMR spectroscopy: ^1^H NMR (600 MHz, CDCl_3_) δ 7.94 (d, *J* = 7.8 Hz, 2H), 7.62 (d, *J* = 8.4 Hz, 2H), 7.55 (t, *J* = 7.8 Hz, 2H), 7.47 (t, *J* = 7.8 Hz, 2H), 7.33 (t, *J* = 7.8 Hz, 2H), 7.21–7.17 (m, 2H), 7.04 (d, *J* = 8.4 Hz, 2H), 2.76–2.63 (m, 2H), 1.71–1.59 (m, 2H), 0.98 (t, *J* = 7.2 Hz, 3H). ^13^C NMR {^1^H} (150 MHz, CDCl_3_) δ 142.7, 141.1, 136.1, 134.5, 134.4, 132.5, 131.3, 130.9, 130.3, 129.0, 128.7, 127.9, 126.7, 123.4, 35.3, 20.5, 13.2. HRMS (ESI-TOF) (*m*/*z*): Calcd for C_23_H_21_BrClNNaO_4_S_2_^+^, ([M + Na]^+^), 575.9676, found 575.9680.

(*E*)-tert-butyl 4-(2-chloro-1-((N-(phenylsulfonyl)phenylsulfonimidoyl)oxy)pent-1-en-1-yl)benzoate **5l**

**1-8** (0.2 mmol, 48.8 mg) and **2b** (0.4 mmol, 132.4 mg) were employed. **5l** (PE/EtOAc = 10:1, Rf = 0.30) was purified by column chromatography on silica gel (PE/EtOAc = 30:1), white solid (53.2 mg, 46%), crystallization in CDCl_3_, mp. 103–104 °C. NMR spectroscopy: ^1^H NMR (600 MHz, CDCl_3_) δ 7.93 (d, *J* = 7.2 Hz, 2H), 7.70 (d, *J* = 8.4 Hz, 2H), 7.65 (d, *J* = 7.8 Hz, 2H), 7.56–7.50 (m, 2H), 7.46 (t, *J* = 7.8 Hz, 2H), 7.32–7.29 (m, 2H), 7.25 (d, *J* = 8.4 Hz, 2H), 2.73–2.62 (m, 2H), 1.70–1.61 (m, 2H), 1.59 (s, 9H), 0.97 (t, *J* = 7.2 Hz, 3H). ^13^C NMR {^1^H} (150 MHz, CDCl_3_) δ 164.9, 142.7, 141.2, 136.1, 135.3, 135.0, 134.5, 132.5, 132.2, 129.5, 129.0, 128.7, 128.7, 127.9, 126.7, 81.3, 35.5, 28.1, 20.5, 13.3. HRMS (ESI-TOF) (*m*/*z*): Calcd for C_28_H_30_ClNNaO_6_S_2_^+^, ([M + Na]^+^), 598.1095, found 598.1094.

(*E*)-2-chloro-1-(4-(trifluoromethyl)phenyl)pent-1-en-1-yl *N*-phenylsulfonylbenzene sulfonimidate **5m**

**1-5** (0.2 mmol, 42.4 mg) and **2b** (0.4 mmol, 132.4 mg) were employed. **5m** (PE/EtOAc = 10:1, Rf = 0.35) was purified by column chromatography on silica gel (PE/EtOAc = 30:1), white solid (44.9 mg, 41%), crystallization in CDCl_3_, mp. 89–90 °C. NMR spectroscopy: ^1^H NMR (600 MHz, CDCl_3_) δ 7.95 (d, *J* = 7.2 Hz, 2H), 7.60 (d, *J* = 7.8 Hz, 2H), 7.55 (t, *J* = 7.2 Hz, 1H), 7.50–7.46 (m, 3H), 7.31–7.25 (m, 6H), 2.80–2.69 (m, 2H), 1.74–1.62 (m, 2H), 1.00 (t, *J* = 7.2 Hz, 3H). ^13^C NMR {^1^H} (125 MHz, CDCl_3_) δ 142.6, 140.6, 136.0, 135.5, 134.9, 134.6, 132.6, 130.7 (q, *J* = 32.5 Hz), 130.2, 129.0, 128.8, 127.8, 126.7, 124.6 (q, *J* = 3.5 Hz), 123.5 (q, *J* = 270.8 Hz), 35.4, 20.5, 13.3. ^19^F NMR (565 MHz, CDCl_3_) δ −63.10. HRMS (ESI-TOF) (*m*/*z*): Calcd for C_24_H_21_ClF_3_NNaO_4_S_2_^+^, ([M + Na]^+^), 566.0445, found 566.0453.

(*E*)-2-chloro-1-(4-(trifluoromethoxy)phenyl)pent-1-en-1-yl *N*-phenylsulfonylbenzenesulfonimidate **5n**

**1-6** (0.2 mmol, 45.6 mg) and **2b** (0.4 mmol, 132.4 mg) were employed. **5n** (PE/EtOAc = 10:1, Rf = 0.35) was purified by column chromatography on silica gel (PE/EtOAc = 30:1), white solid (50.2 mg, 45%), crystallization in CDCl_3_, mp. 86–87 °C. NMR spectroscopy: ^1^H NMR (600 MHz, CDCl_3_) δ 7.97 (d, *J* = 7.2 Hz, 2H), 7.60 (d, *J* = 7.8 Hz, 2H), 7.55 (t, *J* = 7.2 Hz, 1H), 7.49–7.46 (m, 3H), 7.28–7.25 (m, 2H), 7.22–7.17 (m, 2H), 6.88 (d, *J* = 7.8 Hz, 2H), 2.82–2.71 (m, 2H), 1.73–1.62 (m, 2H), 1.00 (t, *J* = 7.2 Hz, 3H). ^13^C NMR {^1^H} (125 MHz, CDCl_3_) δ 149.1, 142.7, 140.7, 136.0, 135.0, 134.5, 132.6, 131.5, 130.0, 128.9, 128.7, 127.9, 126.7, 120.2 (q, *J* = 256.6 Hz), 120.1, 35.4, 20.5, 13.2. ^19^F NMR (565 MHz, CDCl_3_) δ −57.65. HRMS (ESI-TOF) (*m*/*z*): Calcd for C_24_H_21_ClF_3_NNaO_5_S_2_^+^, ([M + Na]^+^), 582.0394, found 582.0403.

(*E*)-1-(3-bromophenyl)-2-chloropent-1-en-1-yl *N*-phenylsulfonylbenzenesulfonimidate **5o**

**1-12** (0.2 mmol, 44.4 mg) and **2b** (0.4 mmol, 132.4 mg) were employed. **5o** (PE/EtOAc = 10:1, Rf = 0.30) was purified by column chromatography on silica gel (PE/EtOAc = 30:1), colorless liquid (60.1 mg, 54%). NMR spectroscopy: ^1^H NMR (600 MHz, CDCl_3_) δ 7.97 (d, *J* = 7.2 Hz, 2H), 7.62 (dd, *J* = 8.4, 1.2 Hz, 2H), 7.55 (t, *J* = 7.2 Hz, 1H), 7.52–7.47 (m, 3H), 7.33–7.30 (m, 2H), 7.25–7.22 (m, 1H), 7.19 (d, *J* = 7.8 Hz, 1H), 7.17–7.16 (m, 1H), 6.97 (t, *J* = 7.8 Hz, 1H), 2.79–2.70 (m, 2H), 1.73–1.62 (m, 2H), 1.00 (t, *J* = 7.2 Hz, 3H). ^13^C NMR {^1^H} (150 MHz, CDCl_3_) δ 142.7, 140.6, 135.8, 135.2, 134.7, 133.2, 132.6, 132.4, 132.0, 129.2, 128.9, 128.8, 128.5, 127.8, 126.7, 121.7, 35.4, 20.5, 13.3. HRMS (ESI-TOF) (*m*/*z*): Calcd for C_23_H_21_BrClNNaO_4_S_2_^+^, ([M + Na]^+^), 575.9676, found 575.9684.

(*E*)-2-chloro-1-(2-fluorophenyl)pent-1-en-1-yl *N*-phenylsulfonylbenzenesulfonimidate **5p**

**1-13** (0.2 mmol, 32.4 mg) and **2b** (0.4 mmol, 132.4 mg) were employed. **5p** (PE/EtOAc = 10:1, Rf = 0.35) was purified by column chromatography on silica gel (PE/EtOAc = 30:1), white solid (45.1 mg, 46%), crystallization in CDCl_3_, mp. 83–84 °C. NMR spectroscopy: ^1^H NMR (600 MHz, CDCl_3_) δ 7.97 (d, *J* = 7.2 Hz, 2H), 7.64 (d, *J* = 7.8 Hz, 2H), 7.54 (t, *J* = 7.2 Hz, 1H), 7.47 (t, *J* = 7.2 Hz, 3H), 7.29–7.26 (m, 2H), 7.20 (t, *J* = 7.2 Hz, 1H), 7.17–7.13 (m, 1H), 6.93 (t, *J* = 7.2 Hz, 1H), 6.70 (t, *J* = 8.4 Hz, 1H), 2.78–2.69 (m, 2H), 1.72–1.61 (m, 2H), 0.99 (t, *J* = 7.2 Hz, 3H). ^13^C NMR {^1^H} (150 MHz, CDCl_3_) δ 159.4 (*J* = 251.1 Hz), 142.8, 136.9 (*J* = 24.5 Hz), 135.7, 134.3, 132.5, 132.1, 131.6 (*J* = 8.3 Hz), 130.9 (*J* = 5.1 Hz), 128.7 (*J* = 20.4 Hz), 127.8, 126.7, 123.5 (*J* = 3.5 Hz), 119.4 (*J* = 14.6 Hz), 115.4 (*J* = 21.2 Hz), 34.9, 20.5, 13.1. ^19^F NMR (565 MHz, CDCl_3_) δ −109.46 (bs). HRMS (ESI-TOF) (*m*/*z*): Calcd for C_23_H_21_ClFNNaO_4_S_2_^+^, ([M + Na]^+^), 516.0477, found 516.0494.

## 5. Conclusions

We achieved the robust trans-selective oxyiodination and oxychlorination of alkynes, employing *N*-iodosaccharin or *N*-chlorobenzenesulfonimide as the halogenation and oxygenation sources. This versatile approach tolerates many alkynes, including electron-rich and electron-deficient aryl-, bi-aryl, bi-alkyl, and terminal alkynes. The features of this transformation are as follows: easy operation, excellent functional group tolerance, broad substrate scope, and excellent trans-selectivity. Therefore, this method is an attractive alternative for synthesizing versatile halogenated enol esters and ethers. Employing highly electrophilic bifunctional reagents was key to achieving the general and practical halogenation of alkynes. To the best of our knowledge, this methodology represents the first oxyhalogenation of alkynes employing bifunctional N–X (halogen) reagents. Also, further applications in organic synthesis are ongoing in our lab.

## Figures and Tables

**Table 1:** Optimization of intermolecular oxyiodination of alkynes.

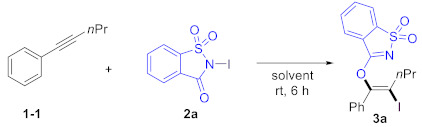			
Entry	2a	Solvent	Yield (%) *^a^*
1	1.0 equiv	DCM	68
2	1.2 equiv	DCM	86
3	1.5 equiv	DCM	85
4	1.2 equiv	CH_3_CN	13
5	1.2 equiv	DCE	80
6	1.2 equiv	Toluene	45
7	1.2 equiv	PhCF_3_	74
8	1.2 equiv	MeOH	n.d.
9 *^b^*	1.2 equiv	DCM	86

Reaction conditions: **1-1** (0.2 mmol), **2a**, Solvents (2 mL), 25 °C, under N_2_ atmosphere for 6 h. *^a^* Yield of the isolated product based on **1-1**. n.d. = not detected. *^b^* Reaction was carried out under air.

## Data Availability

The data presented in this study are available in the supplementary materials.

## Supplementary Materials

The following supporting information can be downloaded at https://www.mdpi.com/article/10.3390/molecules28217420/s1, X-ray crystallographic data of **3i** and **5j**, ^1^H, ^13^C, ^19^F NMR spectra for all new compounds are included in the Supplementary Materials.
