# Supplementary material for: Catalyst-Free Trans-Selective Oxyiodination and Oxychlorination of Alkynes Employing N–X (Halogen) Reagents
Source: Molecules. 2023 Nov 3;28(21):7420. doi: 10.3390/molecules28217420 (PMC10650761; doi:10.3390/molecules28217420)
Supplement: Supplementary file 1 [file molecules-28-07420-s001.zip › molecules-2651529-supplementary.pdf]

Catalyst-free Trans-selective Oxyiodination and Oxychlorina-tion of Alkynes Employing N–X (Halogen) Reagents

Jiaqiong Sun[1,†,*], Yunliang Guo[1,†], Jiuli Xia[2], Guangfan Zheng[2,*] and Qian Zhang[2,3]

Table of Contents

I. List for the Starting Materials **S2**

II. Optimization Conditions **S3**

III. X-ray Crystallographic Data **S4**

IV. NMR Spectra of New Compounds **S6**

I. List for the Starting Materials

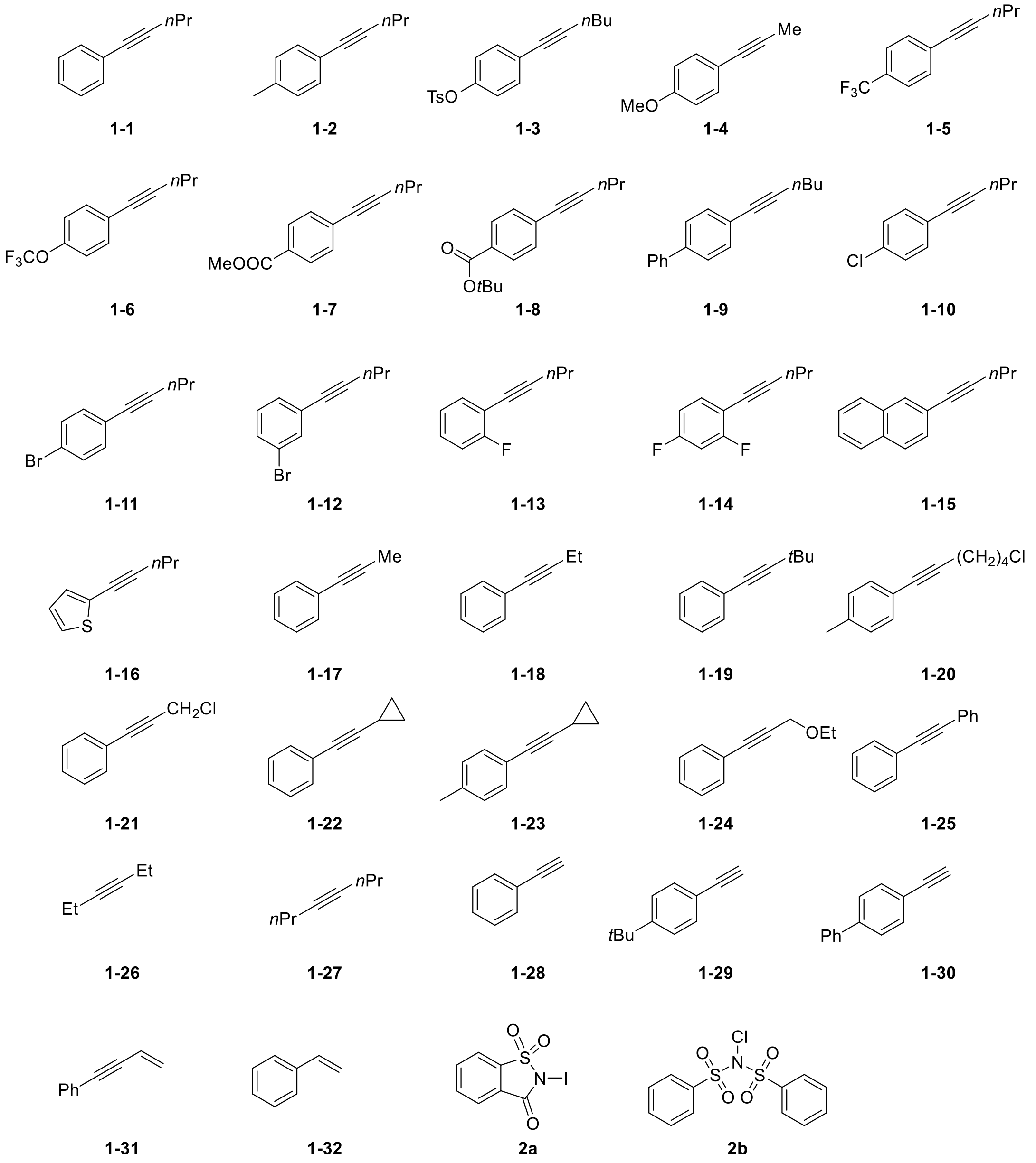

Scheme S1 List for the Starting Materials.

II. Optimization Conditions

Table S1. Optimization of intermolecular oxyiodination of alkynes.

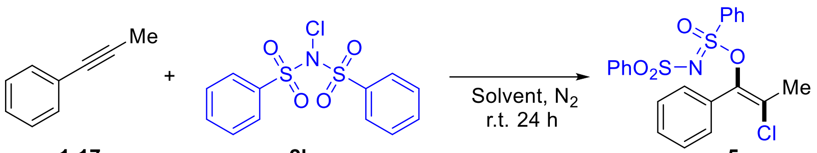

Entry	Solvent	T (°C)	Yield (%)[a]
1	DCM	25	66
2	DCE	25	70
3	$CHCl_3$	25	86
4	THF	25	16
5	Dioxane	25	22
6	Toluene	25	35
7	CH_3CN	25	29
8	$CHCl_3$	50	70
9	$CHCl_3$	70	33
10[b]	$CHCl_3$	25	77
11[c]	$CHCl_3$	25	85

Reaction conditions: **1-17** (0.2 mmol), **2b** (2.0 equiv), Solvents (2 mL), 25 °C, under N_2 atmosphere (g) for 24 h. [a]Yield of the isolated product based on **1-17**. [b] **2b** (1.5 equiv) was employed. [c] Employing Schlenk technical instead of glove box.

III. X-ray Crystallographic Data

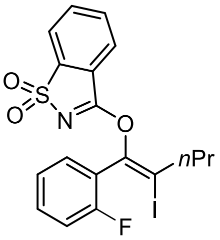

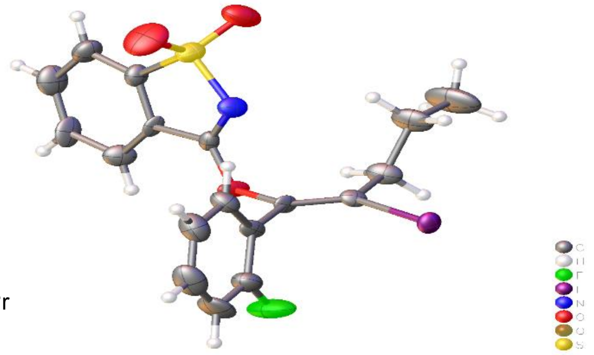

Crystal data and structure refinement for 3i

Identification code	CCDC_2304082
Empirical formula	$C_{18}H_{16}FINO_3S$
Formula weight	472.28
Temperature/K	293.01
Crystal system	triclinic
Space group	P-1
a/Å	9.532(3)
b/Å	9.899(4)
c/Å	10.483(4)
α/°	111.72(3)
β/°	90.99(2)
γ/°	95.57(3)
Volume/Å^3	913.1(6)
Z	2
ρ_{calc}g/cm^3	1.718
μ/mm^{-1}	15.088
F(000)	466.0
Crystal size/mm^3	0.21 × 0.21 × 0.15
Radiation	CuKα (λ = 1.54178)
2Θ range for data collection/°	9.096 to 127.34
Index ranges	-8 ≤ h ≤ 11, -11 ≤ k ≤ 11, -12 ≤ l ≤ 12
Reflections collected	5908
Independent reflections	2868 [R_{int} = 0.0636, R_{sigma} = 0.0773]
Data/restraints/parameters	2868/72/227
Goodness-of-fit on F^2	1.131
Final R indexes [I>=2σ (I)]	R_1 = 0.0996, wR_2 = 0.2432
Final R indexes [all data]	R_1 = 0.1033, wR_2 = 0.2494
Largest diff. peak/hole / e Å^{-3}	2.35/-4.73

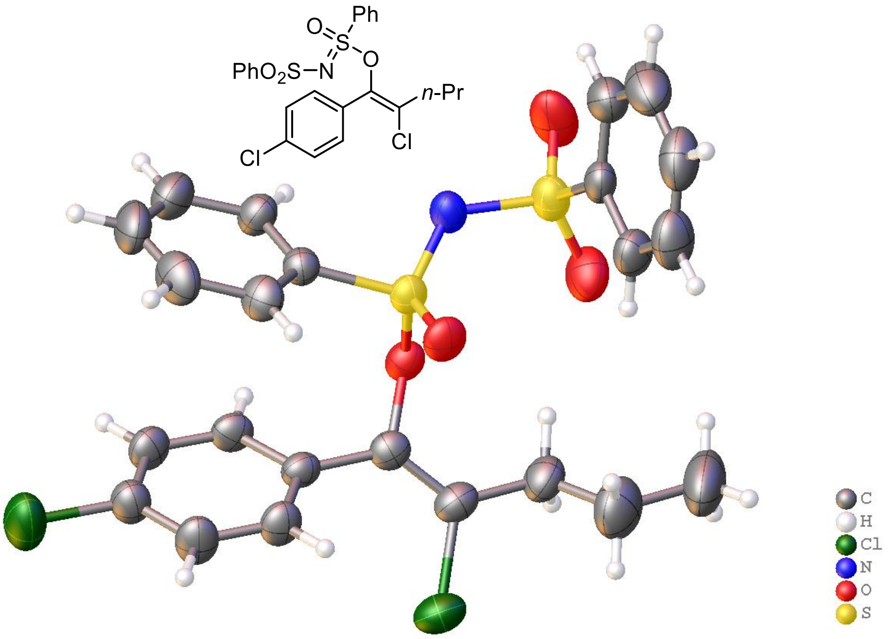

Crystal data and structure refinement for 5j

CCDC number	1829383
Empirical formula	C_{27} H_{20} Br N O_3 S
Formula weight	518.41
Temperature	293(2) K
Wavelength	0.71073 A
Crystal system, space group	Monoclinic, P21/C
Unit cell dimensions	a = 12.857 A alpha = 90 deg.<br>b = 15.123 A beta = 102.04 deg.<br>c = 12.073 A gamma = 90 deg.
Volume	2295.8 A^3
Z, Calculated density	4, 1.500 Mg/m^3
Reflections collected / unique	12707 / 4058 [R(int) = 0.0561]
F(000)	1056
Absorption correction	Semi-empirical from equivalents
Max. and min. transmission	0.7625 and 0.5626
Refinement method	Full-matrix least-squares on F^2
Data / restraints / parameters	4058 / 0 / 298
Goodness-of-fit on F^2	1.007
Final R indices [I>2sigma(I)]	R_1 = 0.0490, wR_2 = 0.1117
R indices (all data)	R_1 = 0.0805, wR_2 = 0.1268
Largest diff. peak and hole	0.540 and -0.288 e.A^-3
[a]$R_1 = \Sigma\|\|F_o\|-\|Fc\|\|/\Sigma\|Fo\|$;[b]$wR_2 = \Sigma[w(F_o^2-F_c^2)^2]/\Sigma[w(F_o^2)^2]^{1/2}$	

IV. NMR Spectra of New Compounds

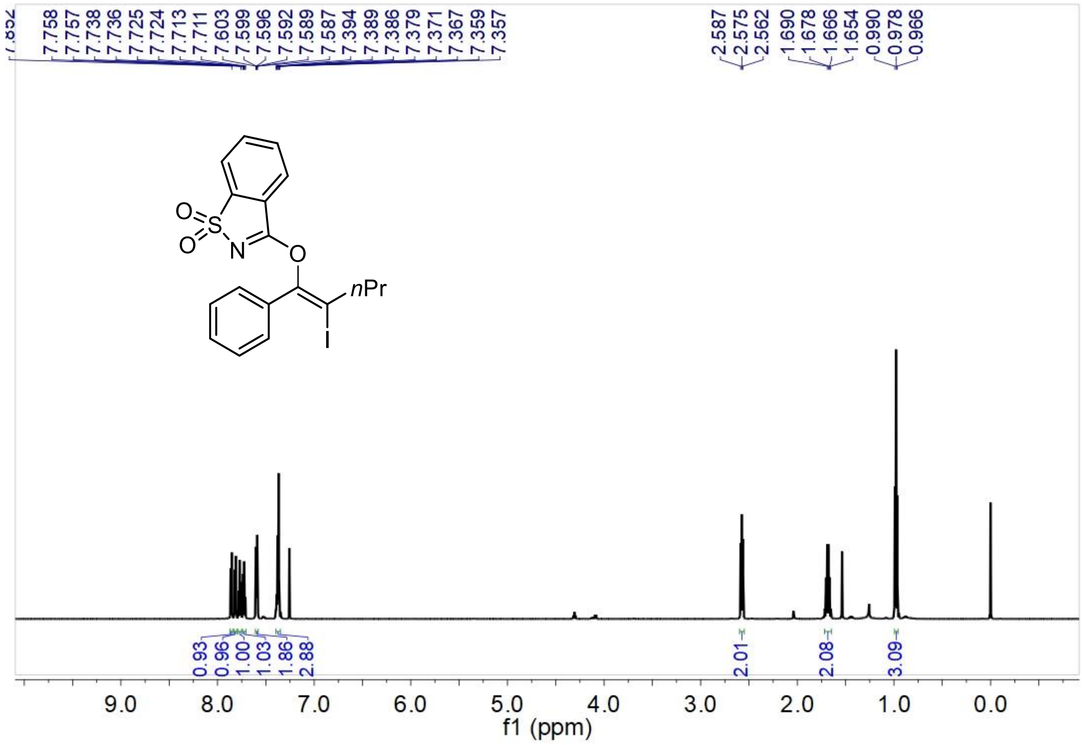

^{1}H NMR (600 MHz, $CDCl_3$) spectrum of 3a.

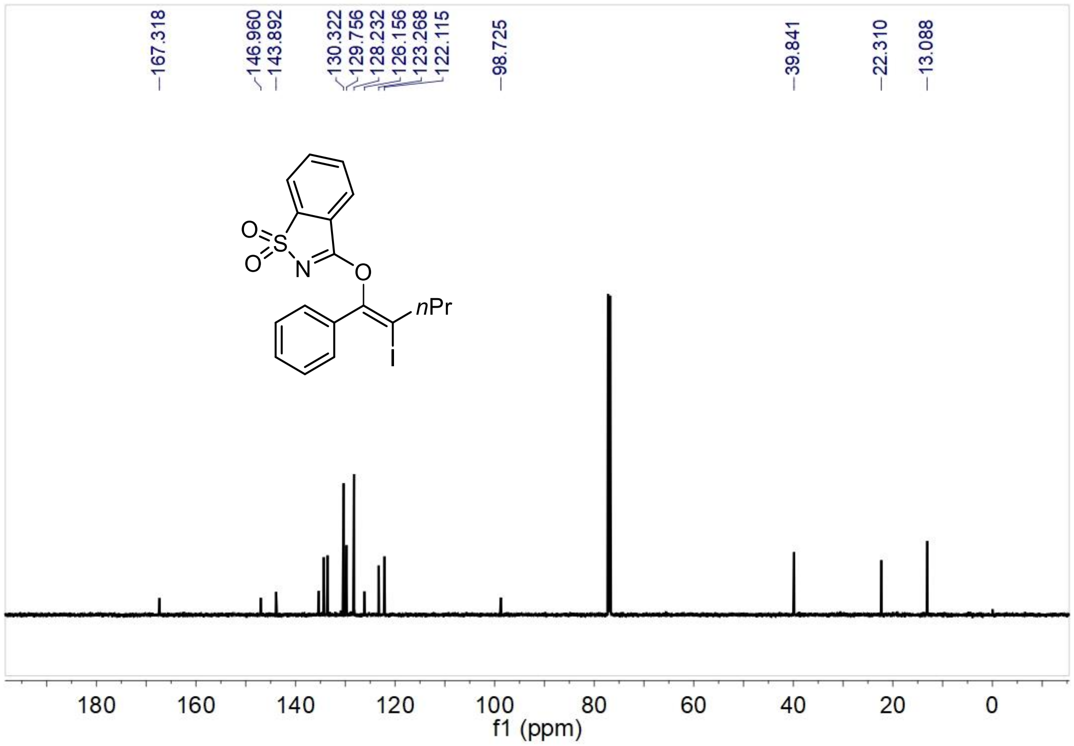

^{13}C NMR (150 MHz, $CDCl_3$) spectrum of 3a.

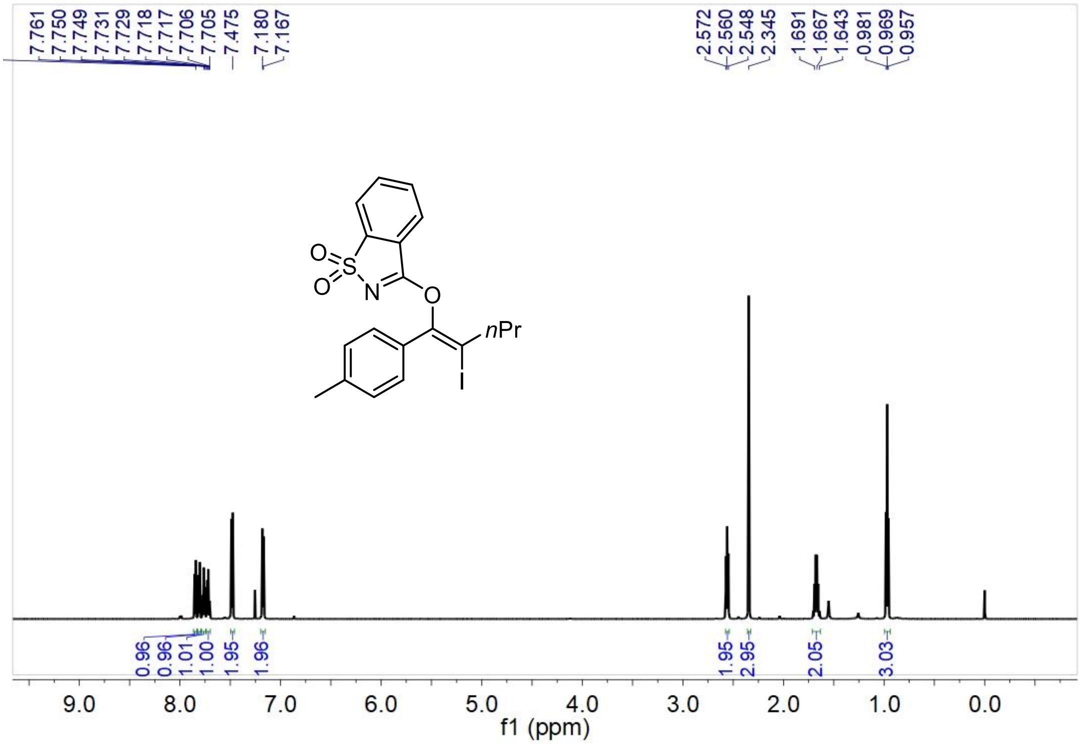

^{1}H NMR (600 MHz, CDCl3) spectrum of 3b.

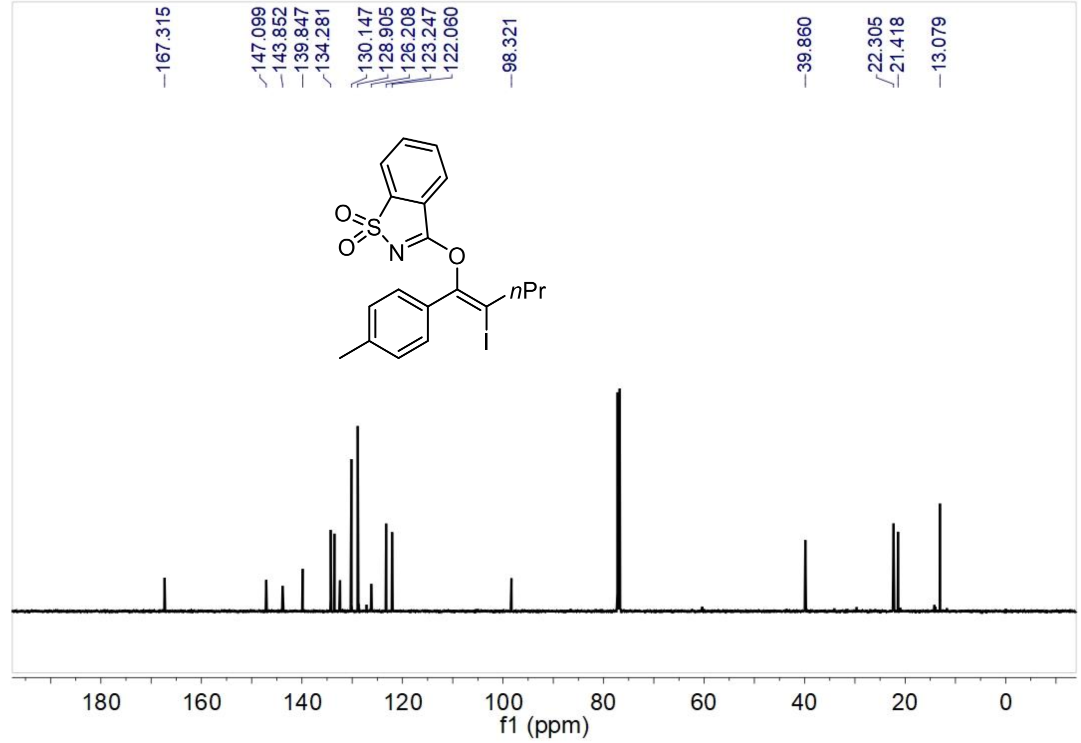

^{13}C NMR (150 MHz, CDCl3) spectrum of 3b.

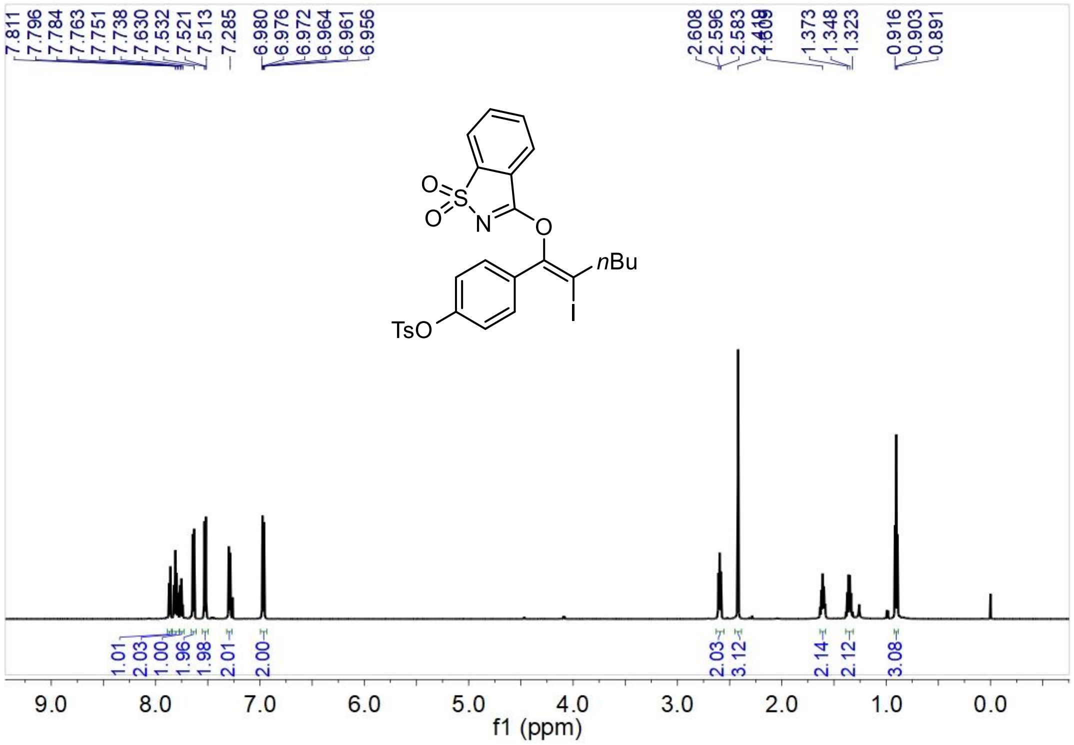

^{1}H NMR (600 MHz, CDCl3) spectrum of 3c.

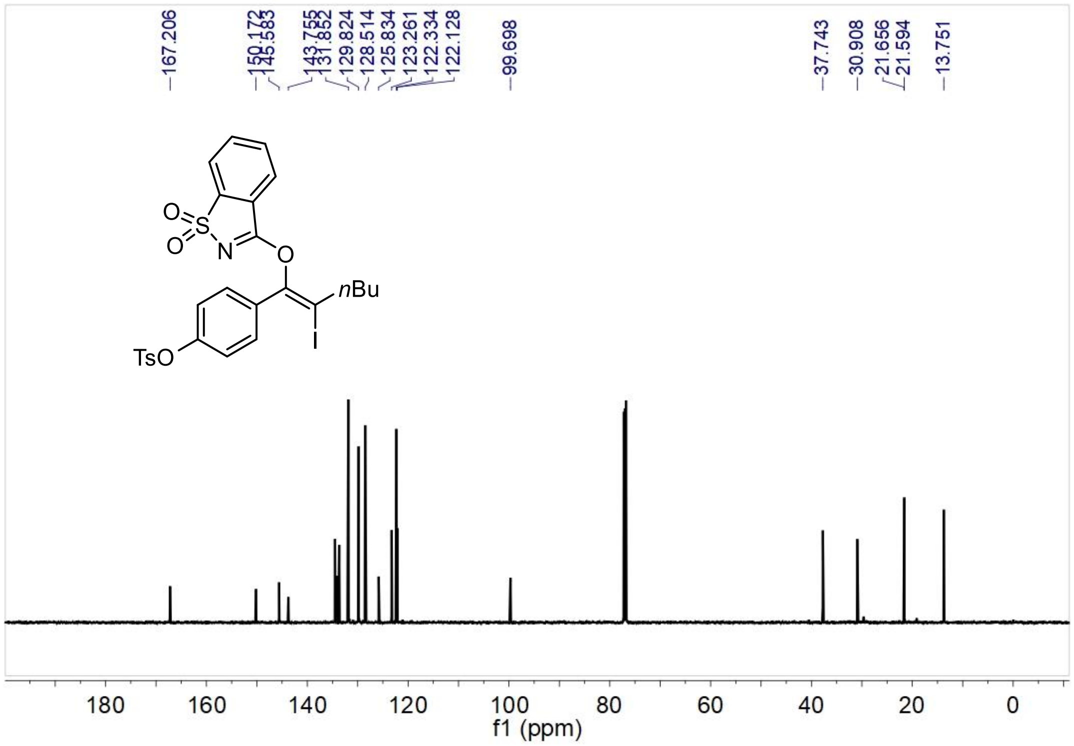

^{13}C NMR (150 MHz, CDCl3) spectrum of 3c.

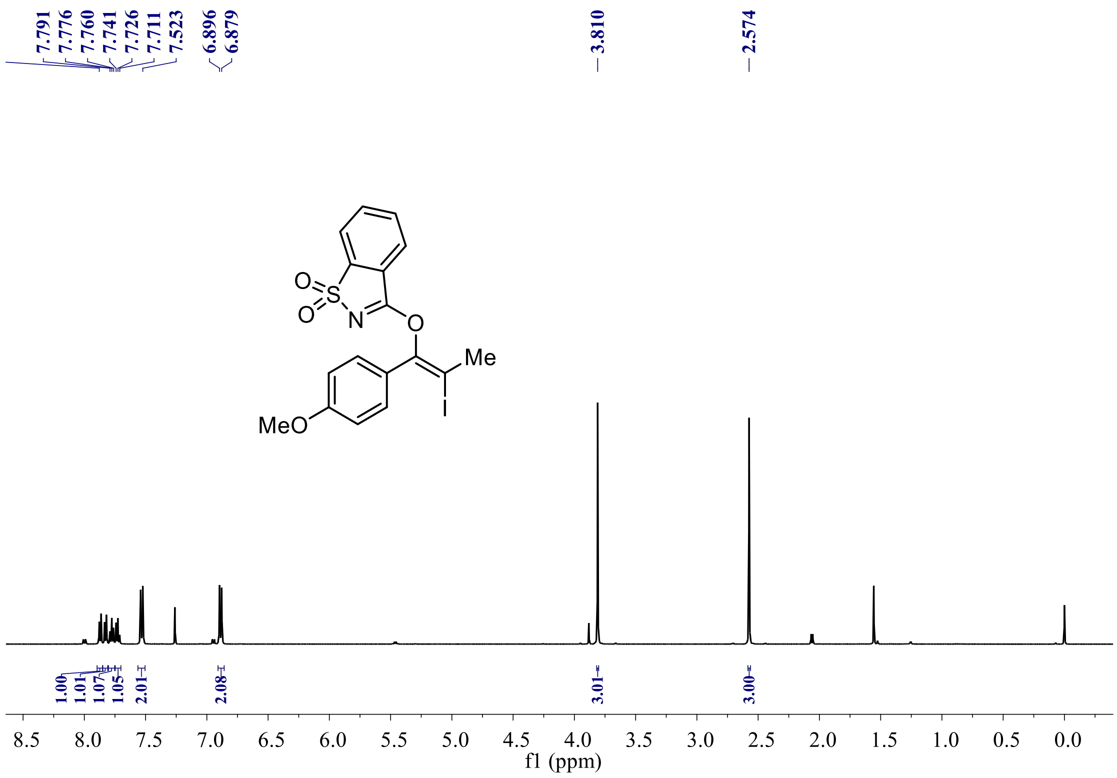

^{1}H NMR (500 MHz, CDCl3) spectrum of 3d.

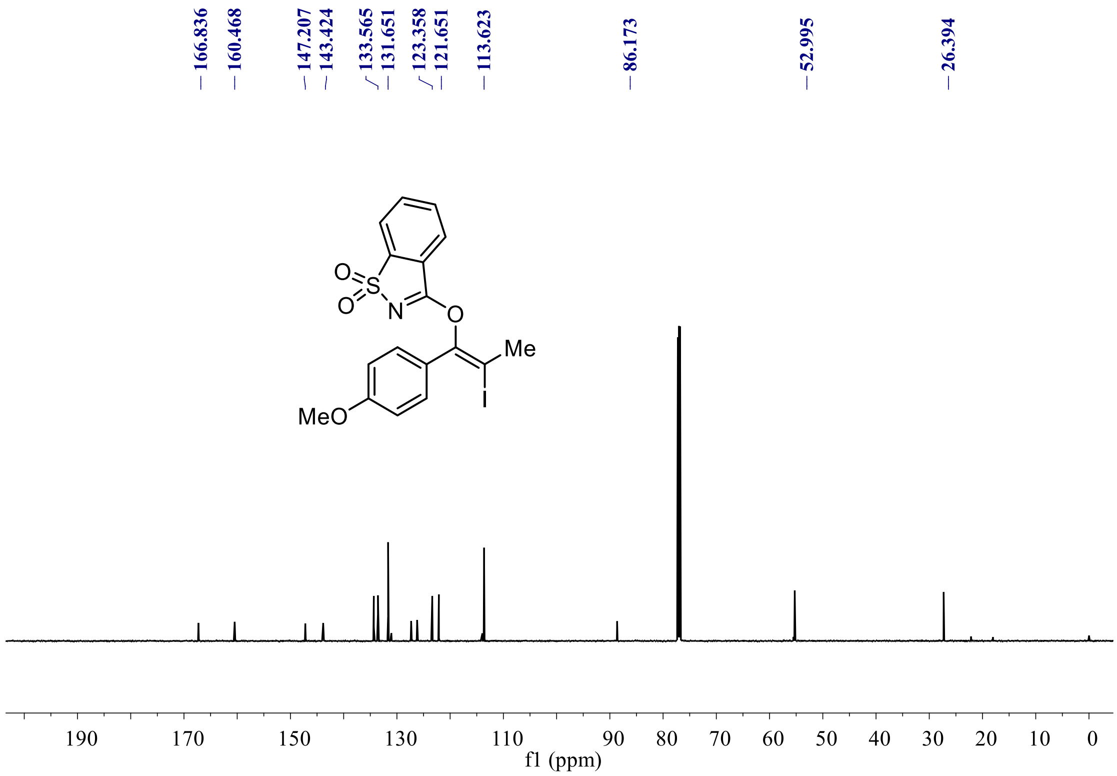

^{13}C NMR (150 MHz, CDCl3) spectrum of 3d.

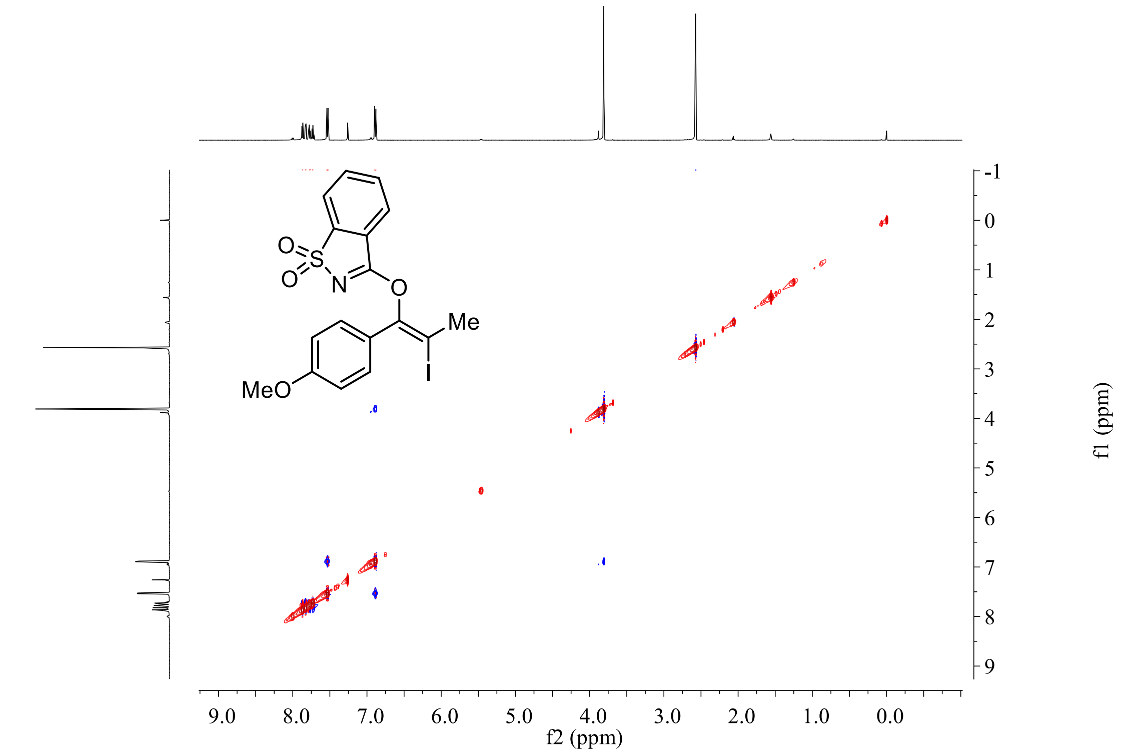

^{1}H-^{1}H NOE spectrum of 3d.

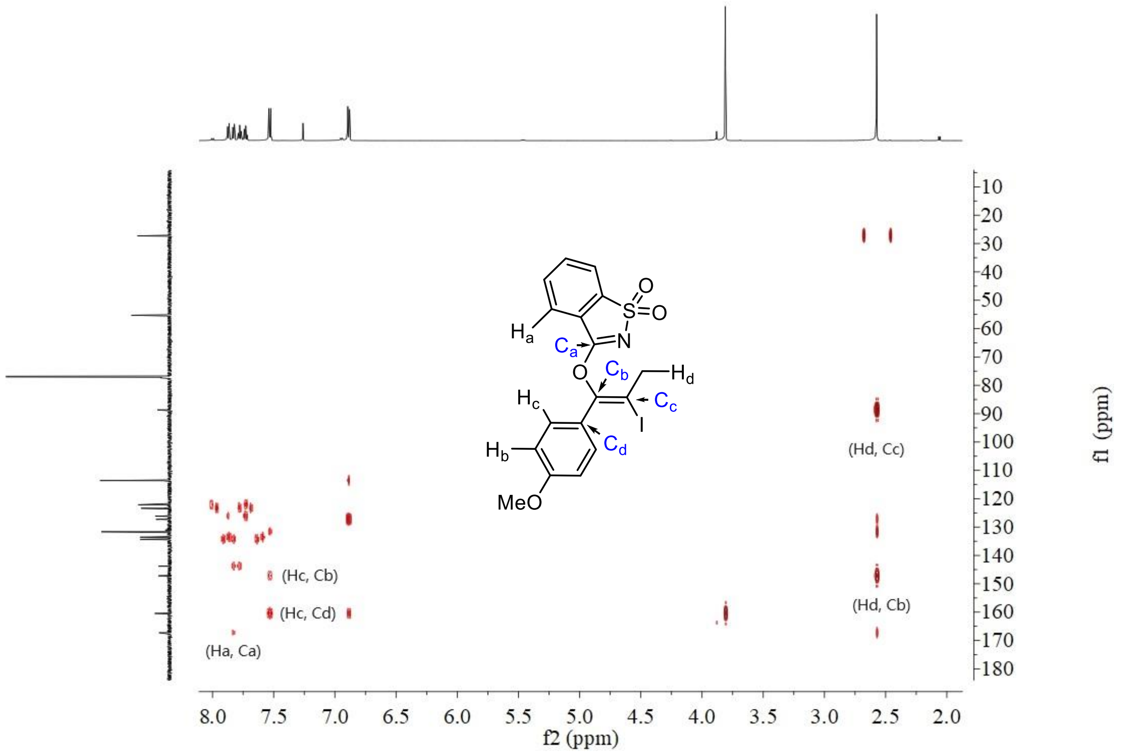

^{1}H-^{13}C HMQC spectrum of 3d.

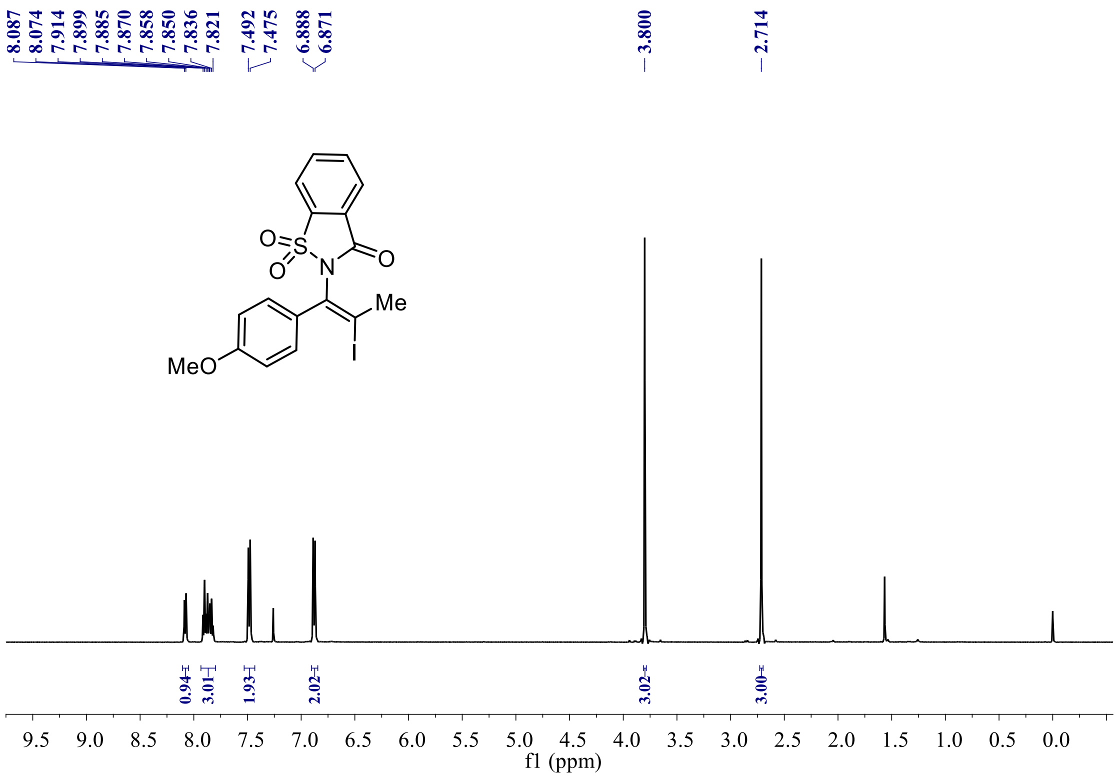

^{1}H NMR (500 MHz, CDCl$_3$) spectrum of 3d'.

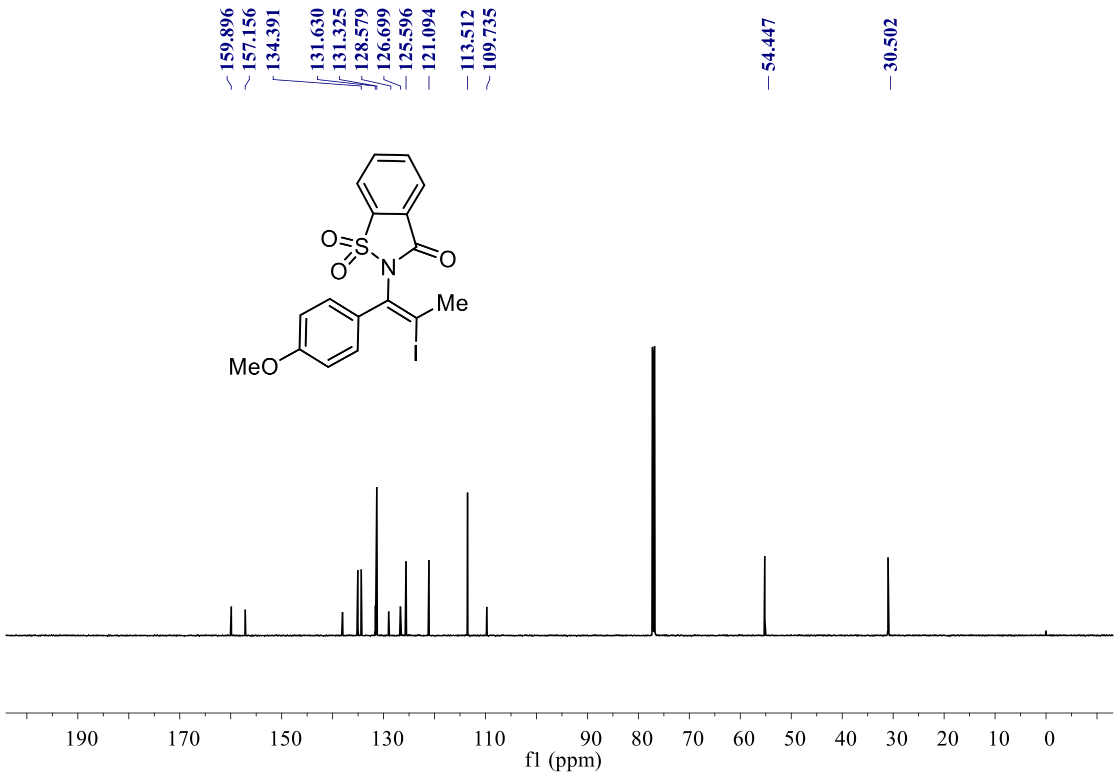

^{13}C NMR (150 MHz, CDCl$_3$) spectrum of 3d'.

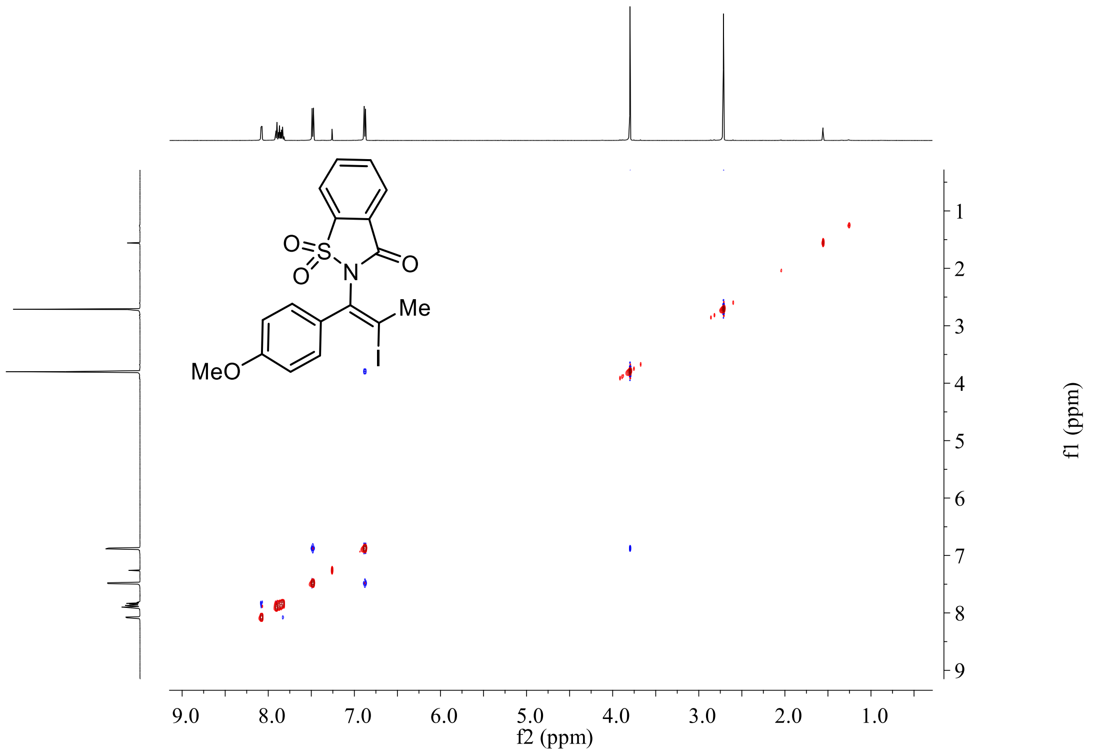

^{1}H-^{1}H NOESY spectrum of 3d'.

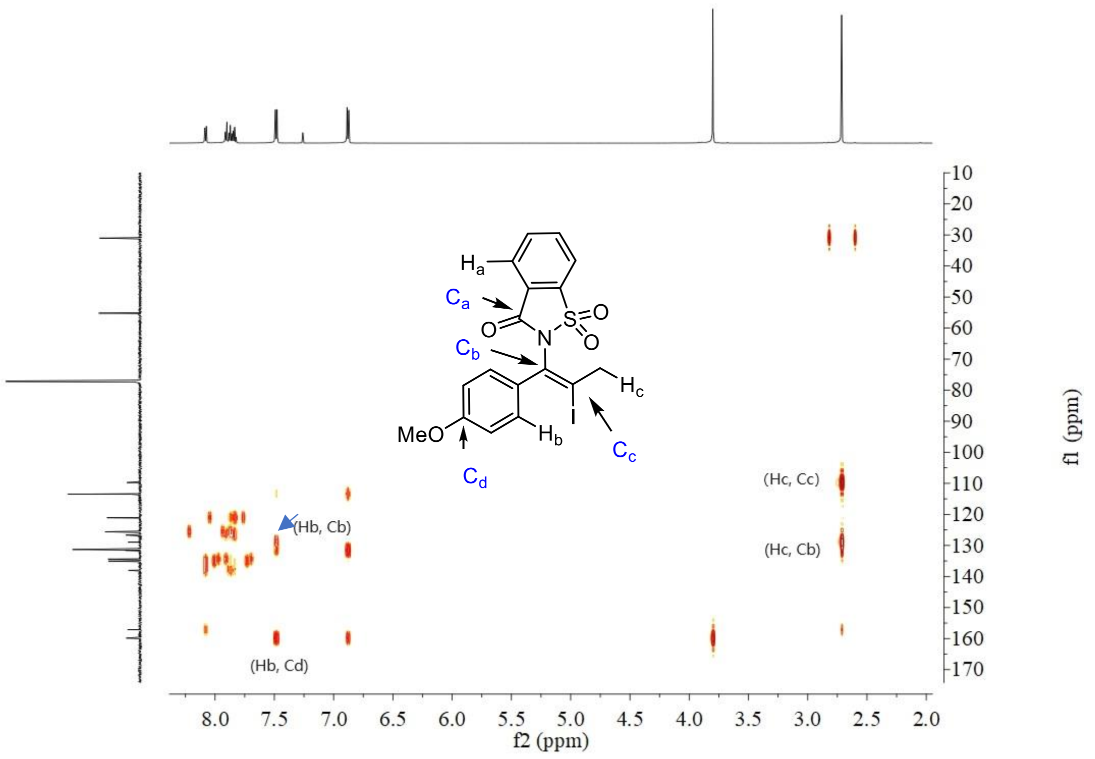

^{1}H-^{13}C HMQC spectrum of 3d'.

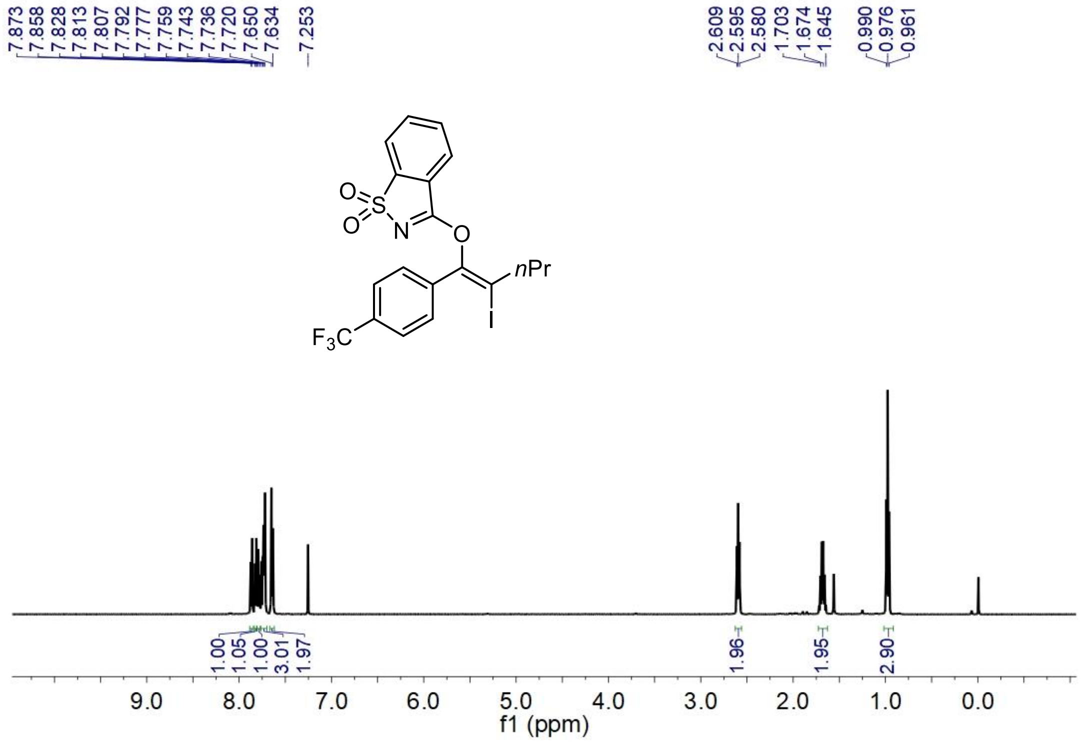

^{1}H NMR (500 MHz, CDCl3) spectrum of 3e.

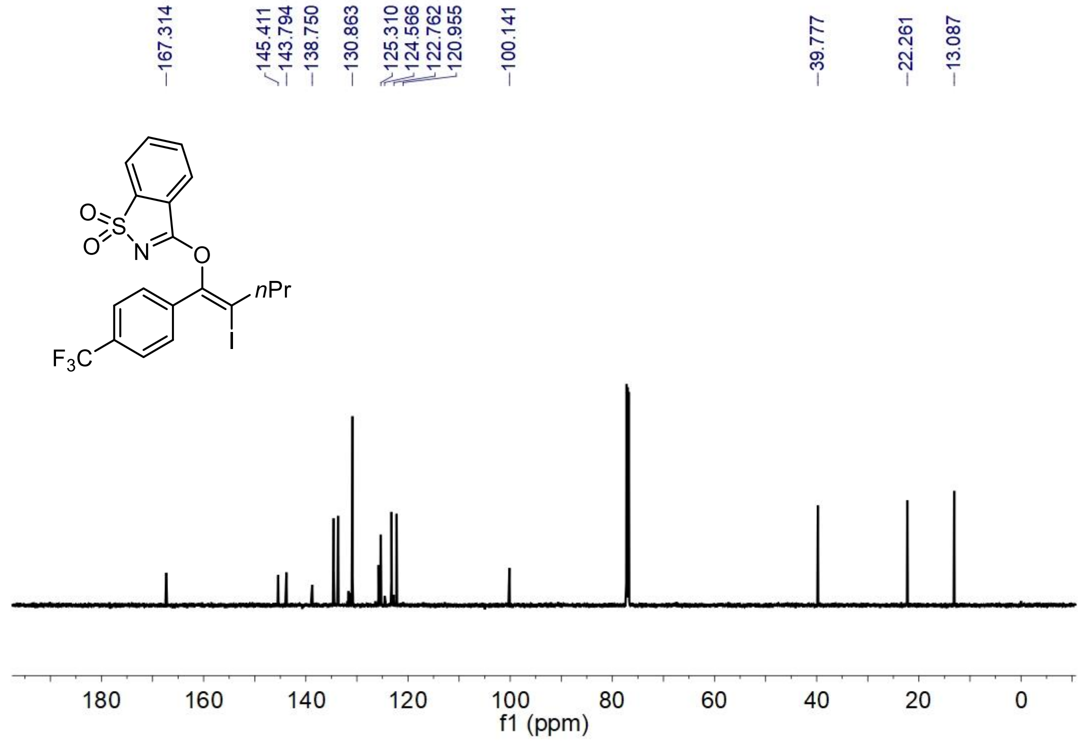

^{13}C NMR (150 MHz, CDCl3) spectrum of 3e.

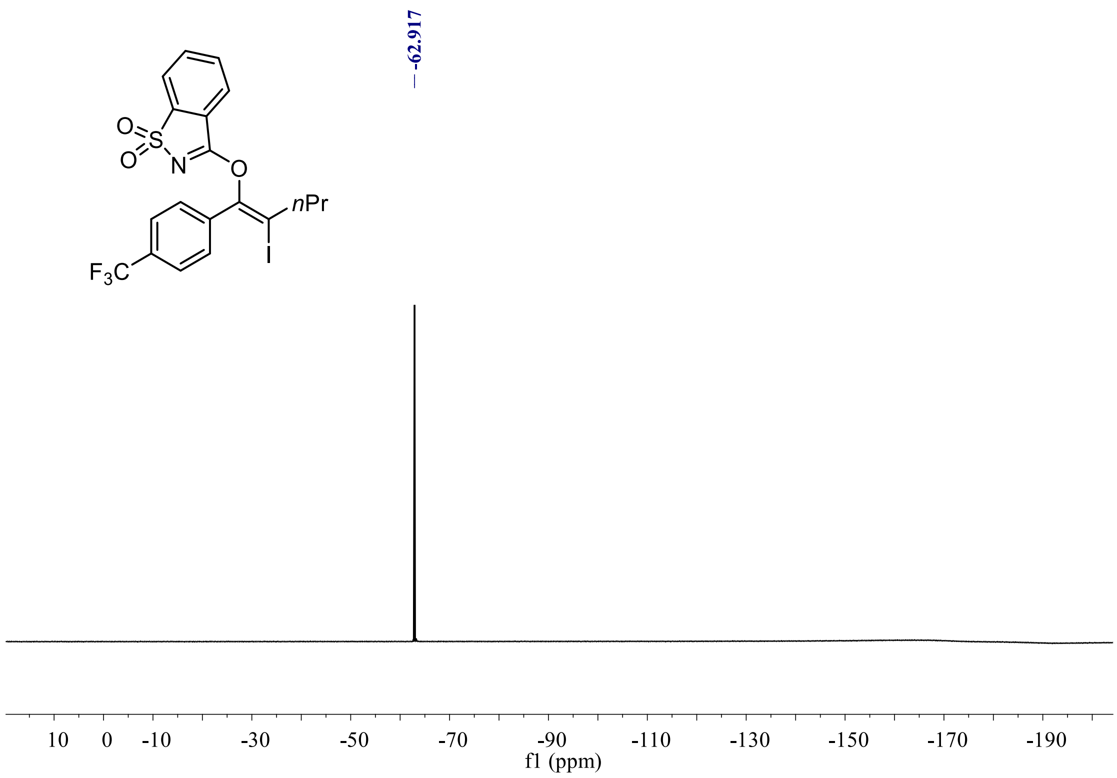

^{19}F NMR (565 MHz, CDCl$_3$) spectrum of 3e.

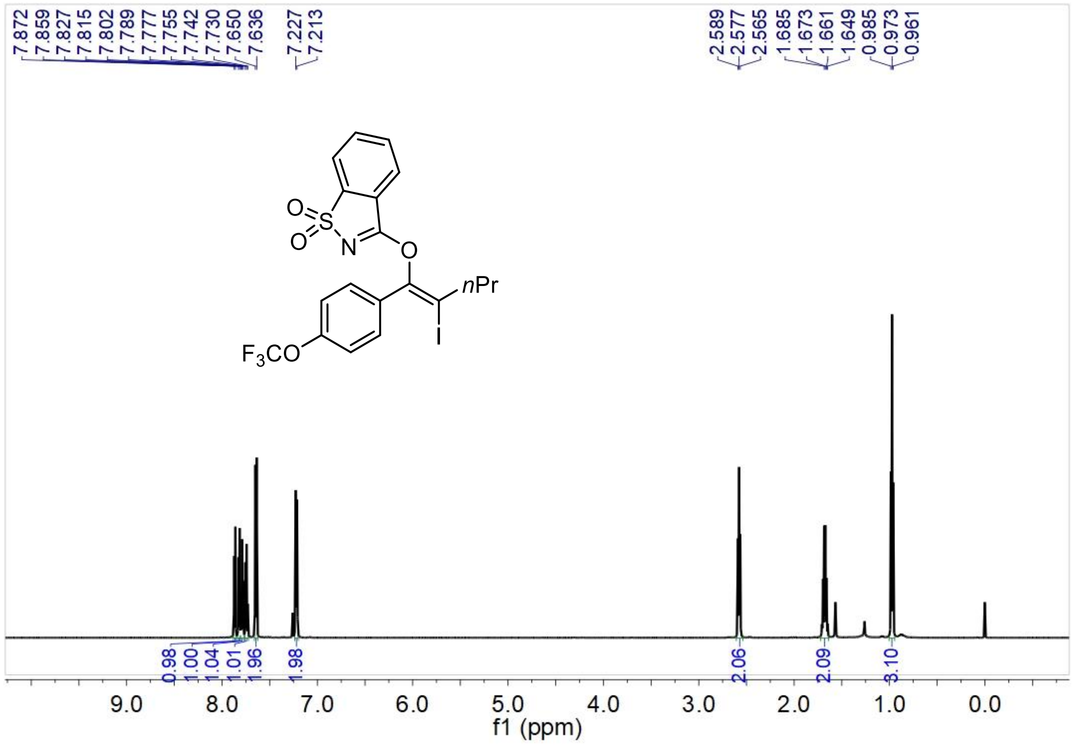

^{1}H NMR (600 MHz, $CDCl_3$) spectrum of 3f.

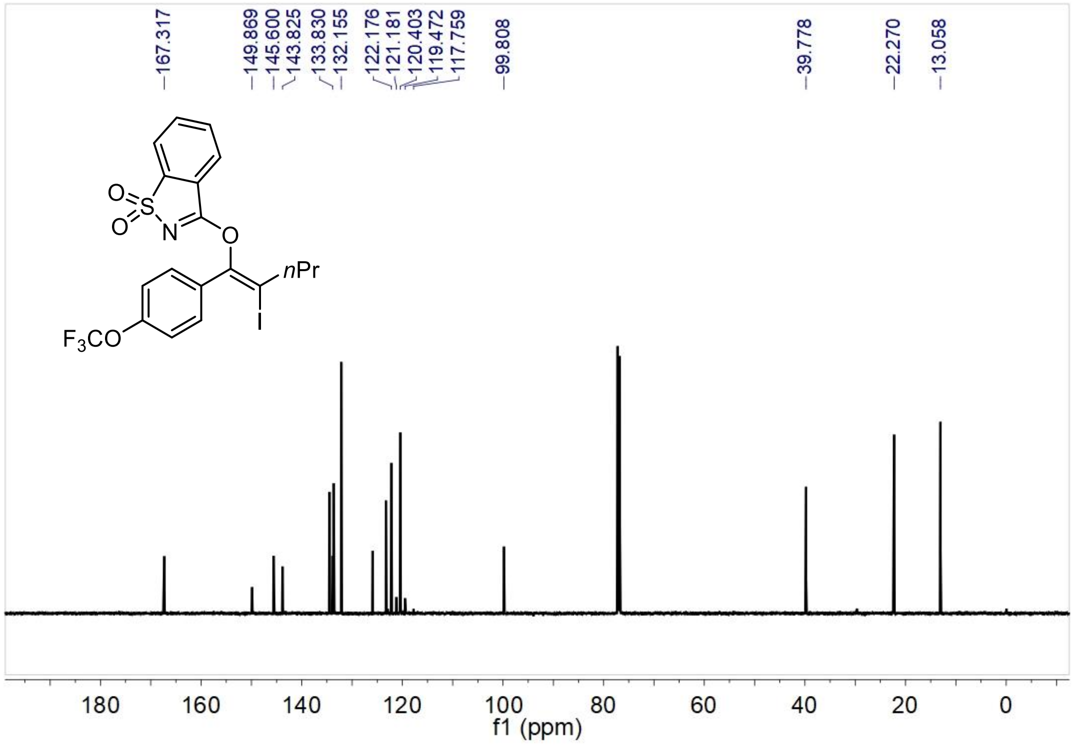

^{13}C NMR (150 MHz, $CDCl_3$) spectrum of 3f.

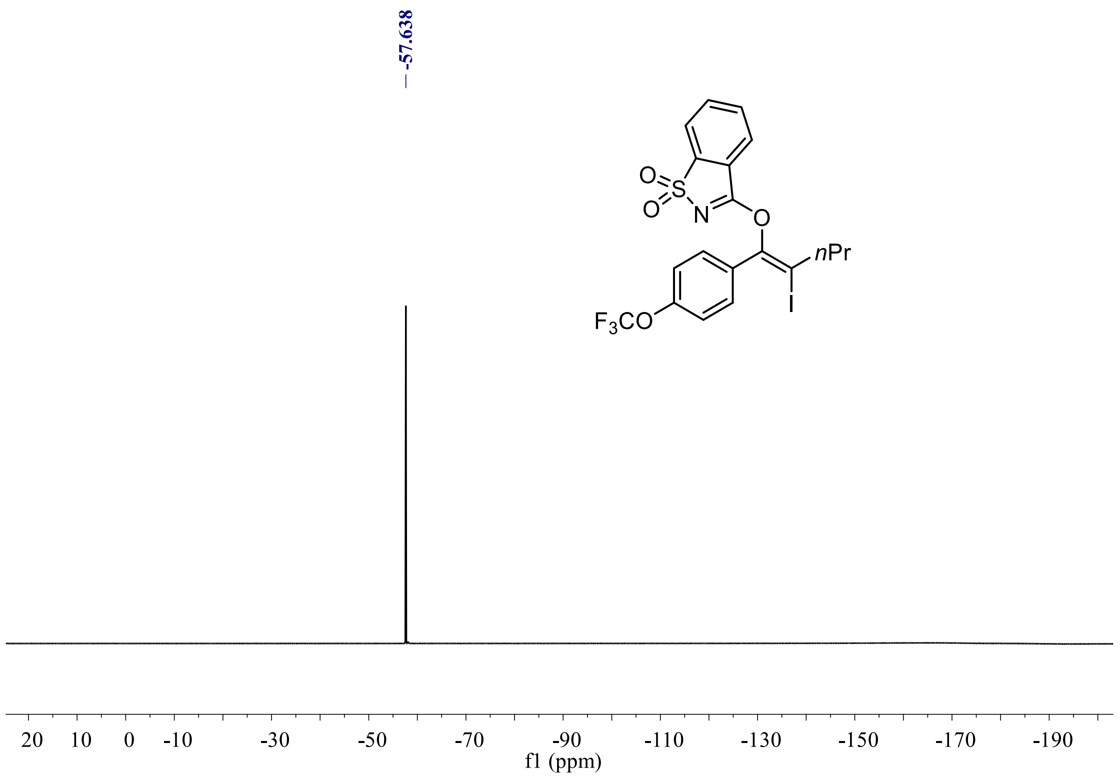

^{19}F NMR (565 MHz, CDCl3) spectrum of 3f.

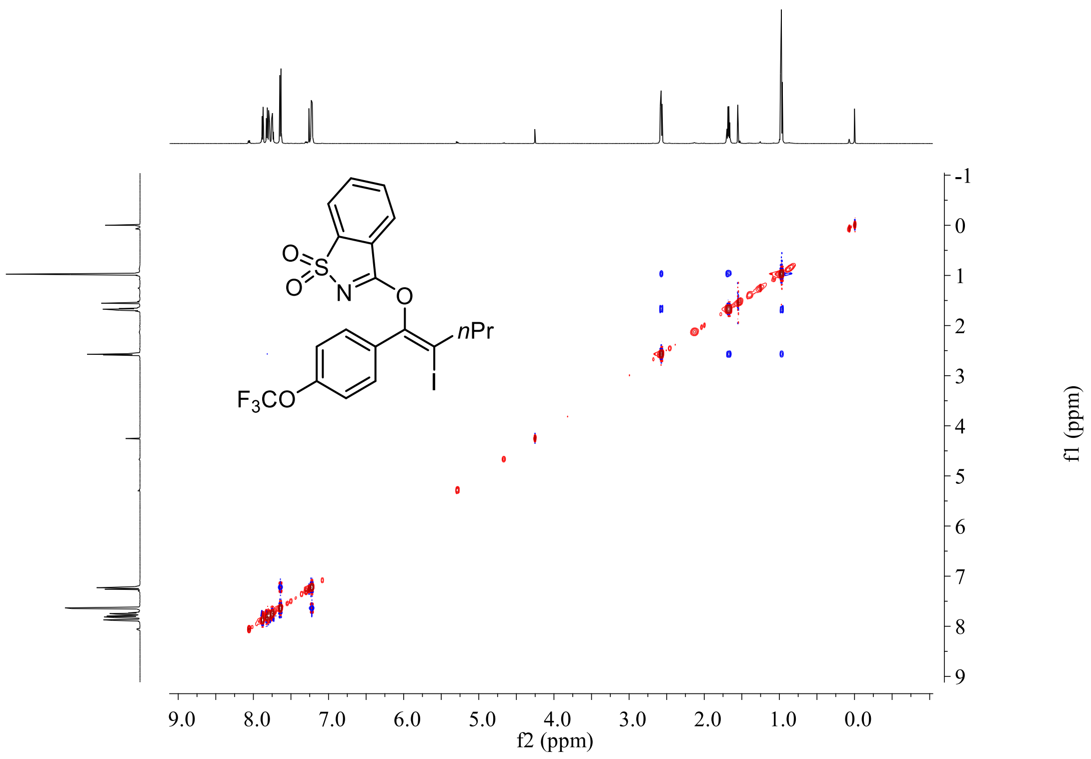

^{1}H-^{1}H NOESY spectrum of 3f.

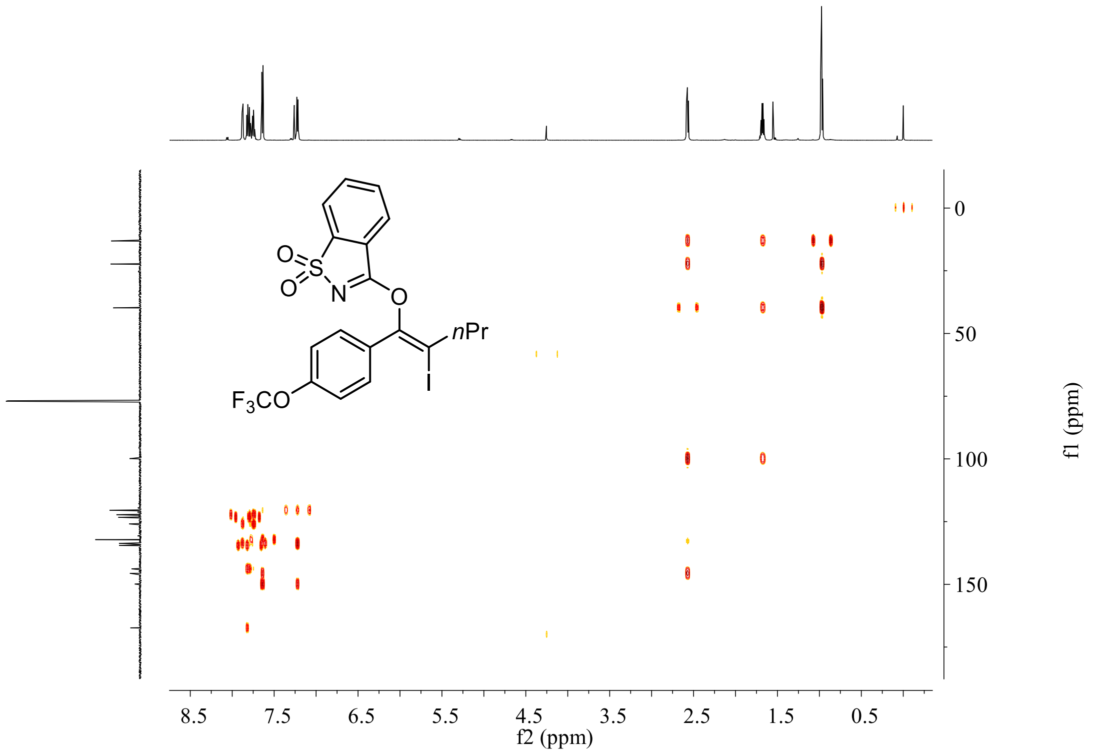

^{1}H-^{13}C HMQC spectrum of 3f.

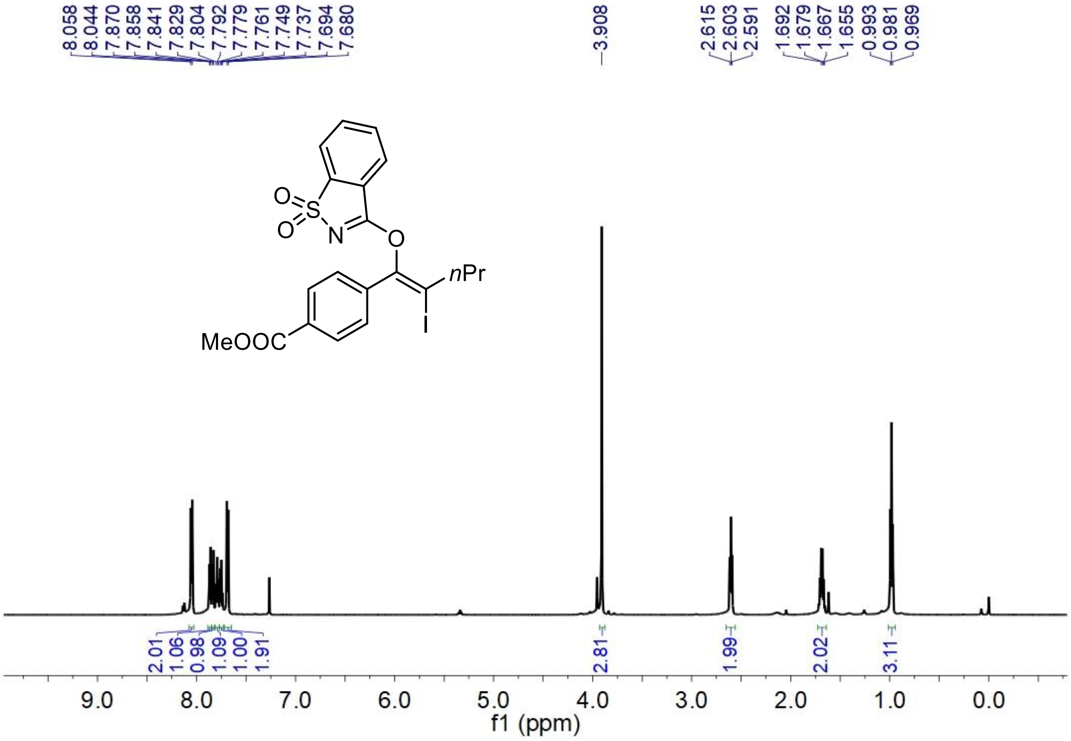

^{1}H NMR (600 MHz, CDCl3) spectrum of 3g.

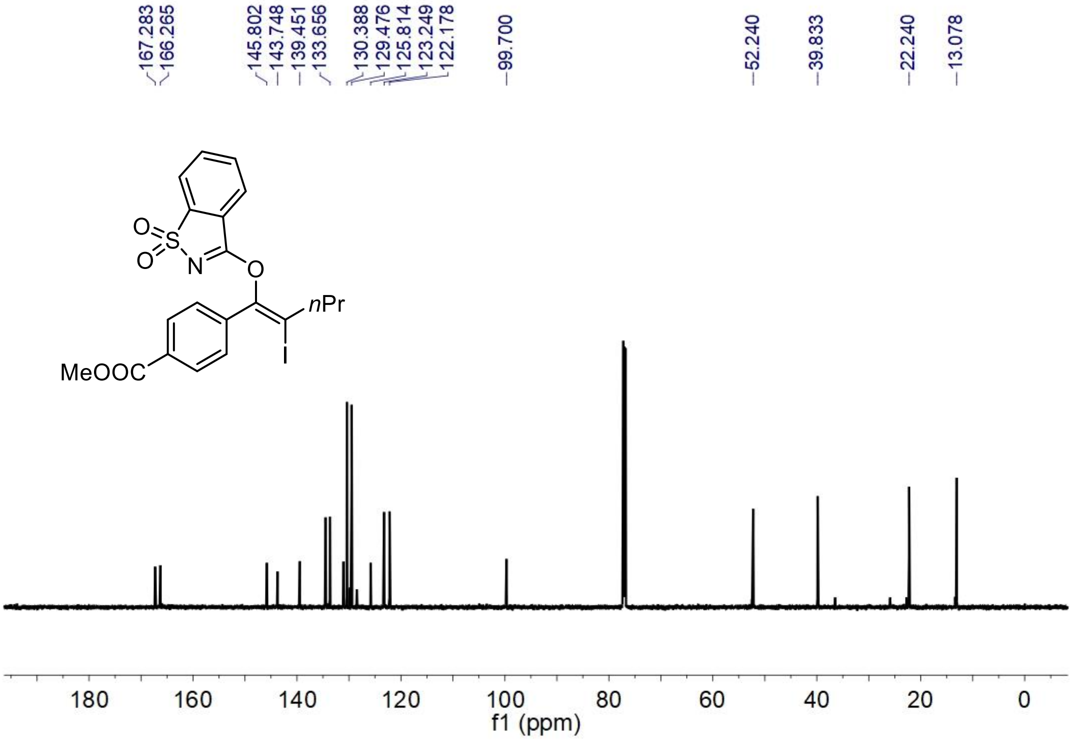

^{13}C NMR (150 MHz, CDCl3) spectrum of 3g.

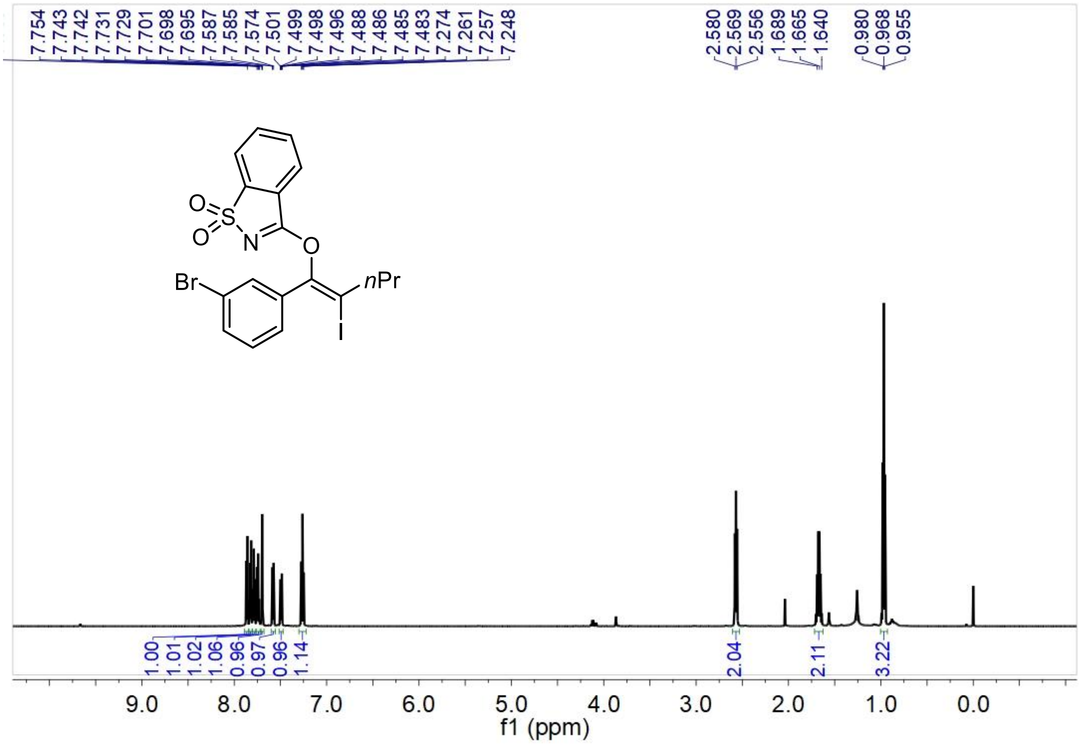

^{1}H NMR (600 MHz, $CDCl_3$) spectrum of 3h.

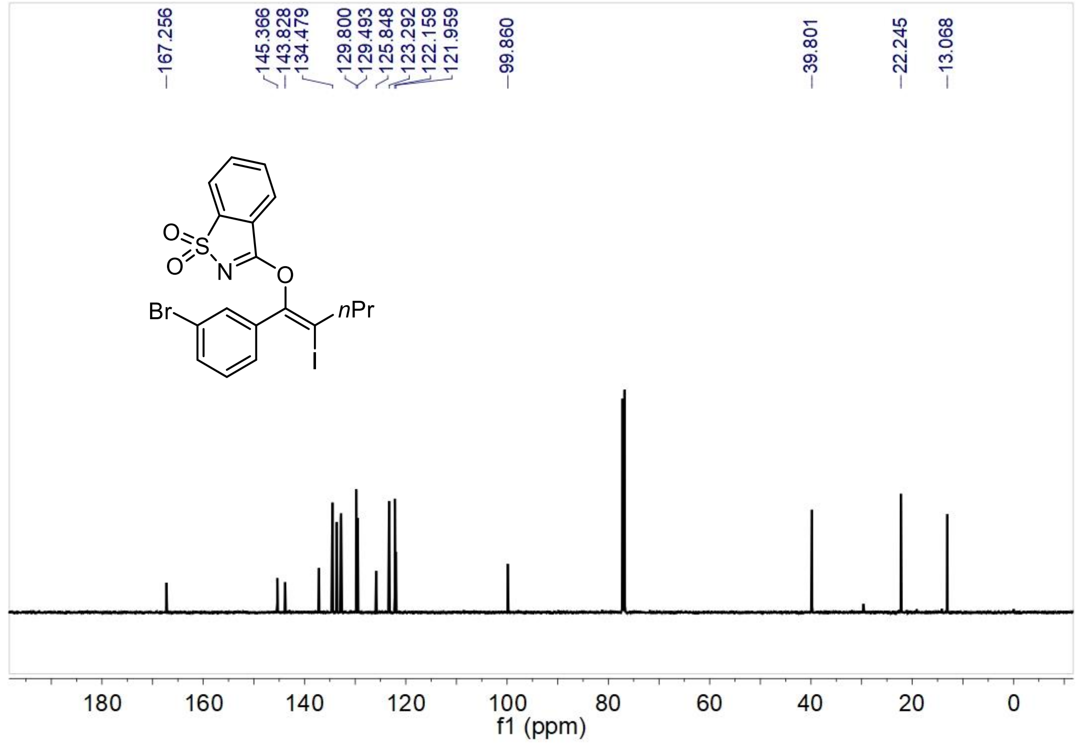

^{13}C NMR (150 MHz, $CDCl_3$) spectrum of 3h.

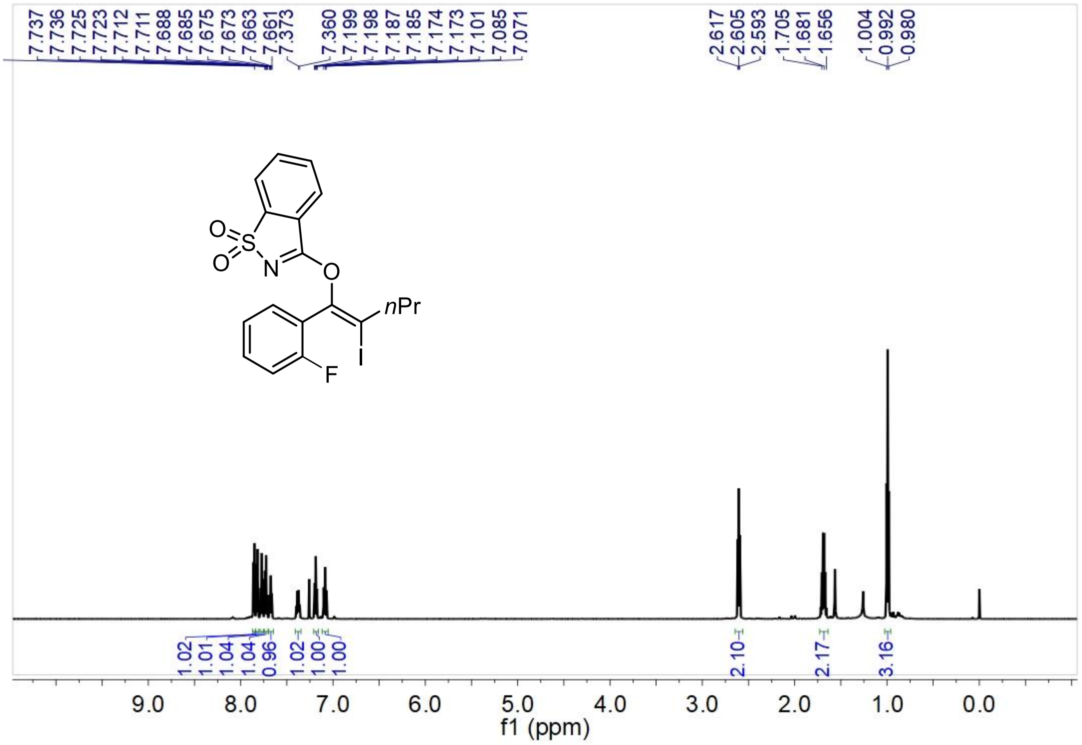

^{1}H NMR (600 MHz, $CDCl_3$) spectrum of 3i.

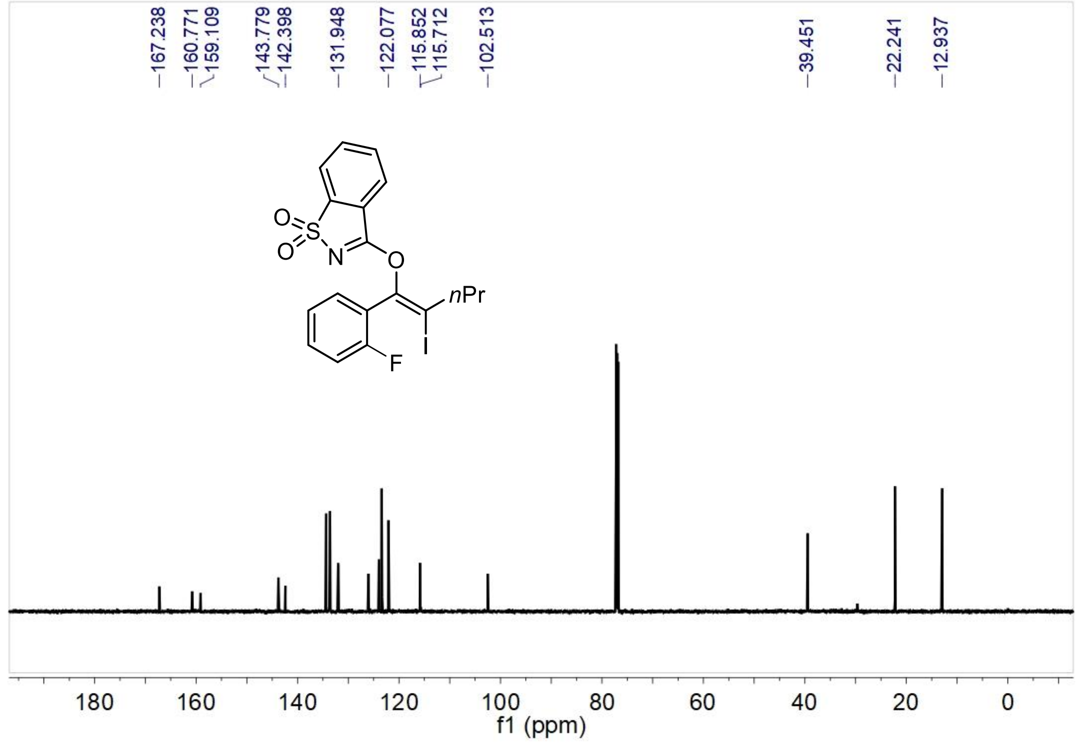

^{13}C NMR (150 MHz, $CDCl_3$) spectrum of 3i.

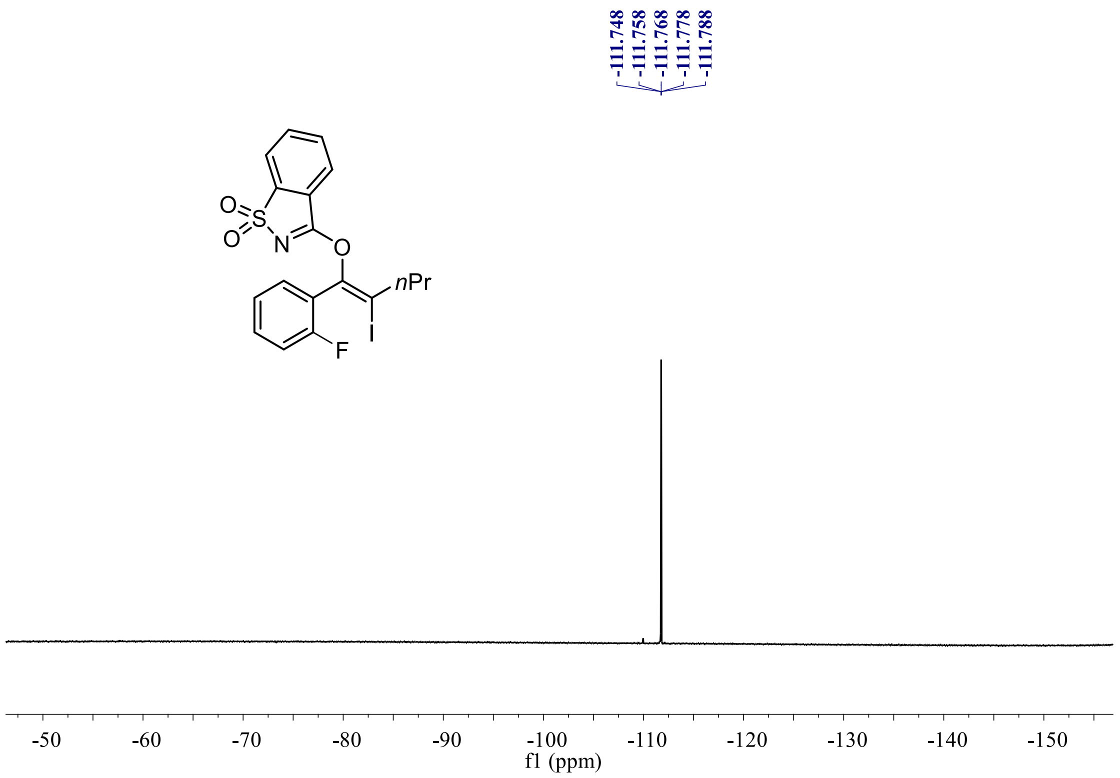

^{19}F NMR (565 MHz, $CDCl_3$) spectrum of 3i.

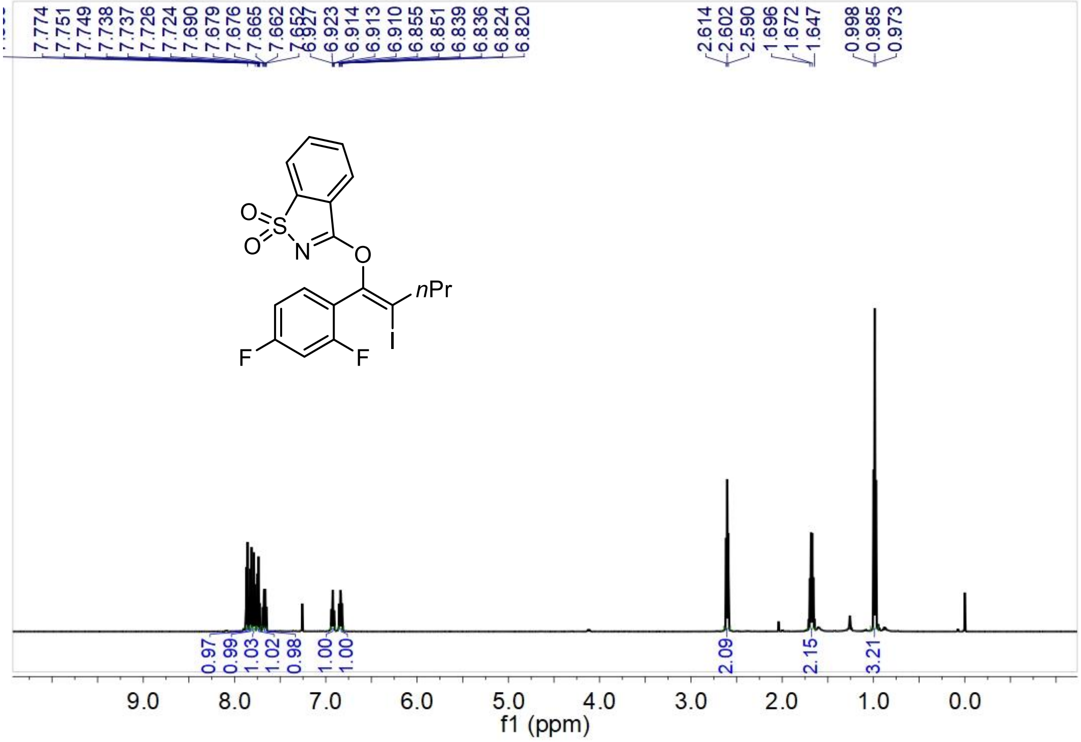

^{1}H NMR (600 MHz, CDCl3) spectrum of 3j.

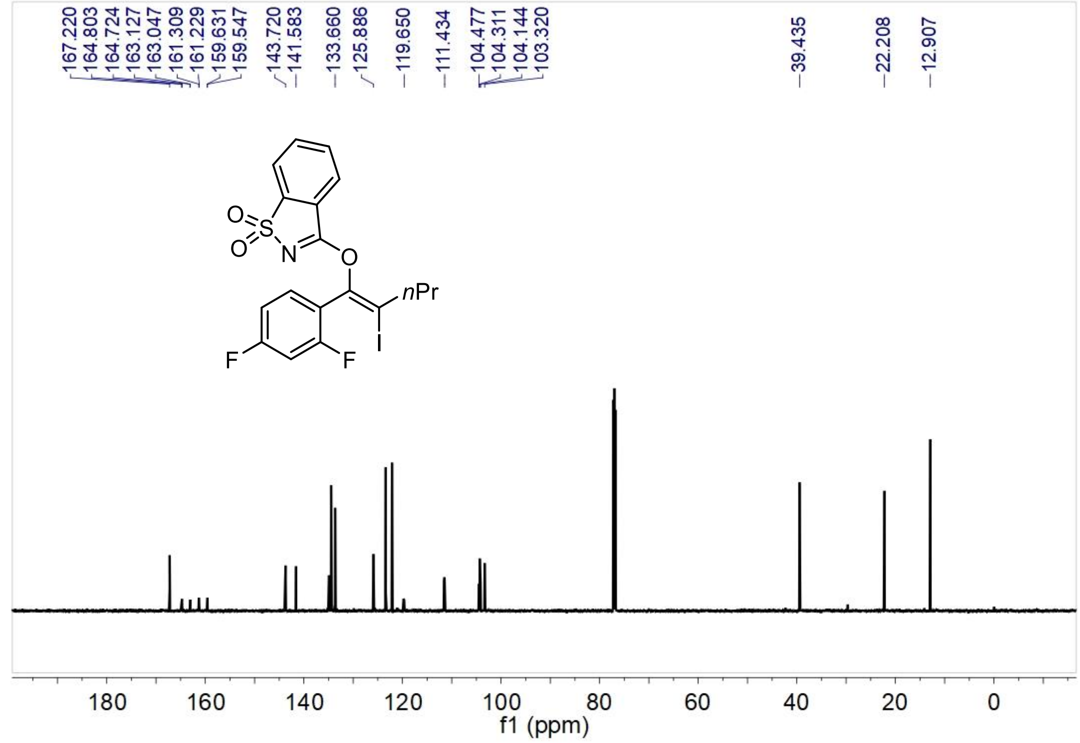

^{13}C NMR (150 MHz, CDCl3) spectrum of 3j.

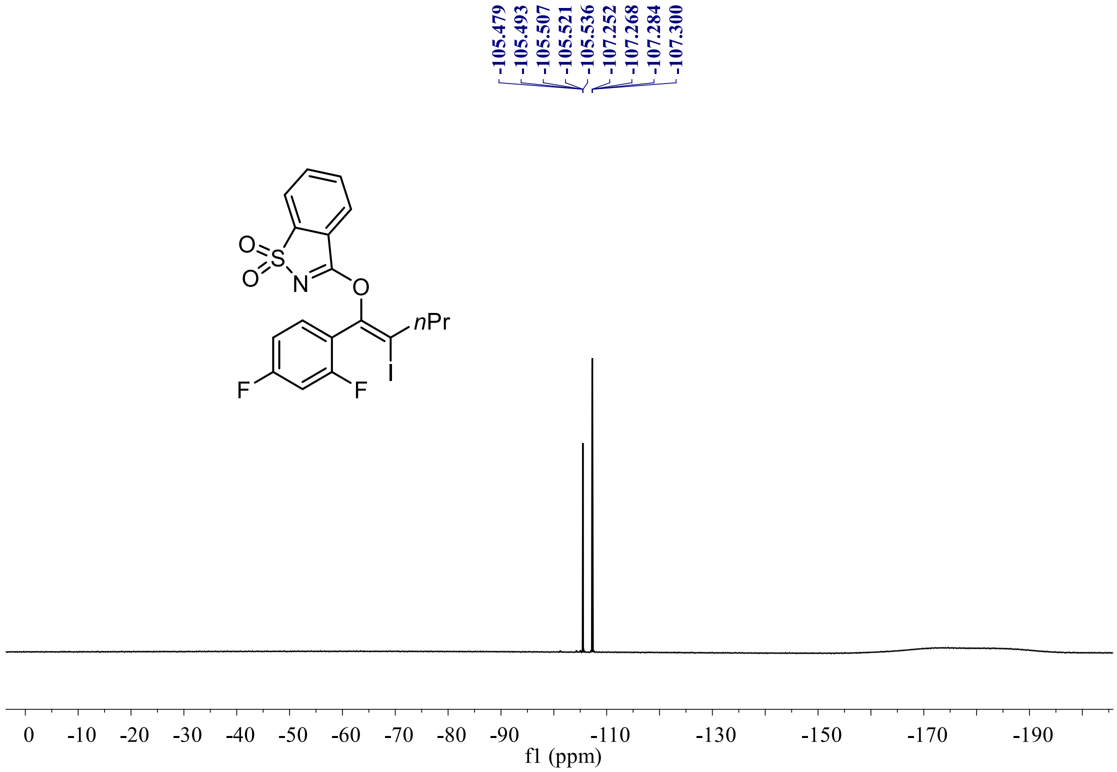

^{19}F NMR (565 MHz, $CDCl_3$) spectrum of 3j.

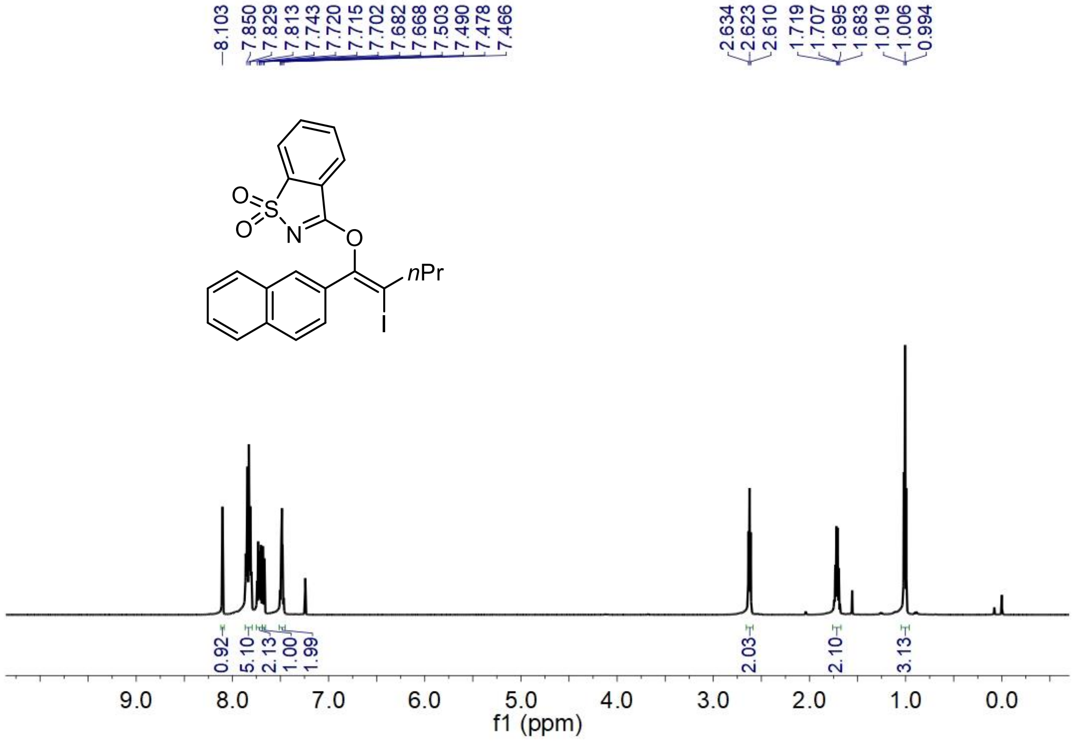

^{1}H NMR (600 MHz, $CDCl_3$) spectrum of 3k.

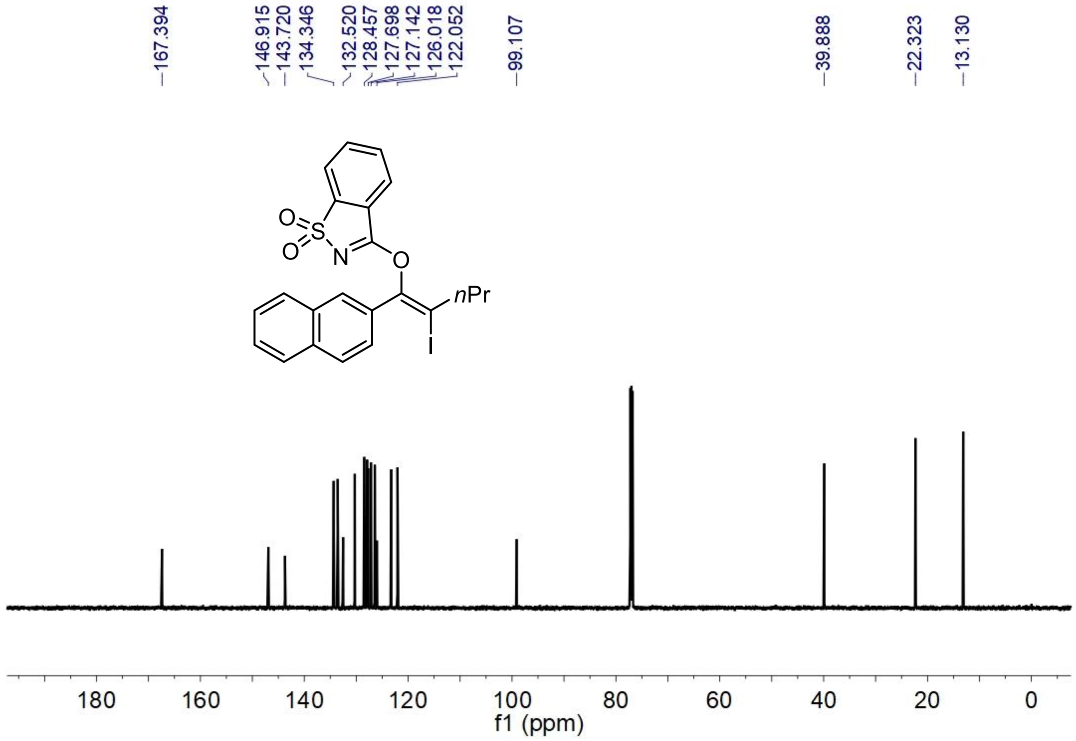

^{13}C NMR (150 MHz, $CDCl_3$) spectrum of 3k.

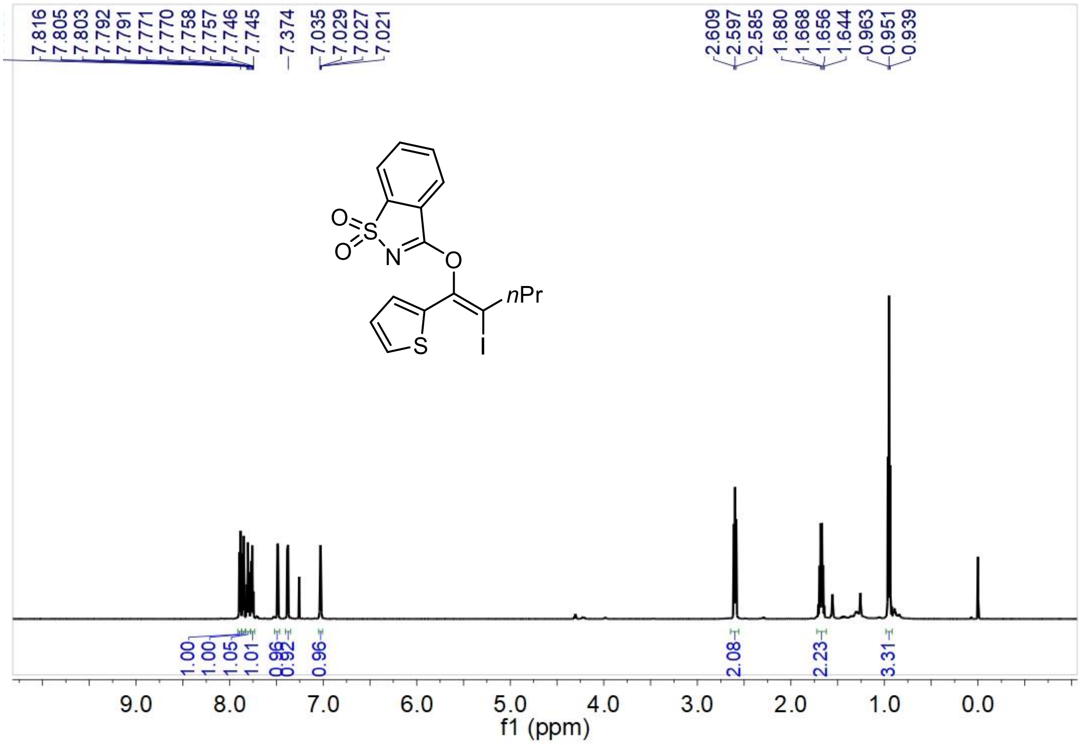

^{1}H NMR (600 MHz, CDCl3) spectrum of 3l.

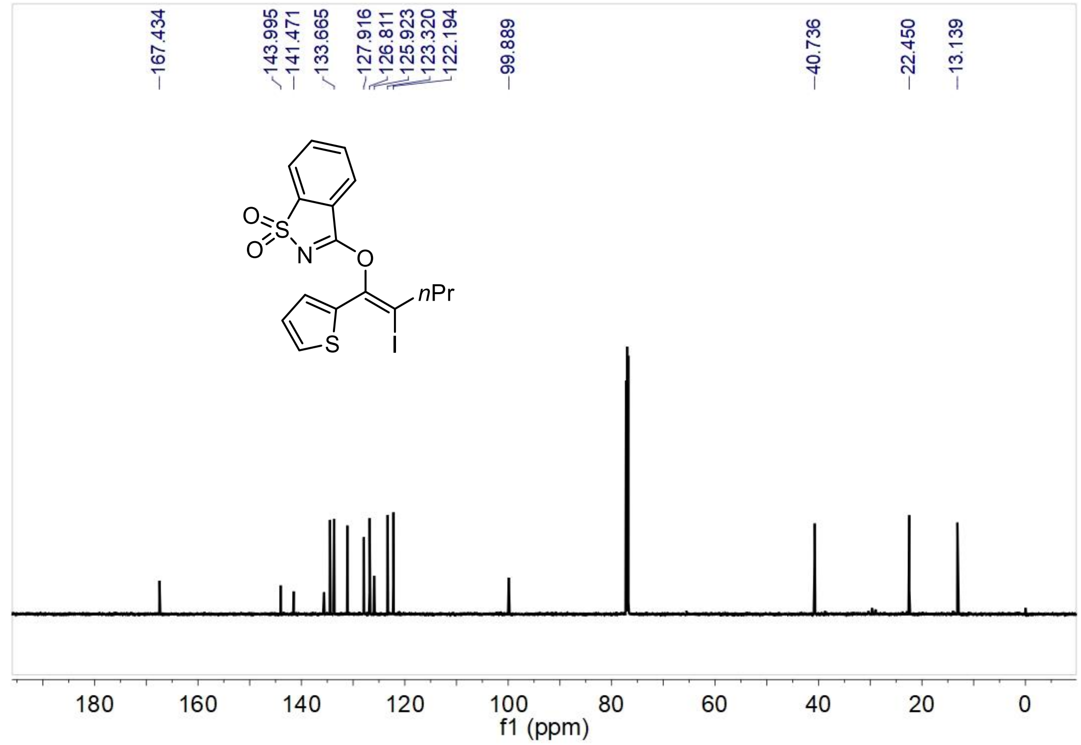

^{13}C NMR (150 MHz, CDCl3) spectrum of 3l.

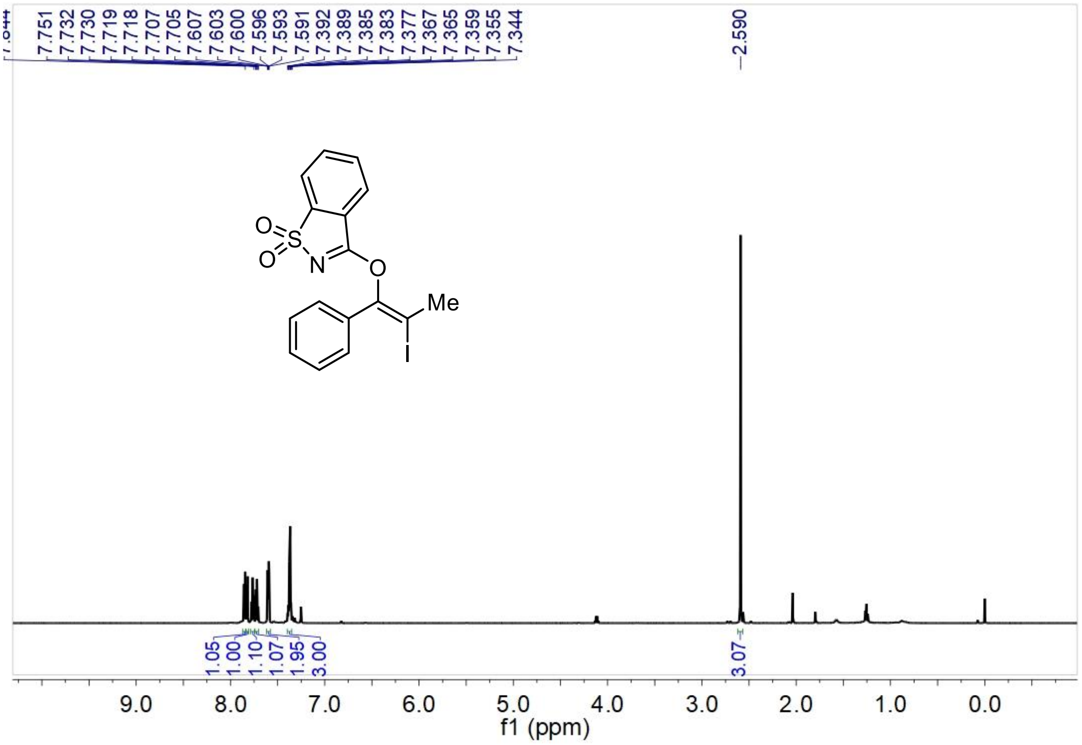

^{1}H NMR (600 MHz, $CDCl_3$) spectrum of 3m.

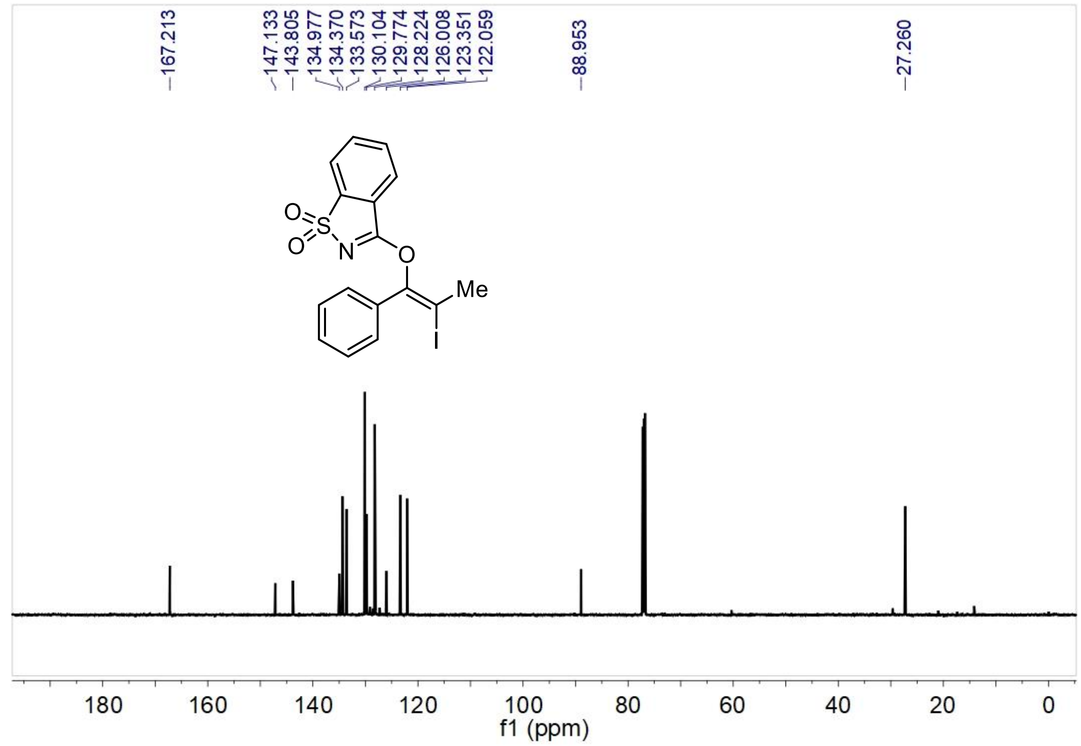

^{13}C NMR (150 MHz, $CDCl_3$) spectrum of 3m.

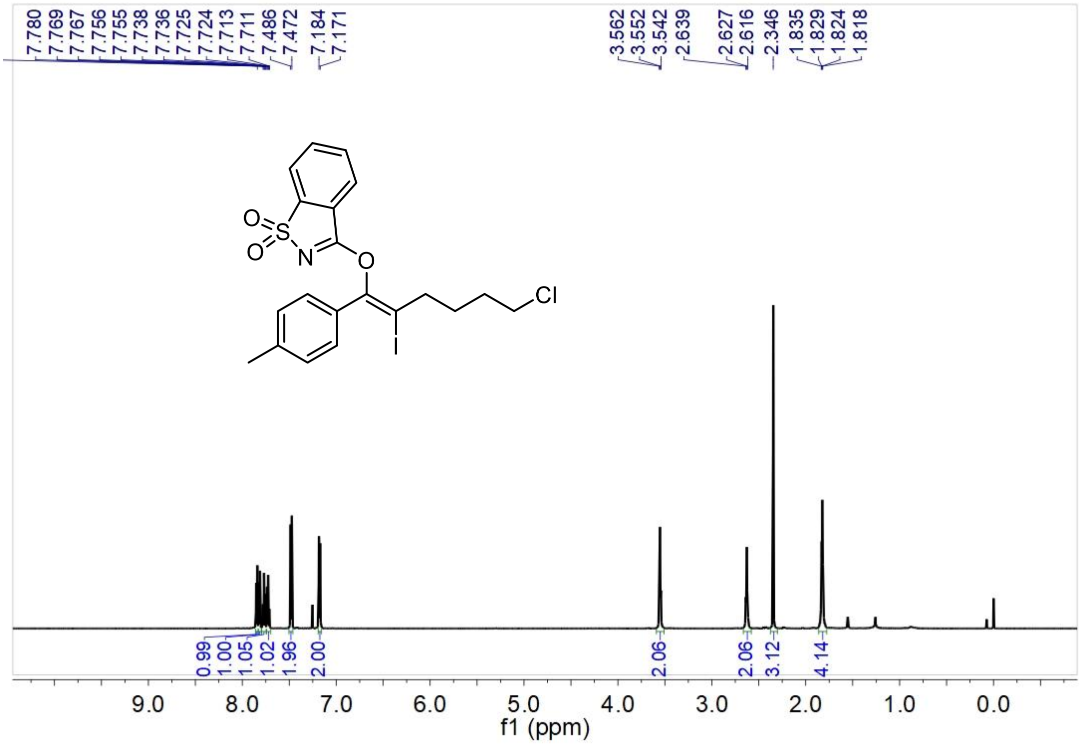

^{1}H NMR (600 MHz, CDCl3) spectrum of 3n.

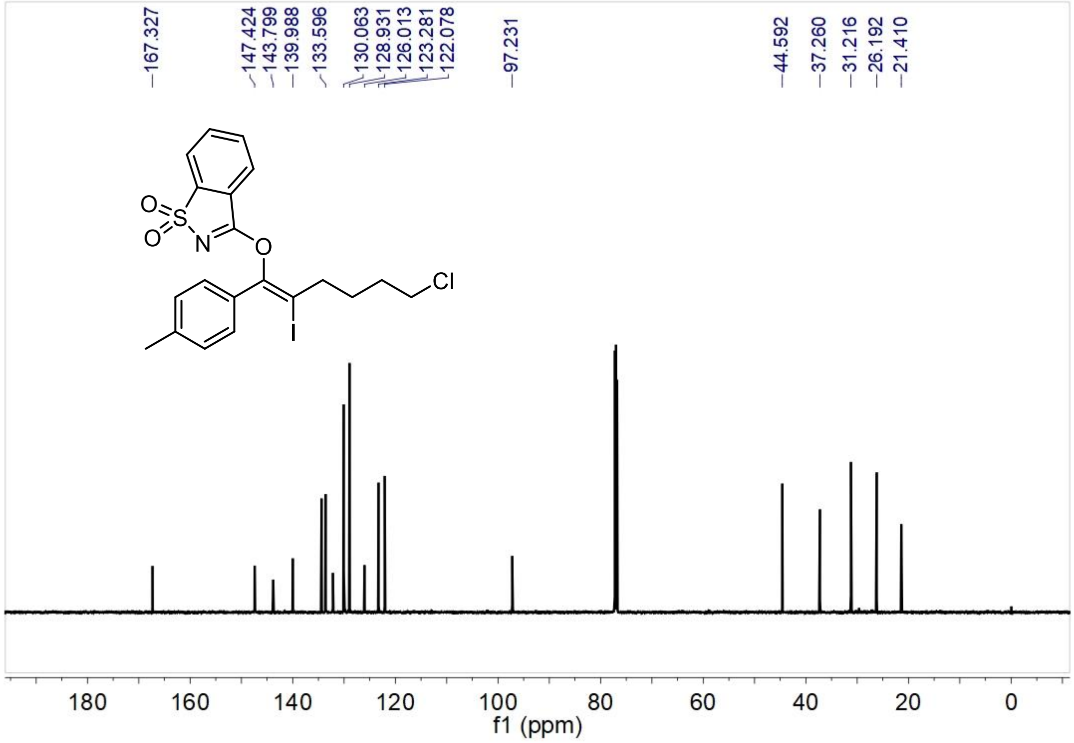

^{13}C NMR (150 MHz, CDCl3) spectrum of 3n.

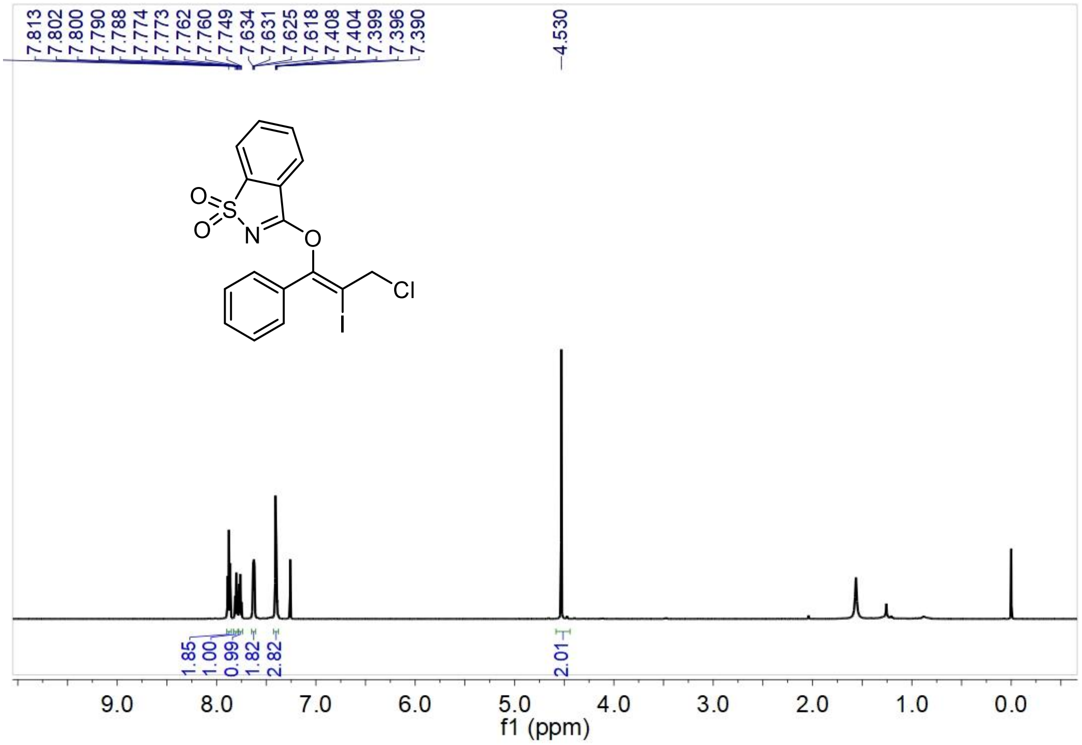

^{1}H NMR (600 MHz, CDCl3) spectrum of 3o.

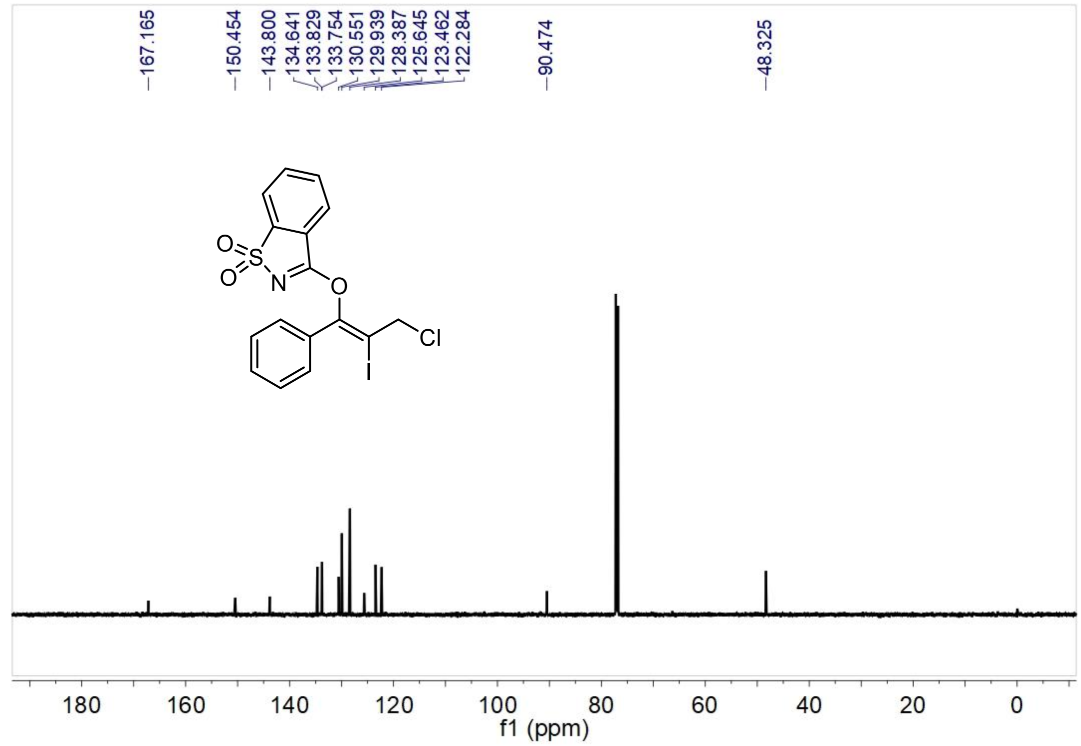

^{13}C NMR (150 MHz, CDCl3) spectrum of 3o.

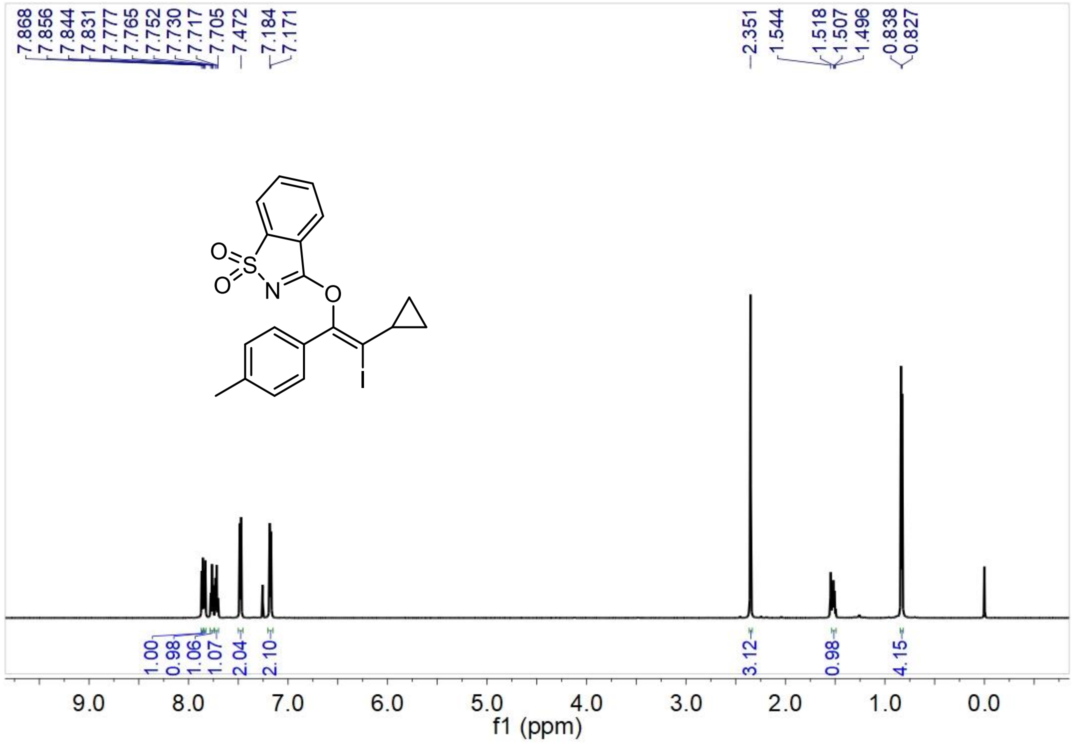

^{1}H NMR (600 MHz, $CDCl_3$) spectrum of 3p.

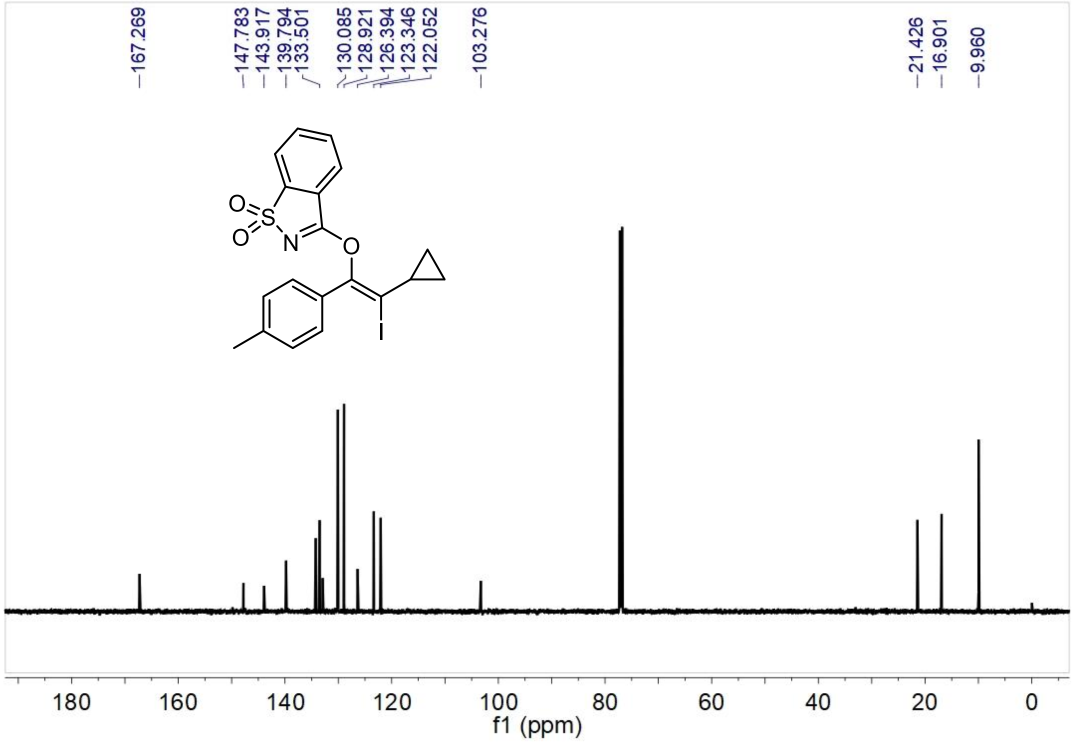

^{13}C NMR (150 MHz, $CDCl_3$) spectrum of 3p.

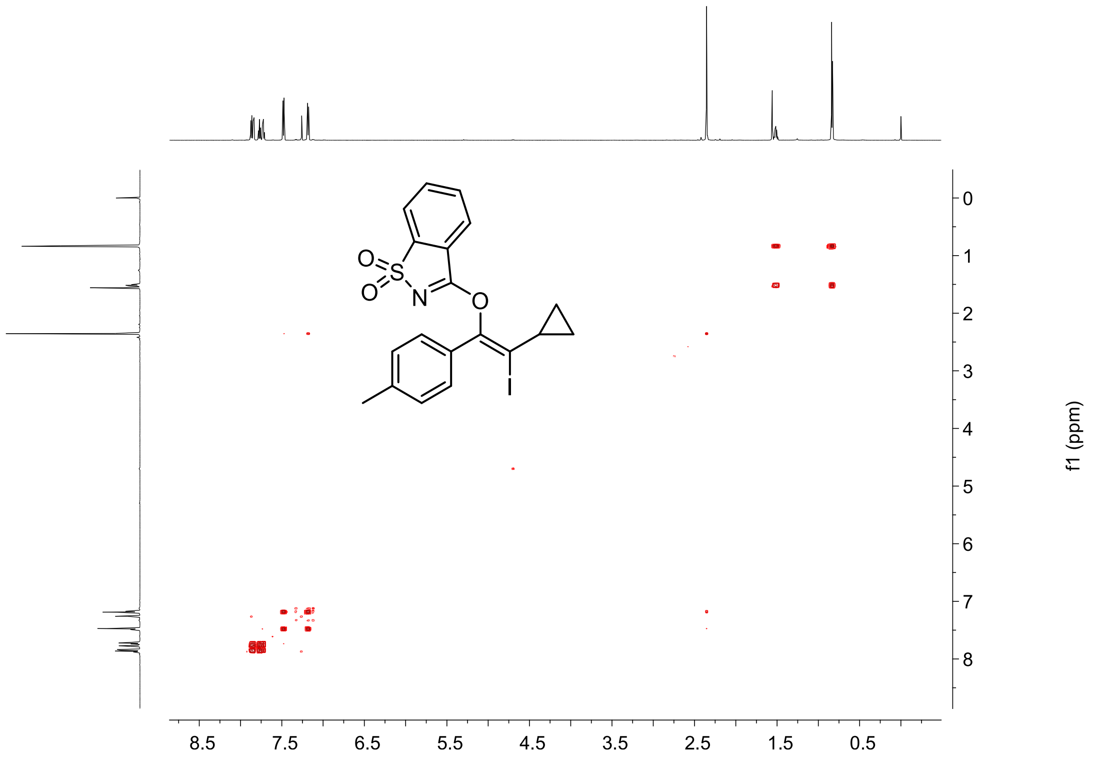

^{1}H-^{1}H COSY spectrum of 3p.

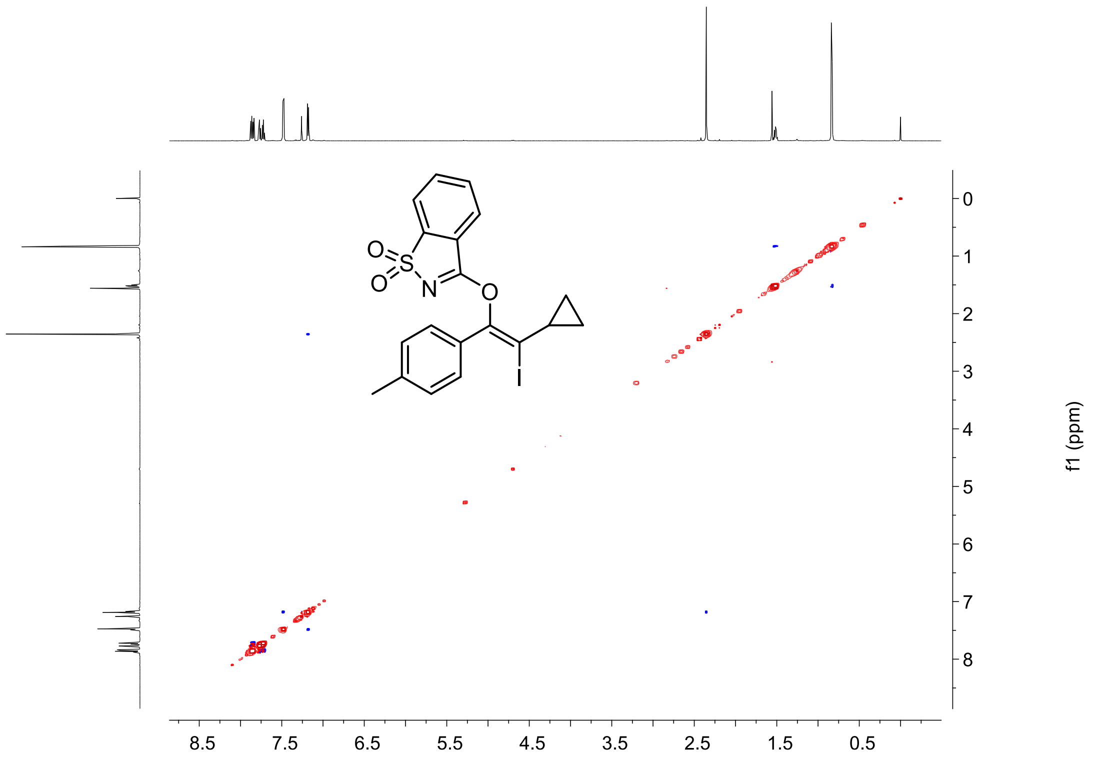

^{1}H-^{1}H NOESY spectrum of 3p.

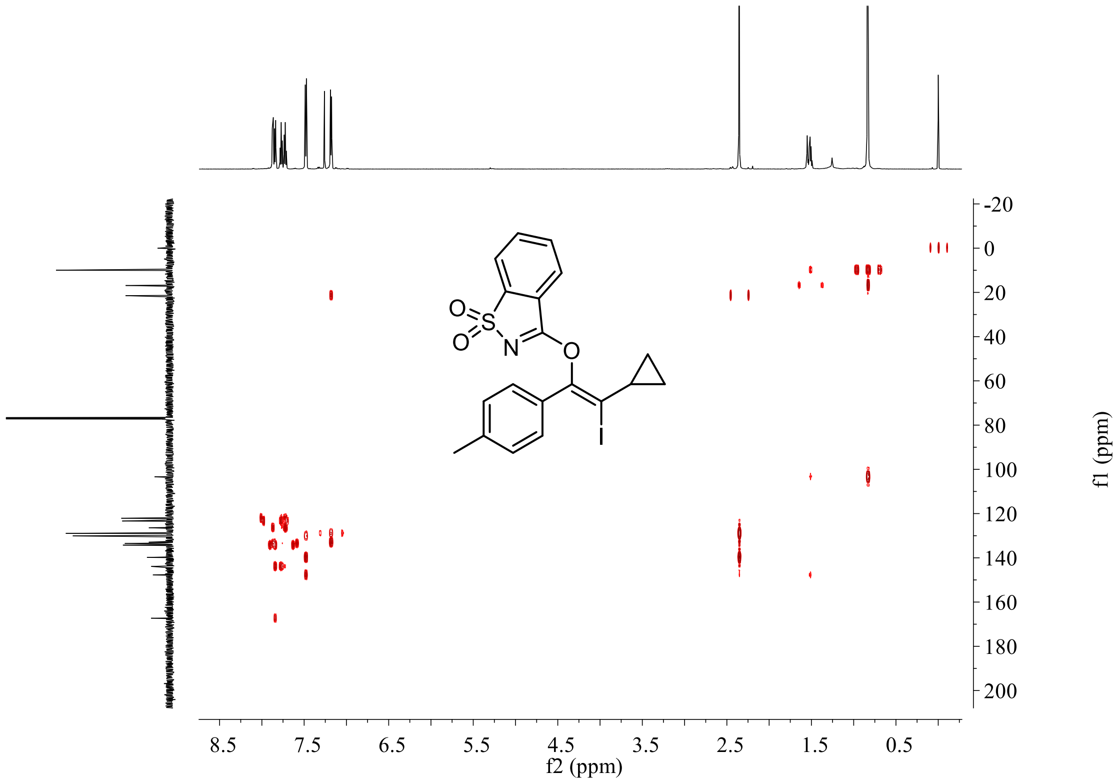

^{1}H-^{13}C HMQC spectrum of 3p.

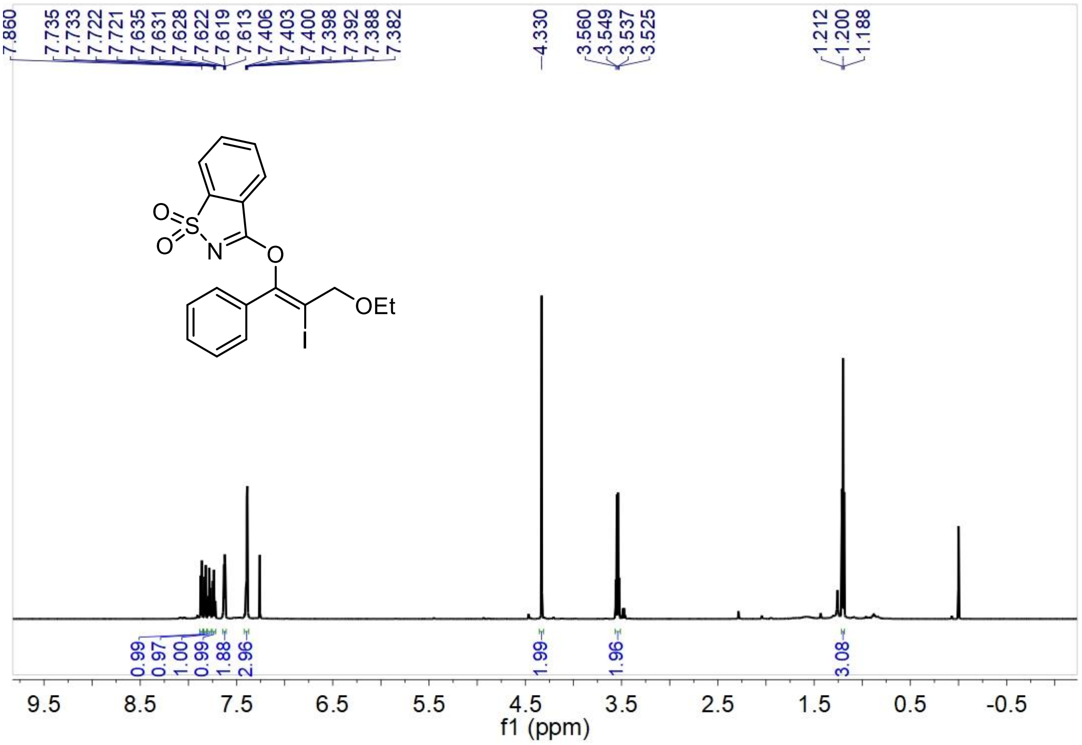

^{1}H NMR (600 MHz, CDCl3) spectrum of 3q.

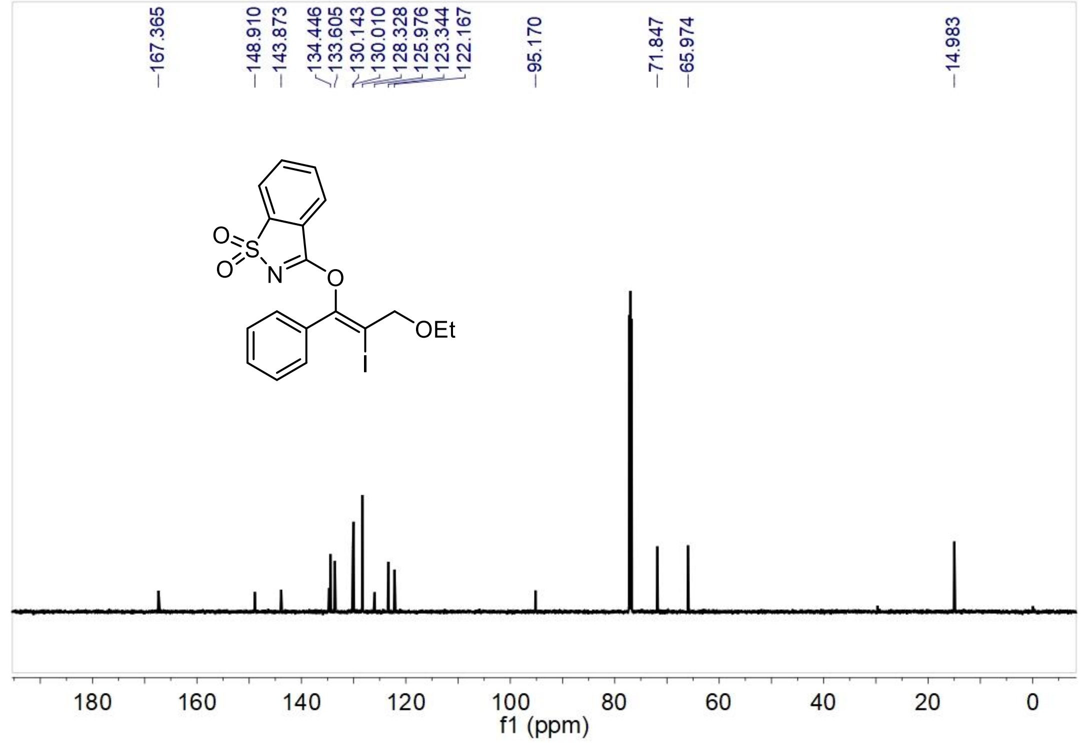

^{13}C NMR (150 MHz, CDCl3) spectrum of 3q.

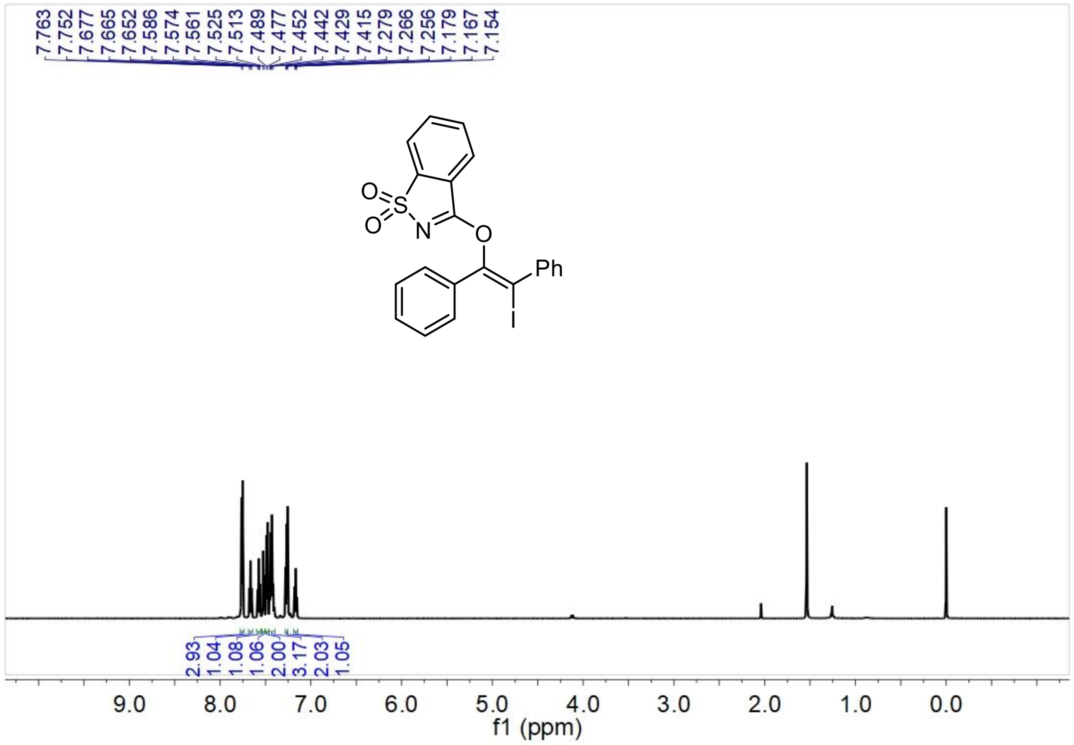

^{1}H NMR (600 MHz, CDCl3) spectrum of 3r.

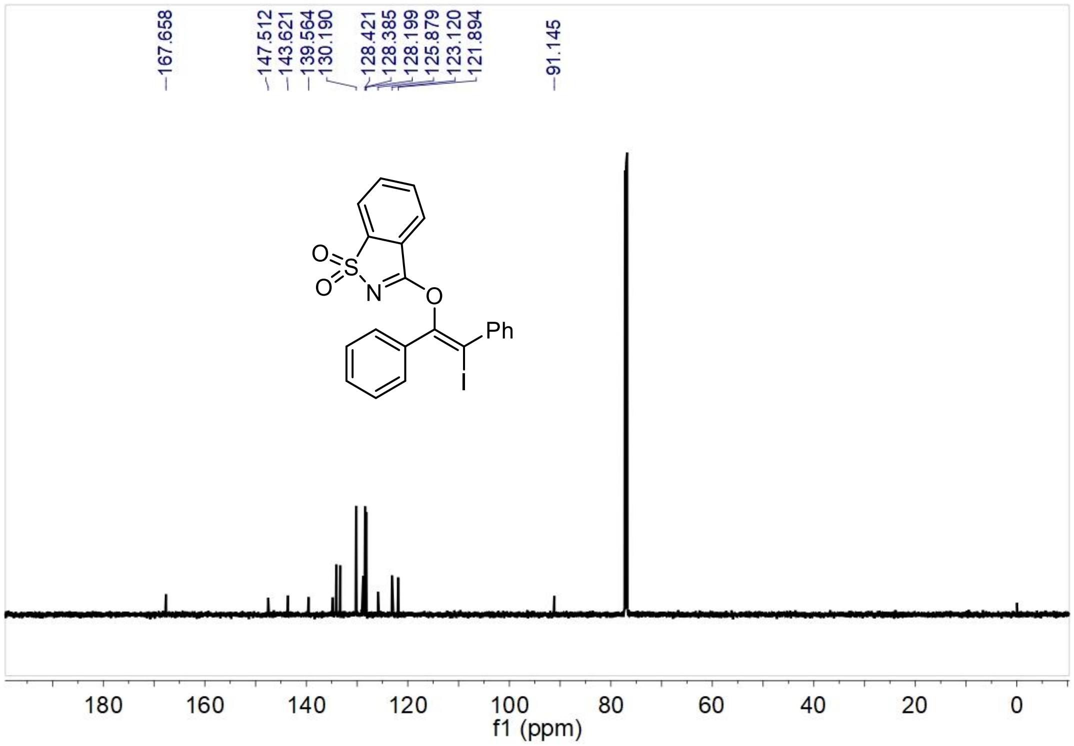

^{13}C NMR (150 MHz, CDCl3) spectrum of 3r.

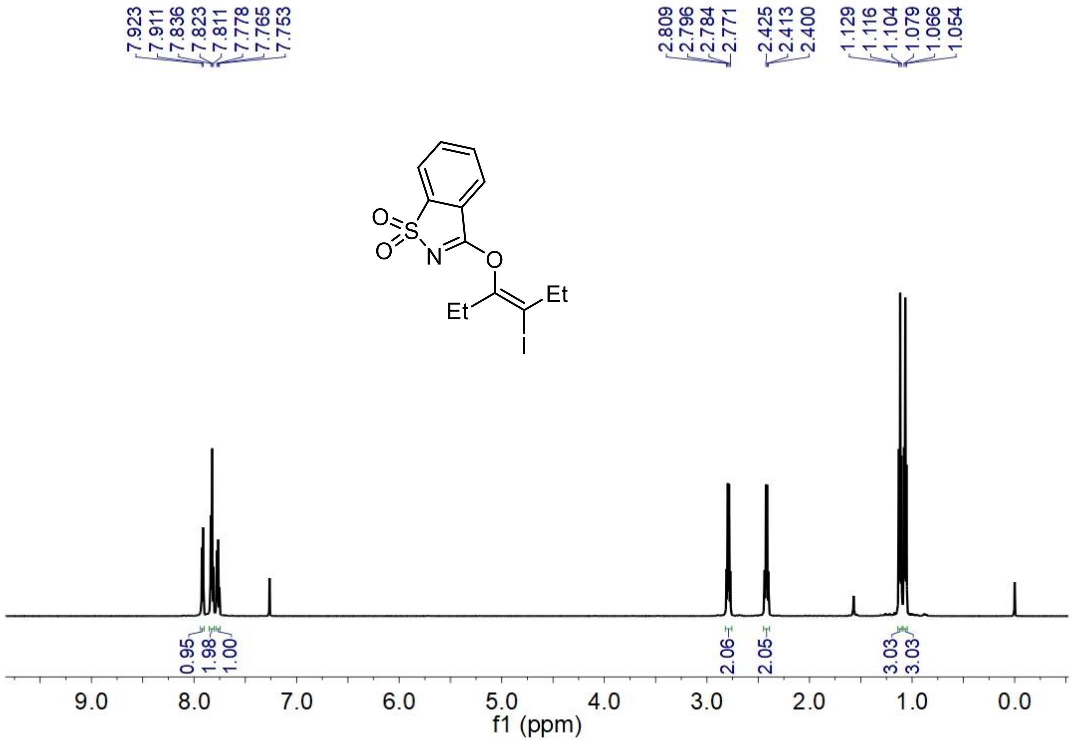

^{1}H NMR (600 MHz, CDCl3) spectrum of 3s.

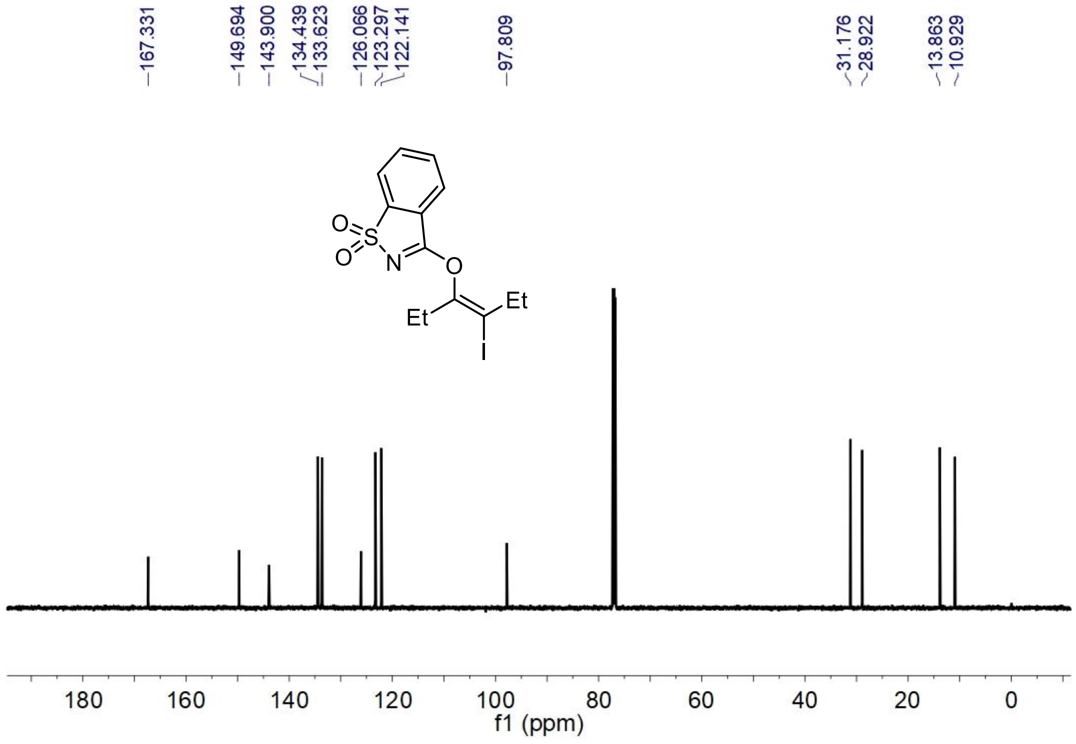

^{13}C NMR (150 MHz, CDCl3) spectrum of 3s.

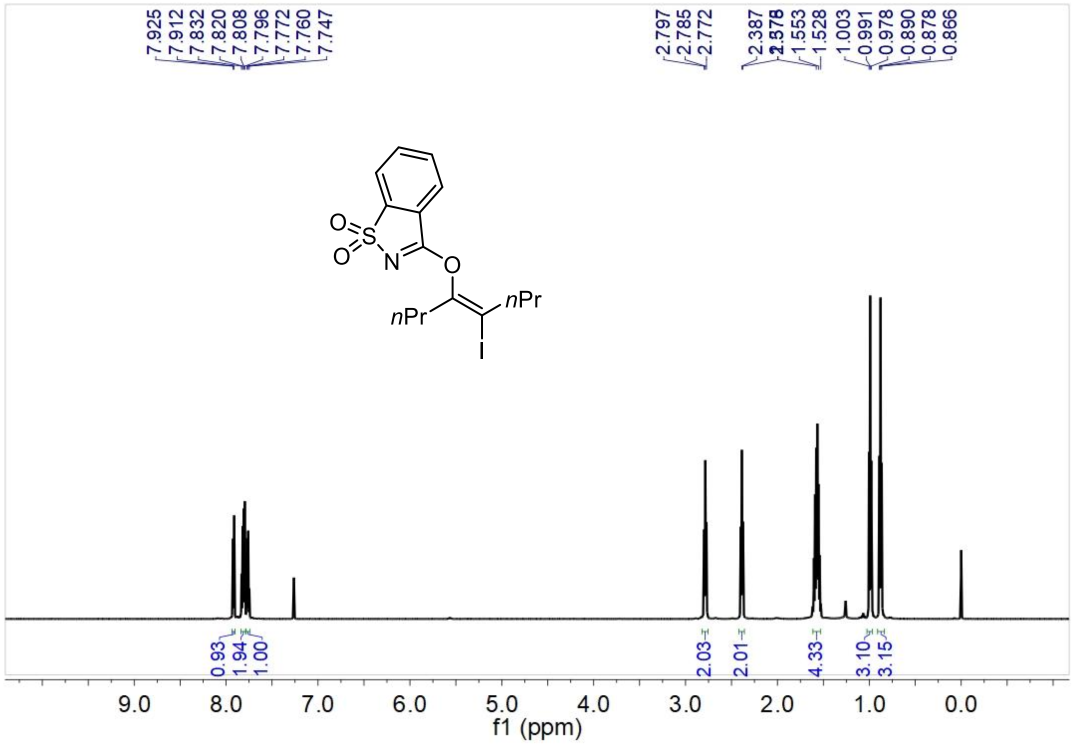

^{1}H NMR (600 MHz, $CDCl_3$) spectrum of 3t.

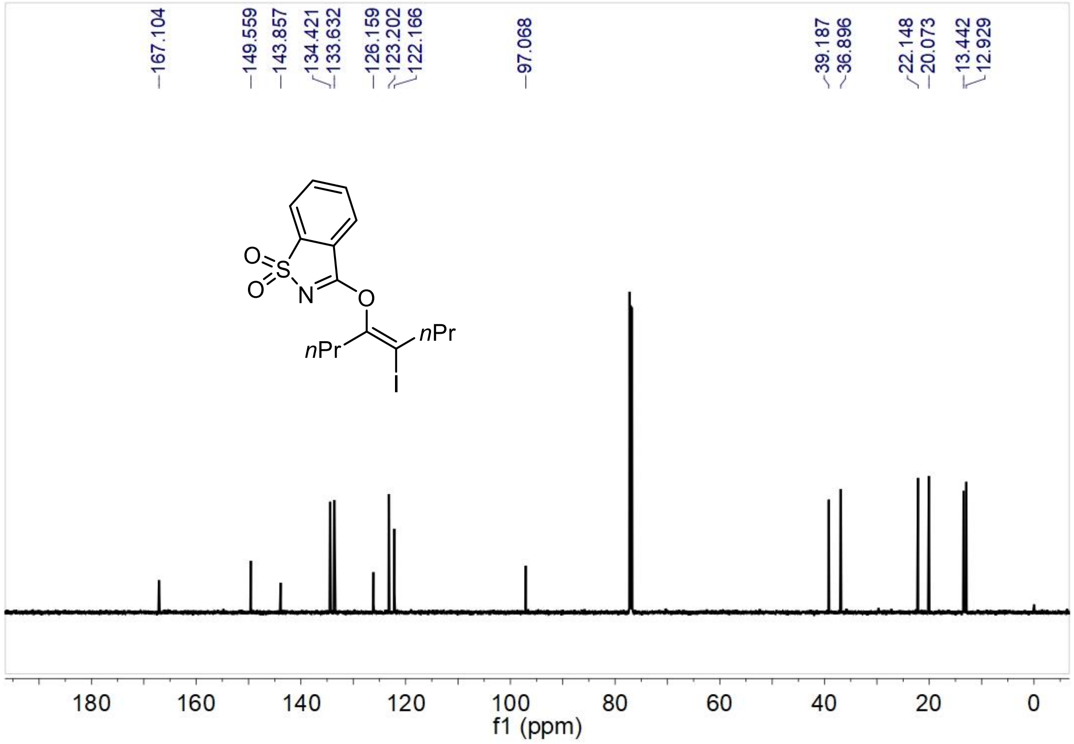

^{13}C NMR (150 MHz, $CDCl_3$) spectrum of 3t.

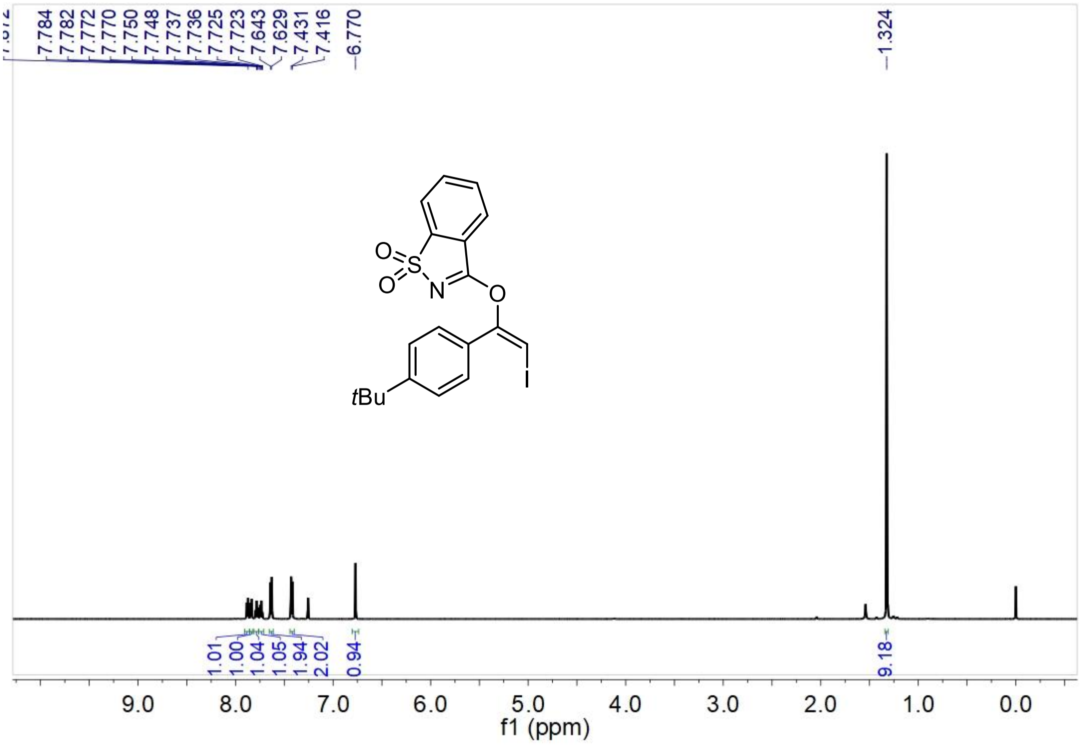

^{1}H NMR (600 MHz, CDCl3) spectrum of 3u.

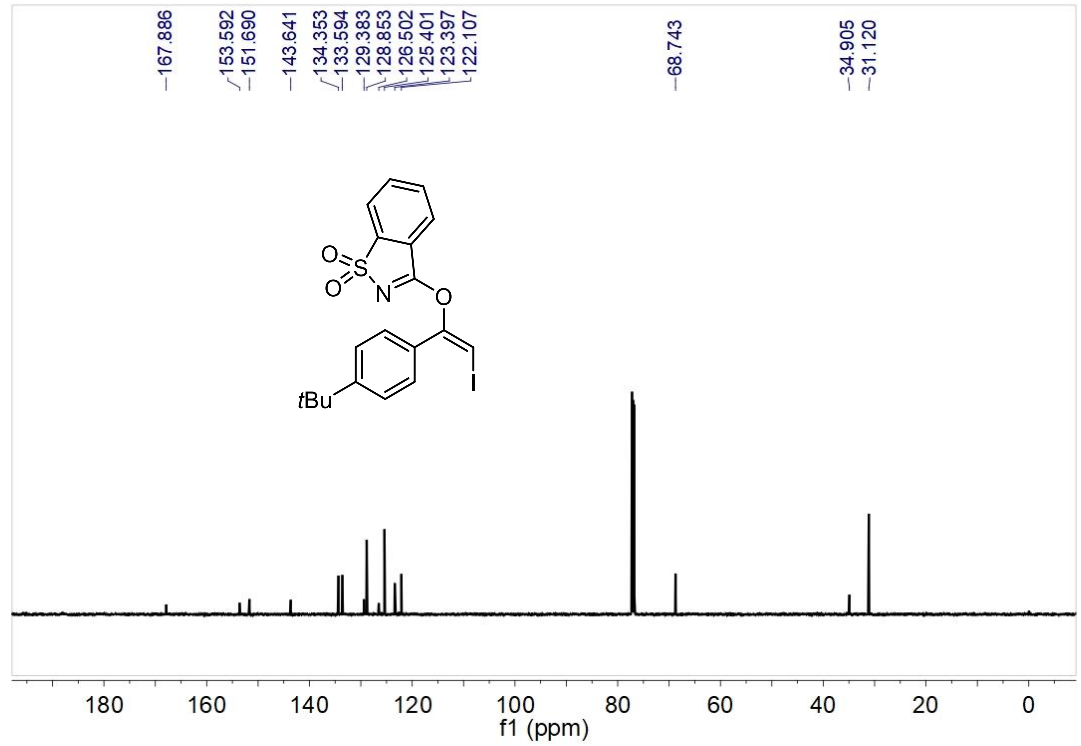

^{13}C NMR (150 MHz, CDCl3) spectrum of 3u.

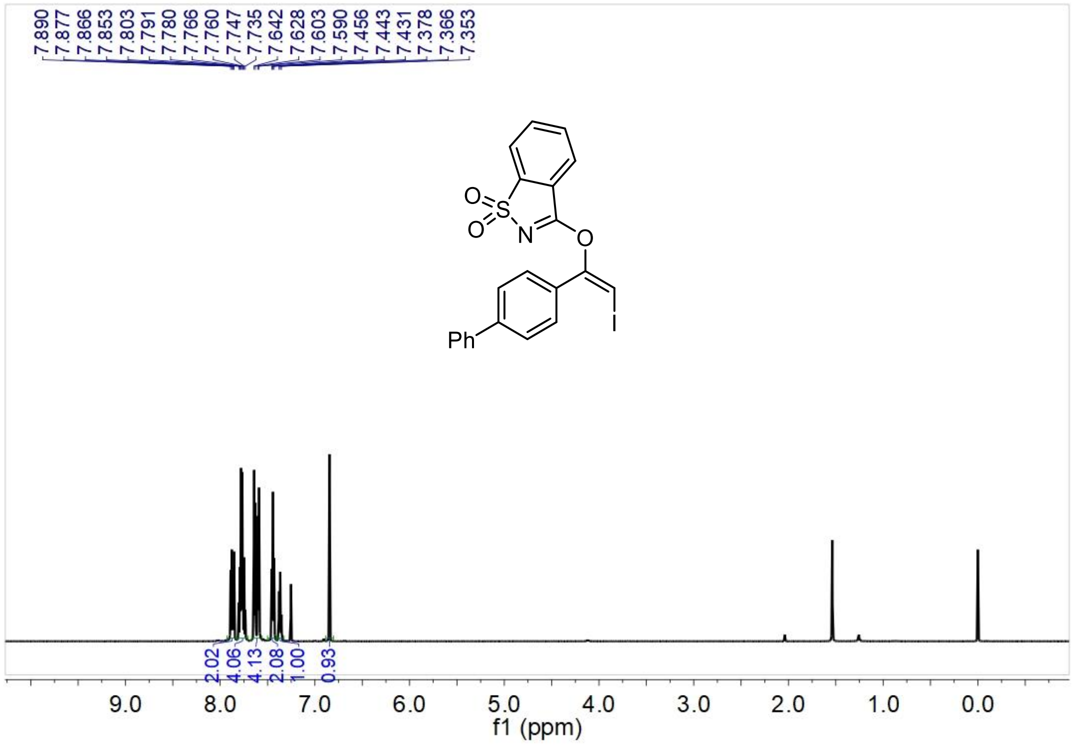

^{1}H NMR (600 MHz, CDCl$_3$) spectrum of 3v.

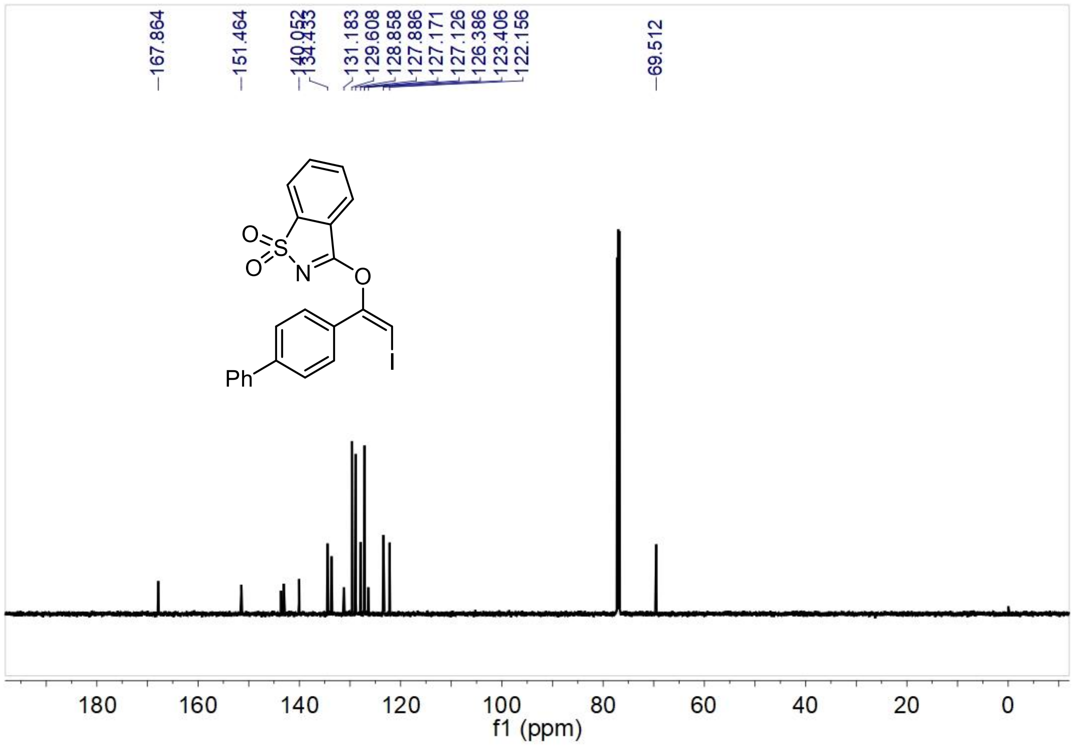

^{13}C NMR (150 MHz, CDCl$_3$) spectrum of 3v.

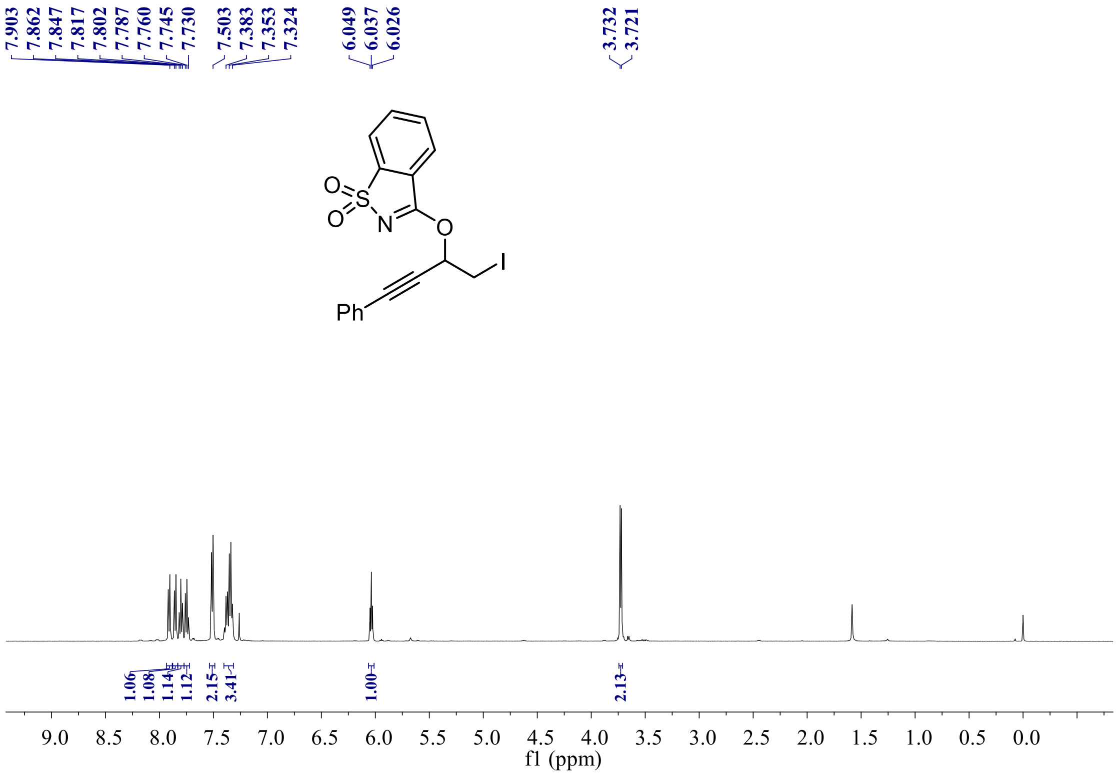

^{1}H NMR (500 MHz, $CDCl_3$) spectrum of 4a.

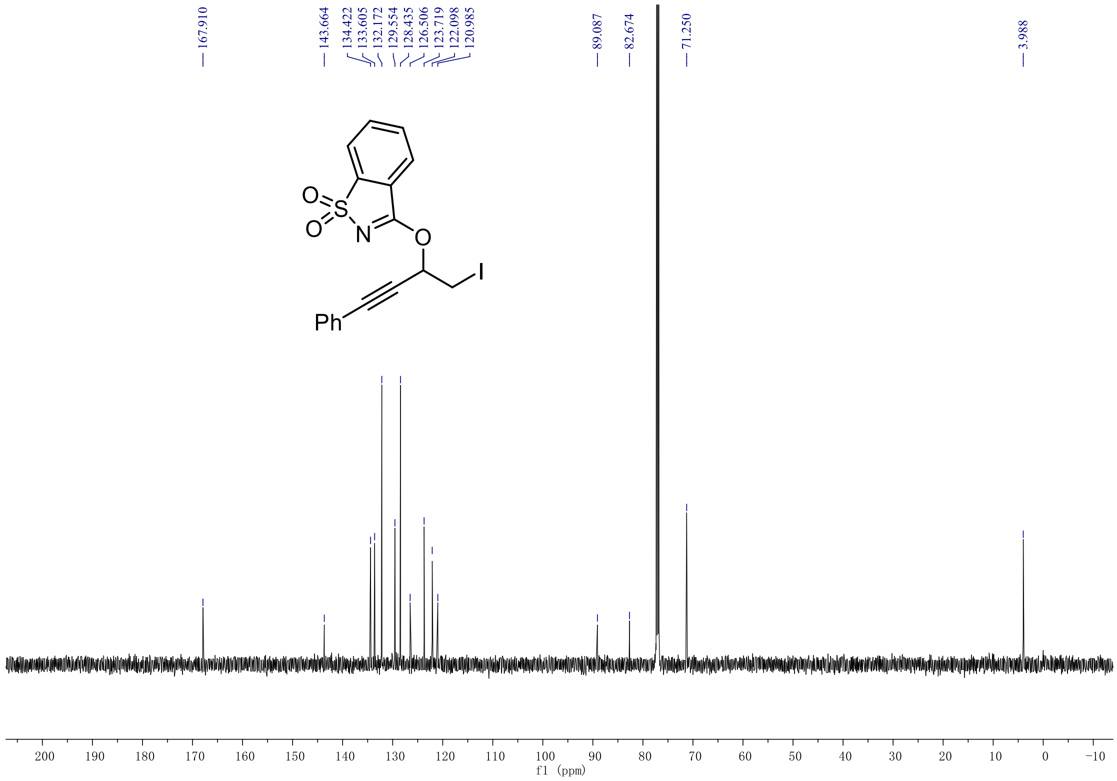

^{13}C NMR (150 MHz, $CDCl_3$) spectrum of 4a.

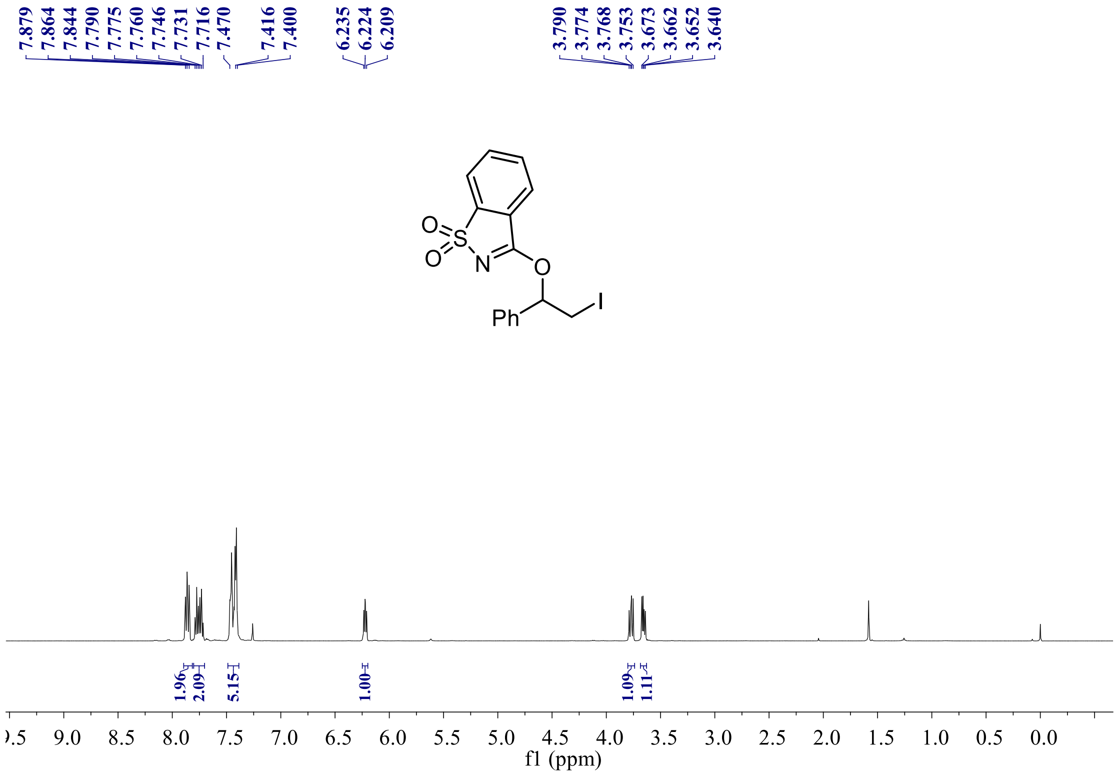

^{1}H NMR (500 MHz, CDCl3) spectrum of 4b.

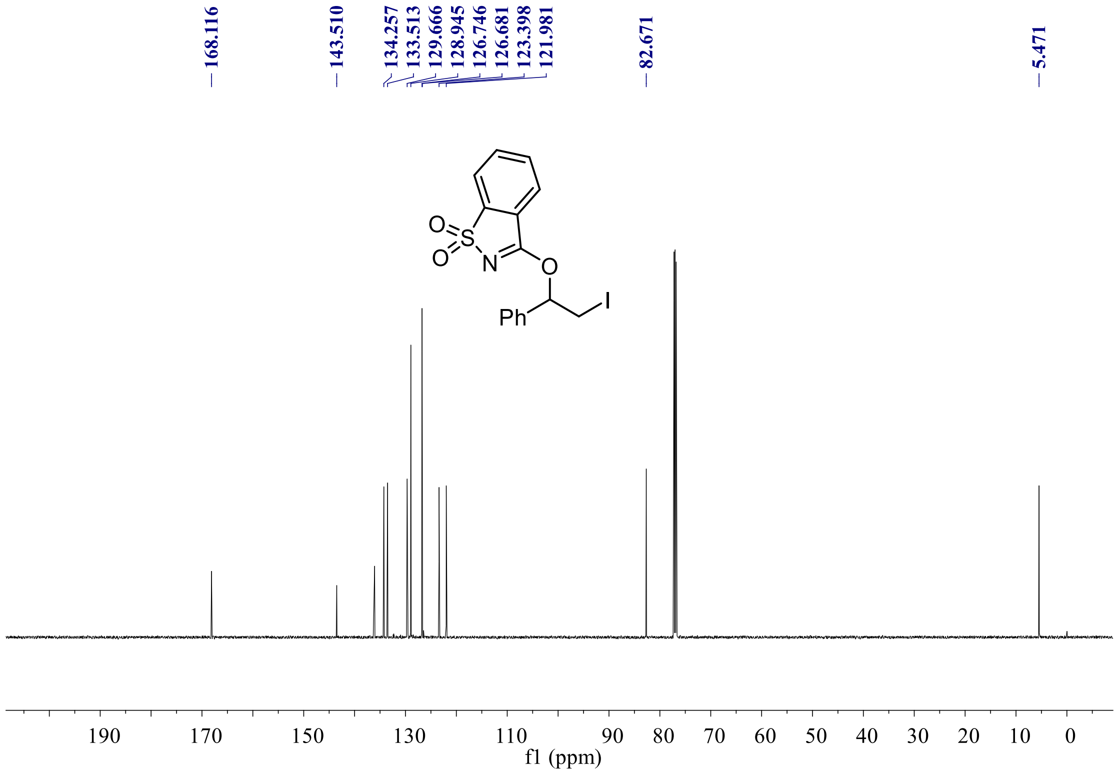

^{1}H NMR (500 MHz, CDCl3) spectrum of 4b.

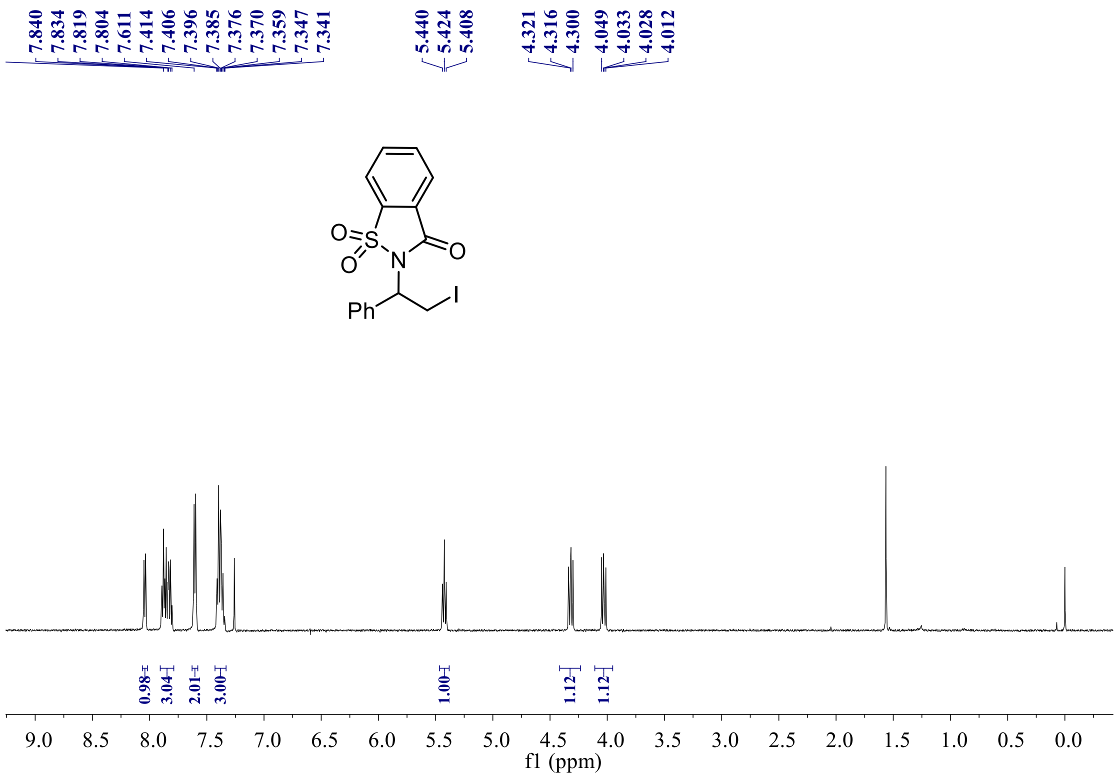

^{1}H NMR (500 MHz, CDCl3) spectrum of 4b'.

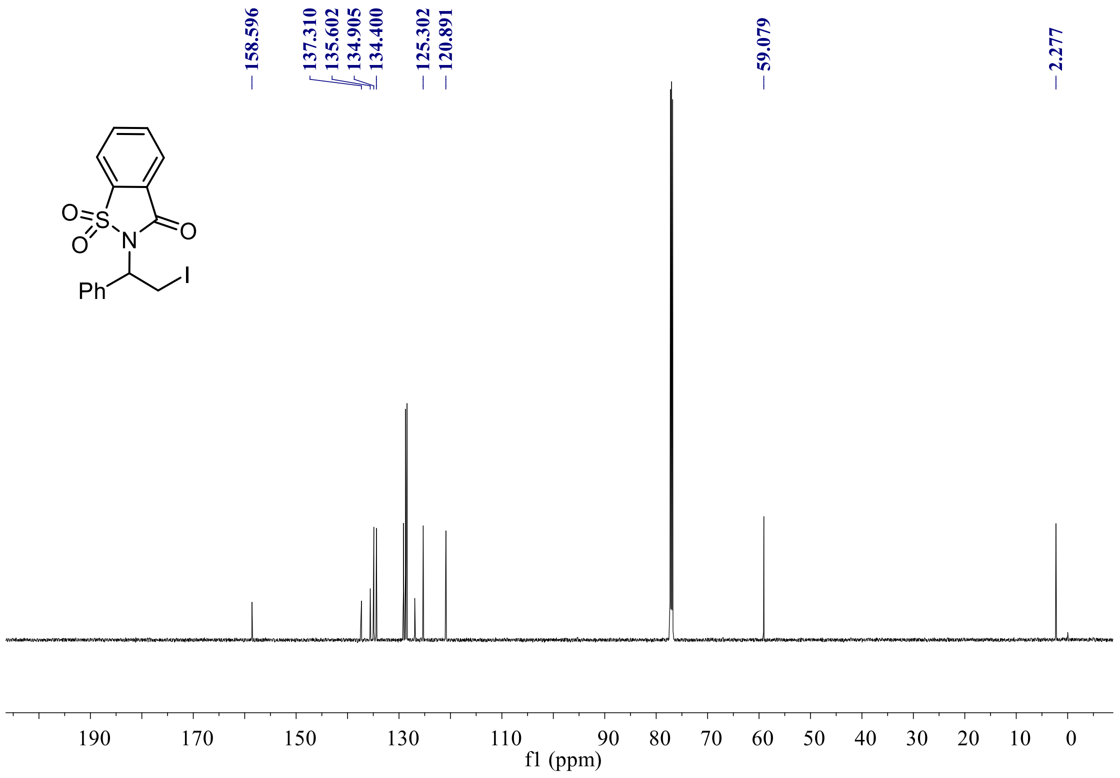

^{13}C NMR (150 MHz, CDCl3) spectrum of 4b'.

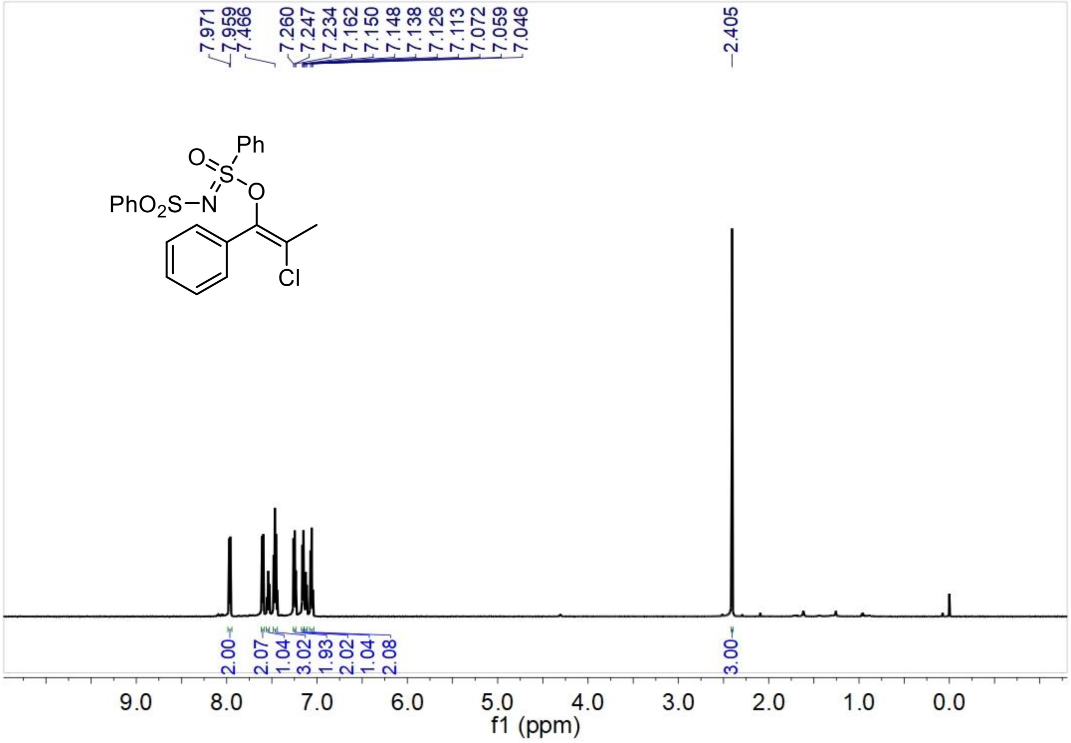

^{1}H NMR (600 MHz, CDCl$_3$) spectrum of 5a.

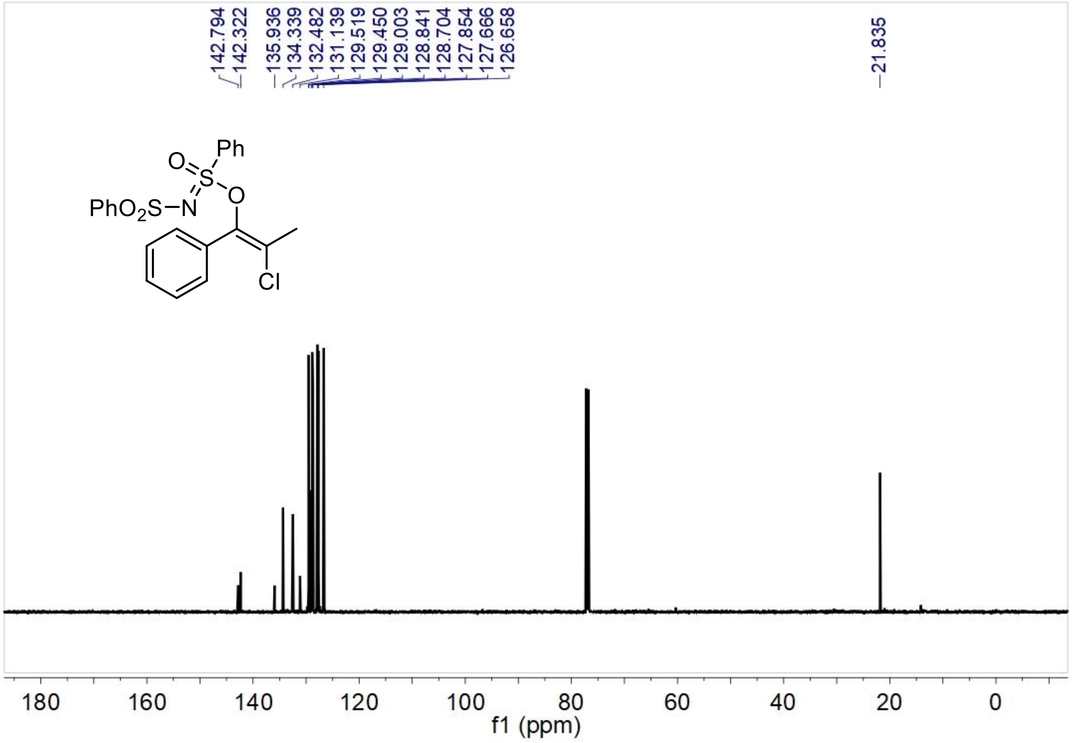

^{13}C NMR (150 MHz, CDCl$_3$) spectrum of 5a.

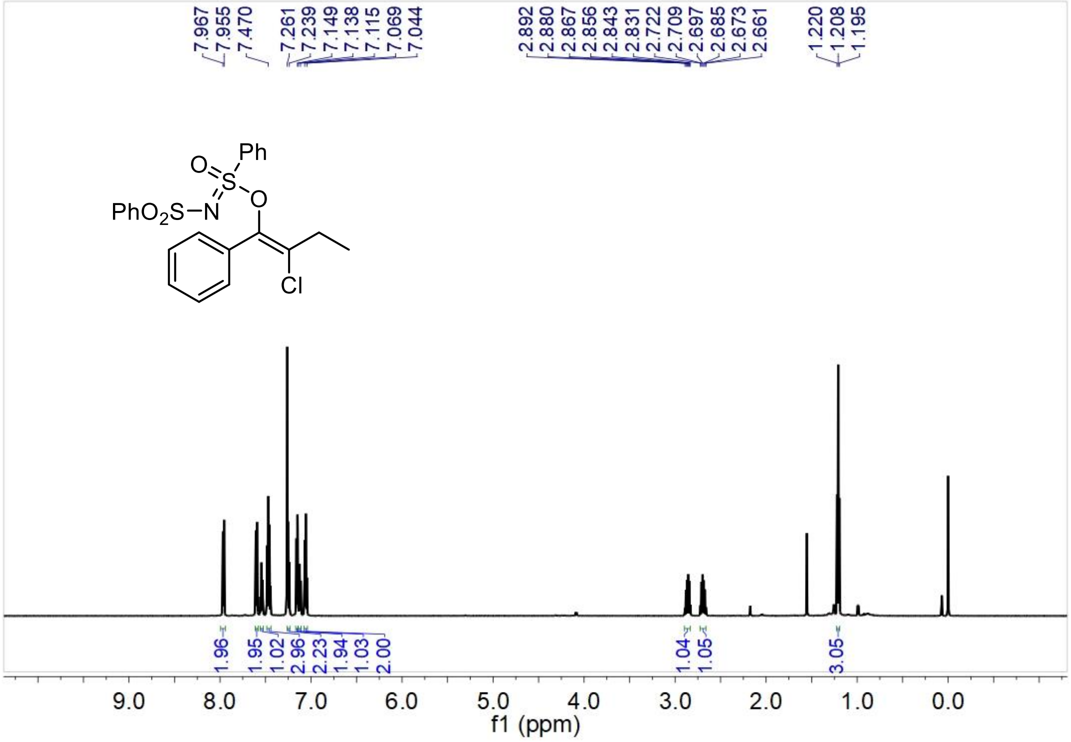

^{1}H NMR (600 MHz, CDCl$_3$) spectrum of 5b.

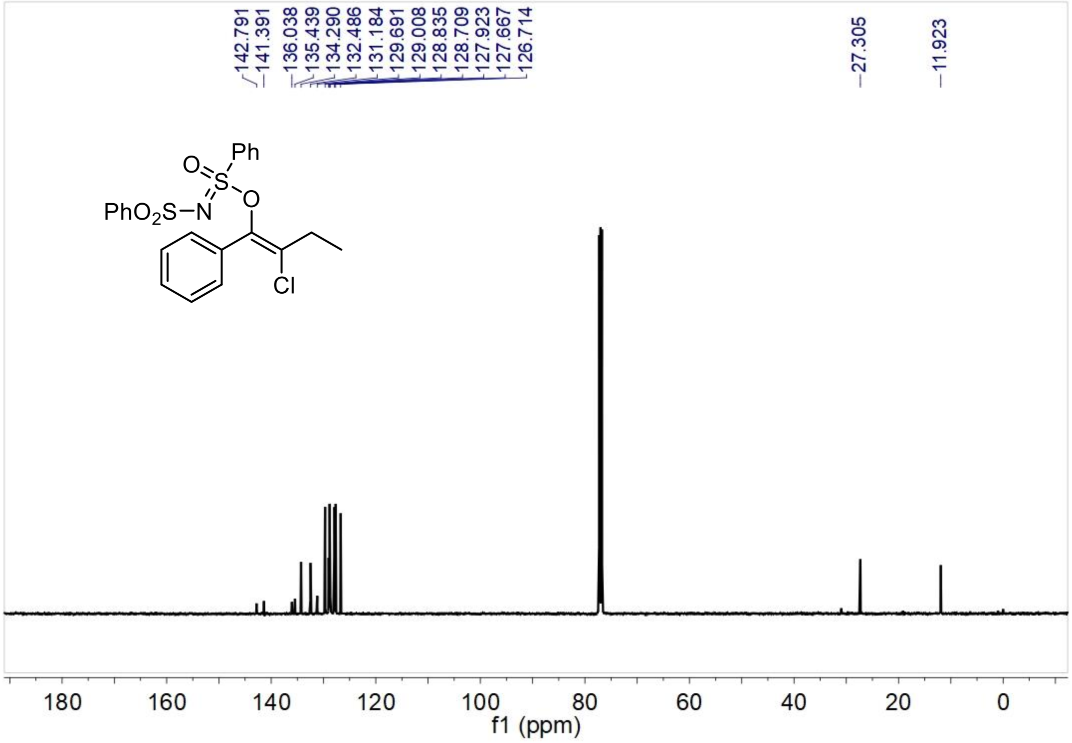

^{13}C NMR (125 MHz, CDCl$_3$) spectrum of 5b.

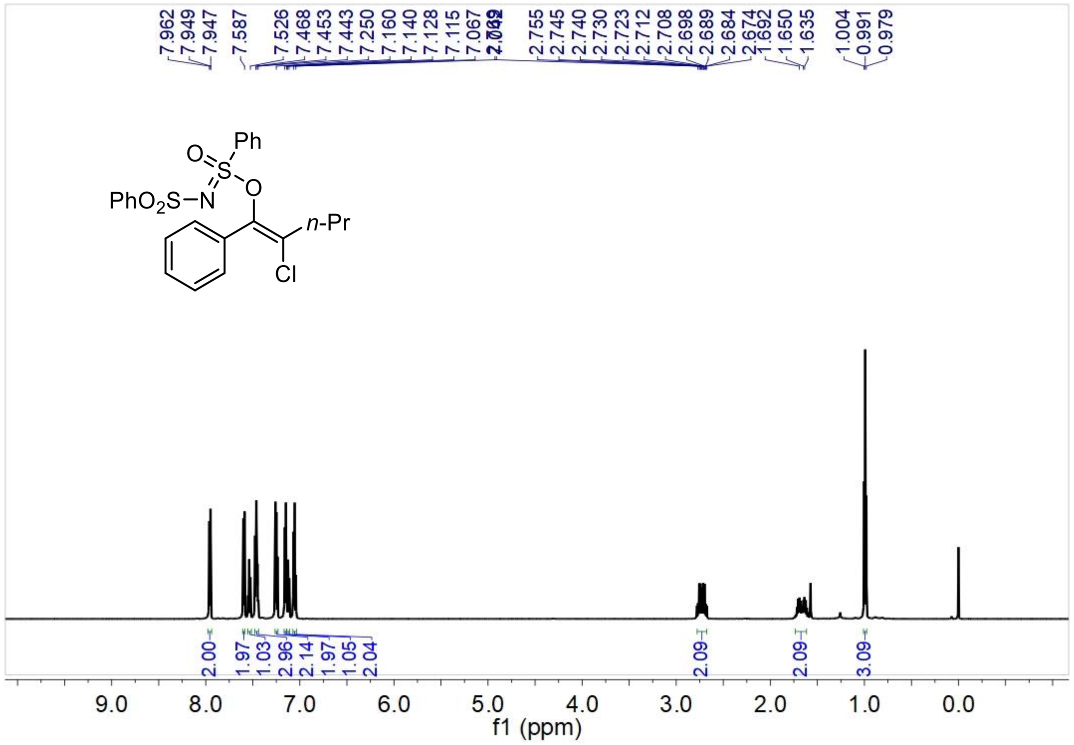

^{1}H NMR (600 MHz, CDCl3) spectrum of 5c.

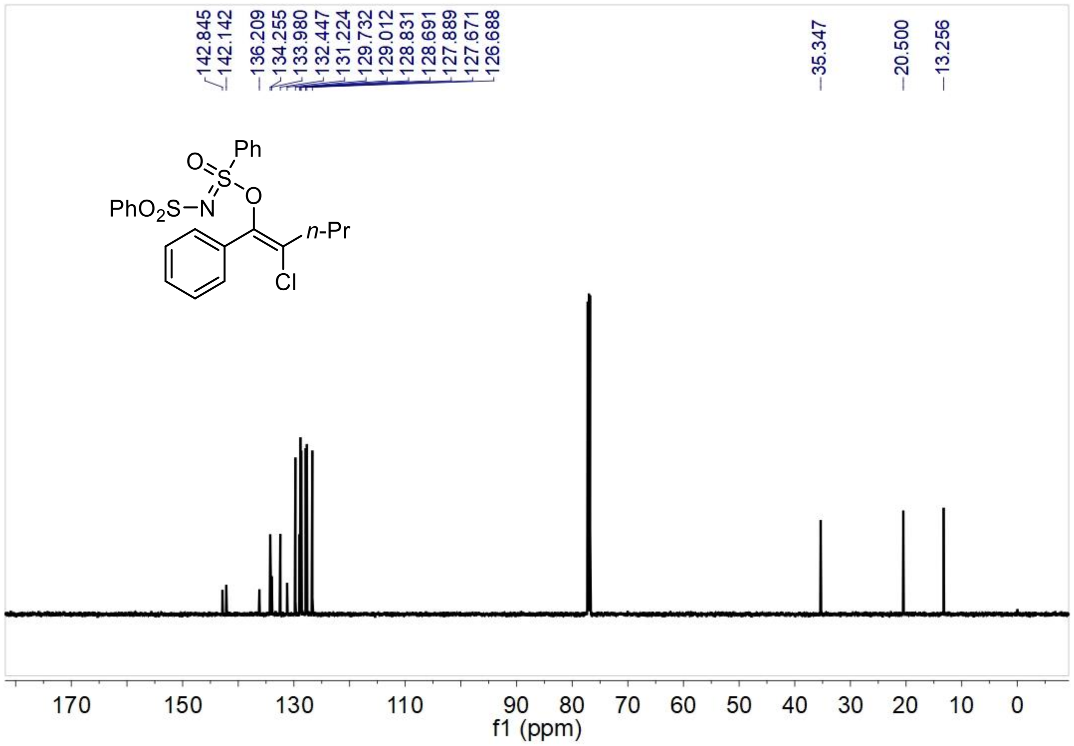

^{13}C NMR (150 MHz, CDCl3) spectrum of 5c.

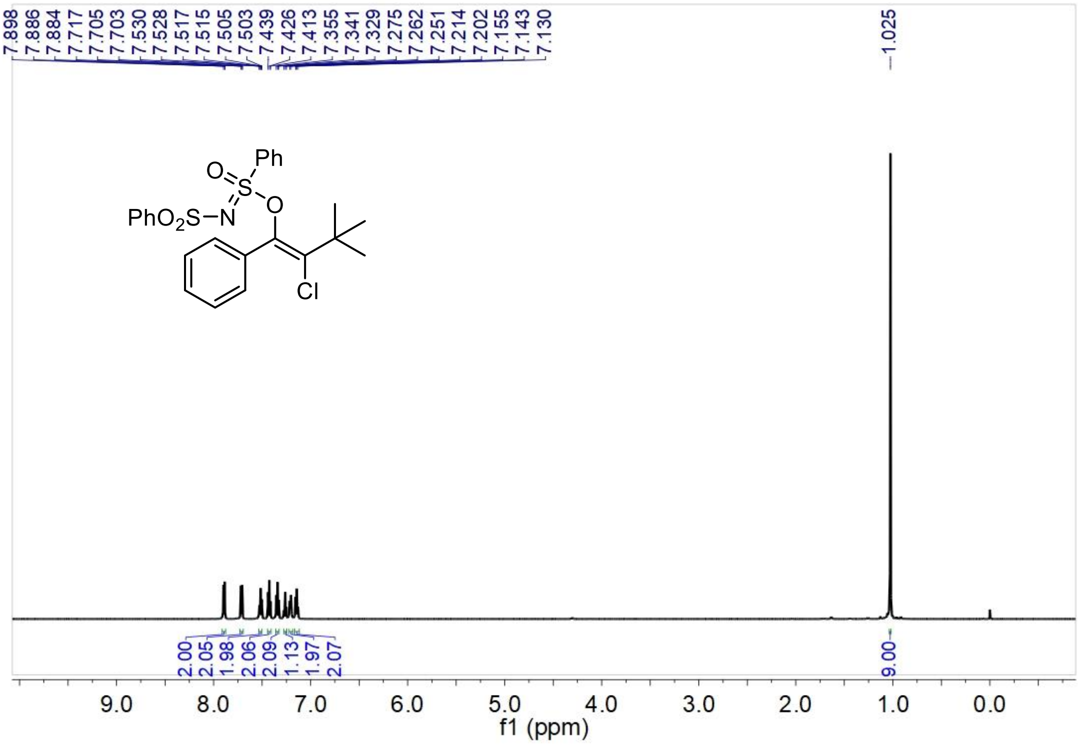

^{1}H NMR (600 MHz, $CDCl_3$) spectrum of 5d.

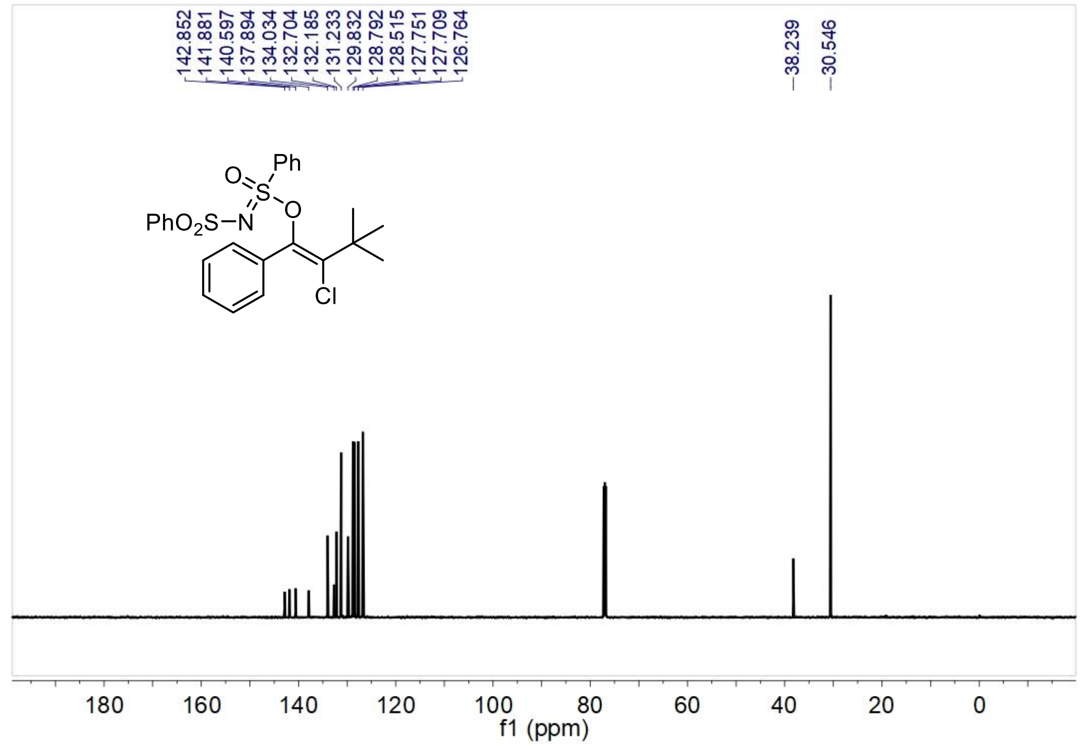

^{13}C NMR (150 MHz, $CDCl_3$) spectrum of 5d.

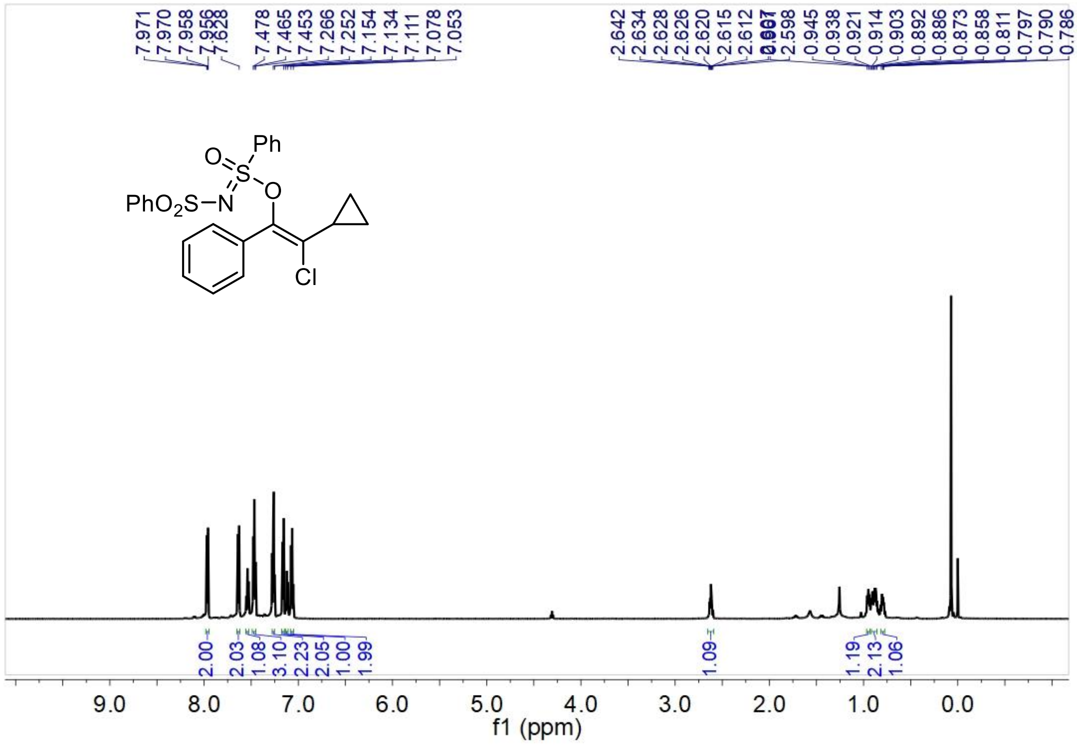

^{1}H NMR (600 MHz, CDCl3) spectrum of 5e.

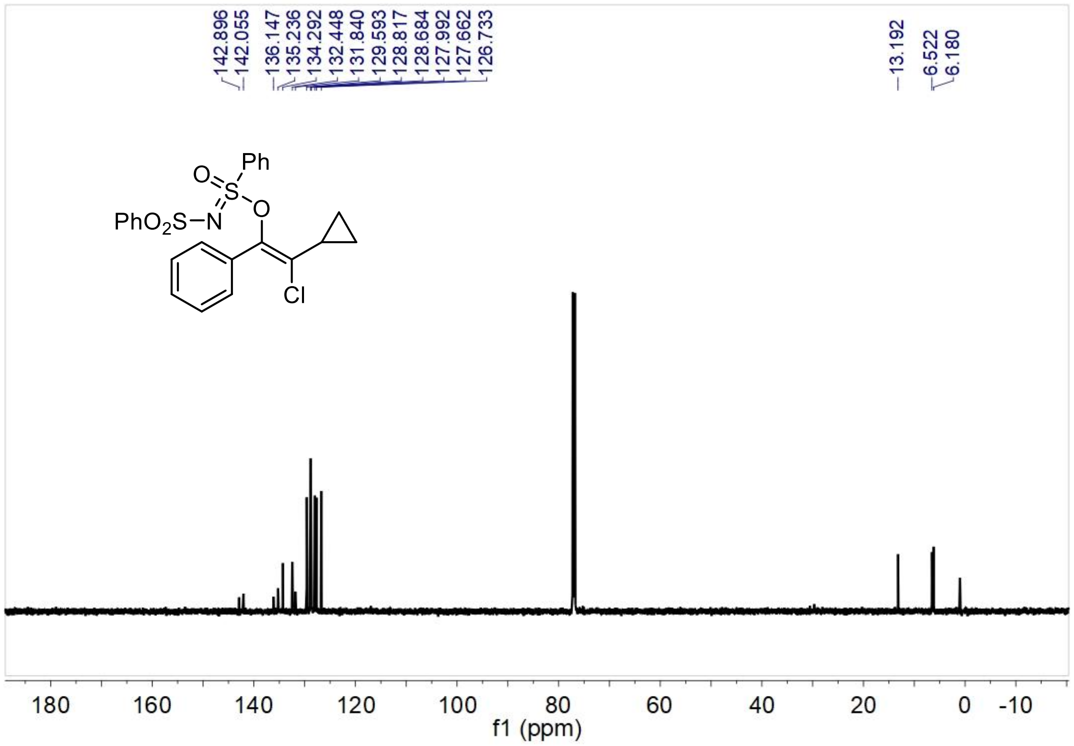

^{13}C NMR (150 MHz, CDCl3) spectrum of 5e.

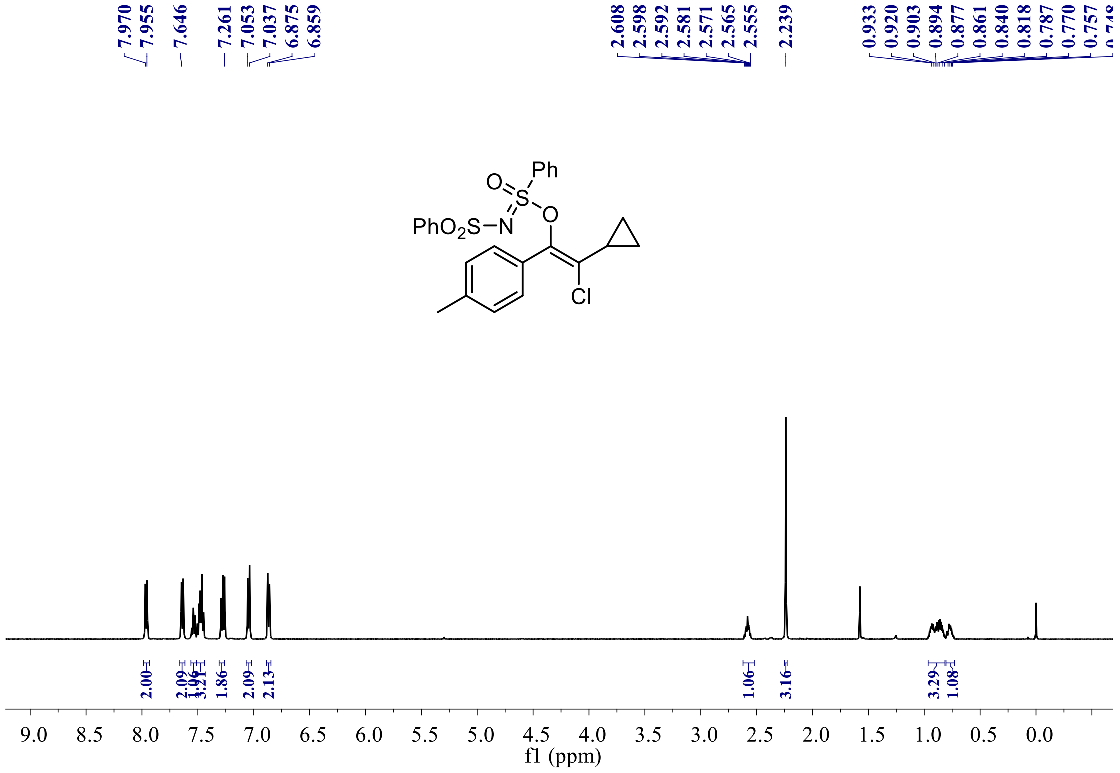

^{1}H NMR (500 MHz, $CDCl_3$) spectrum of 5f.

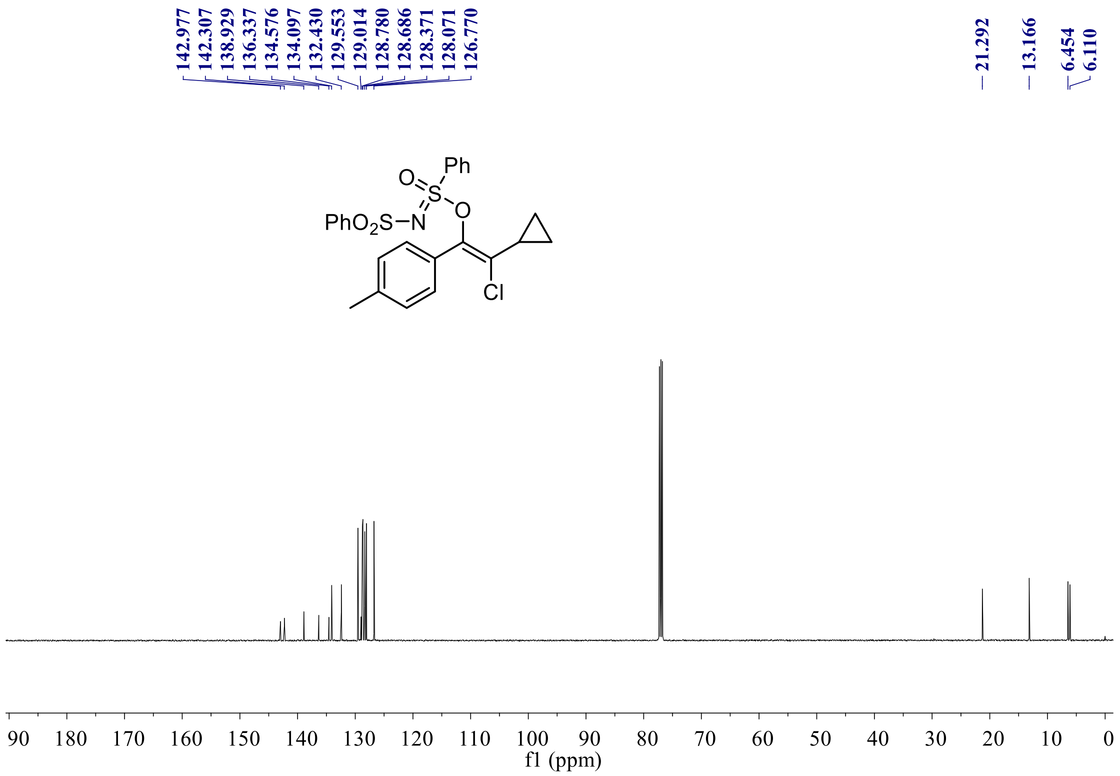

^{13}C NMR (150 MHz, $CDCl_3$) spectrum of 5f.

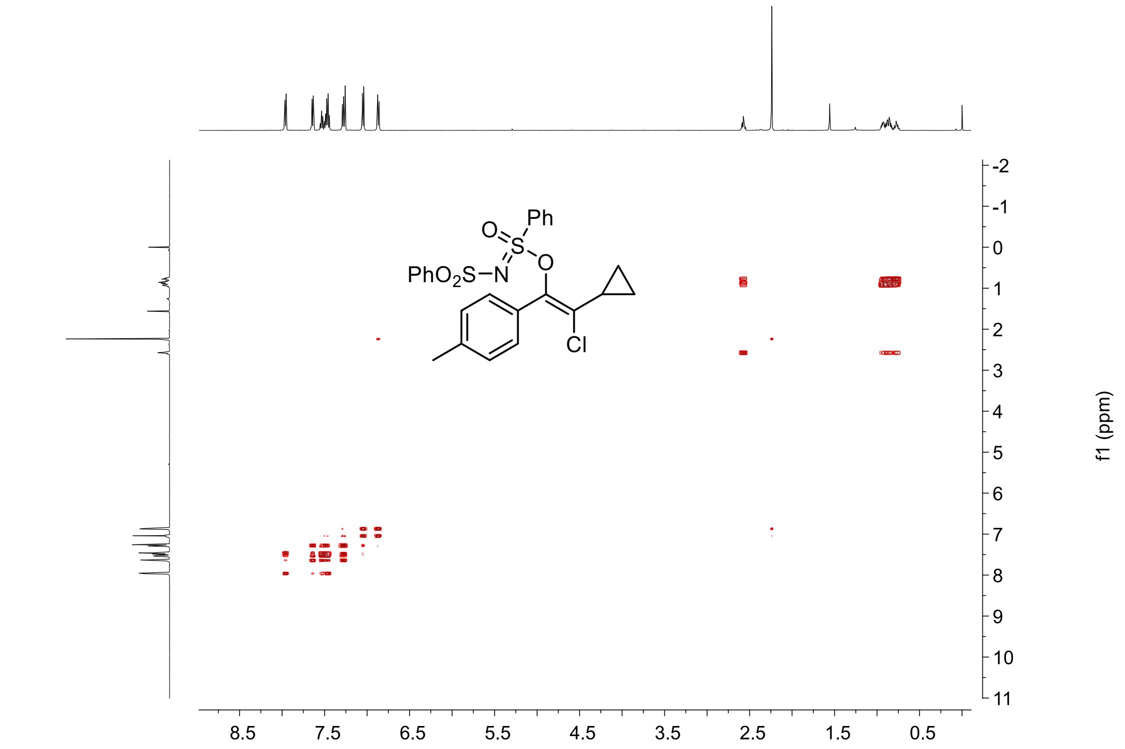

^{1}H-^{1}H Cosy spectrum of 5f.

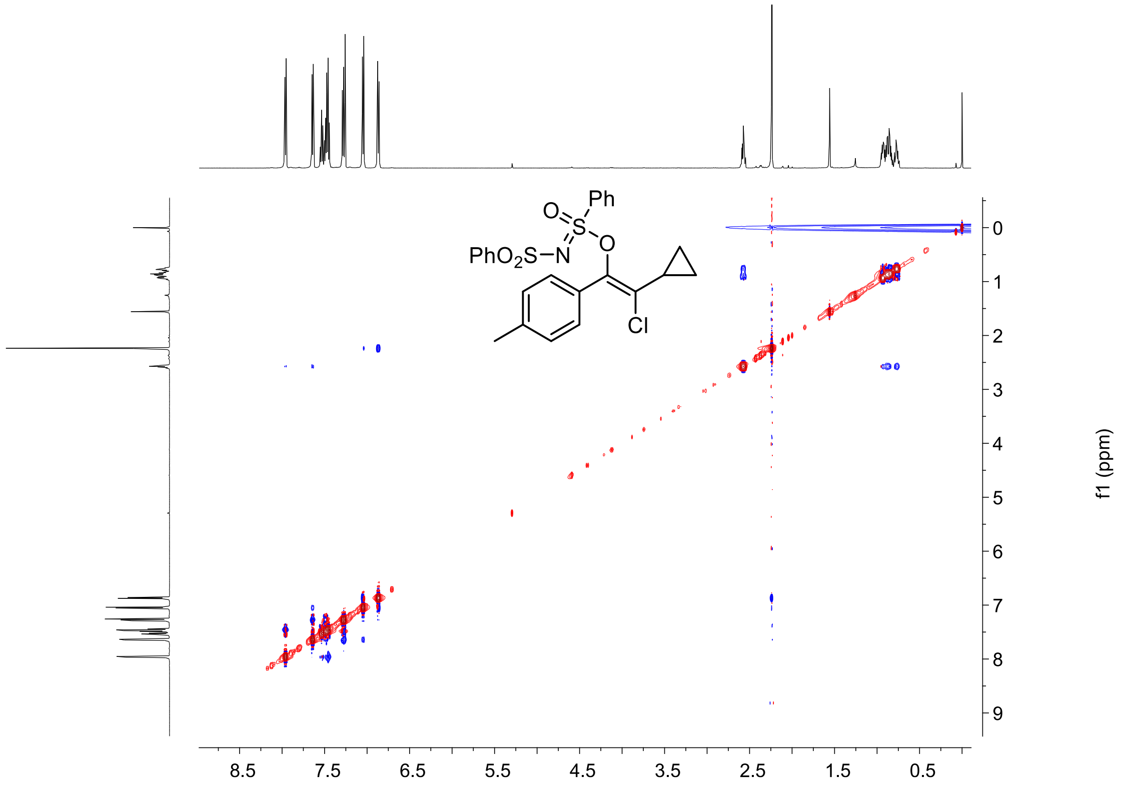

^{1}H-^{1}H NOE spectrum of 5f.

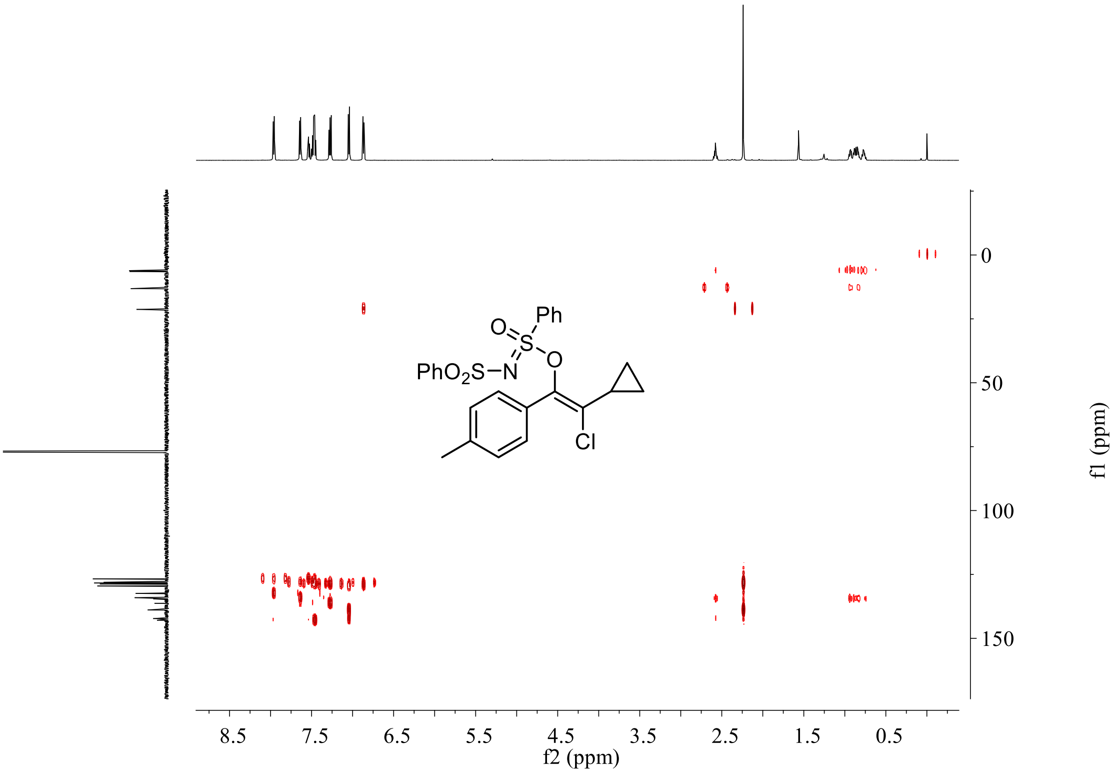

^{1}H-^{13}C HMQC spectrum of 5f.

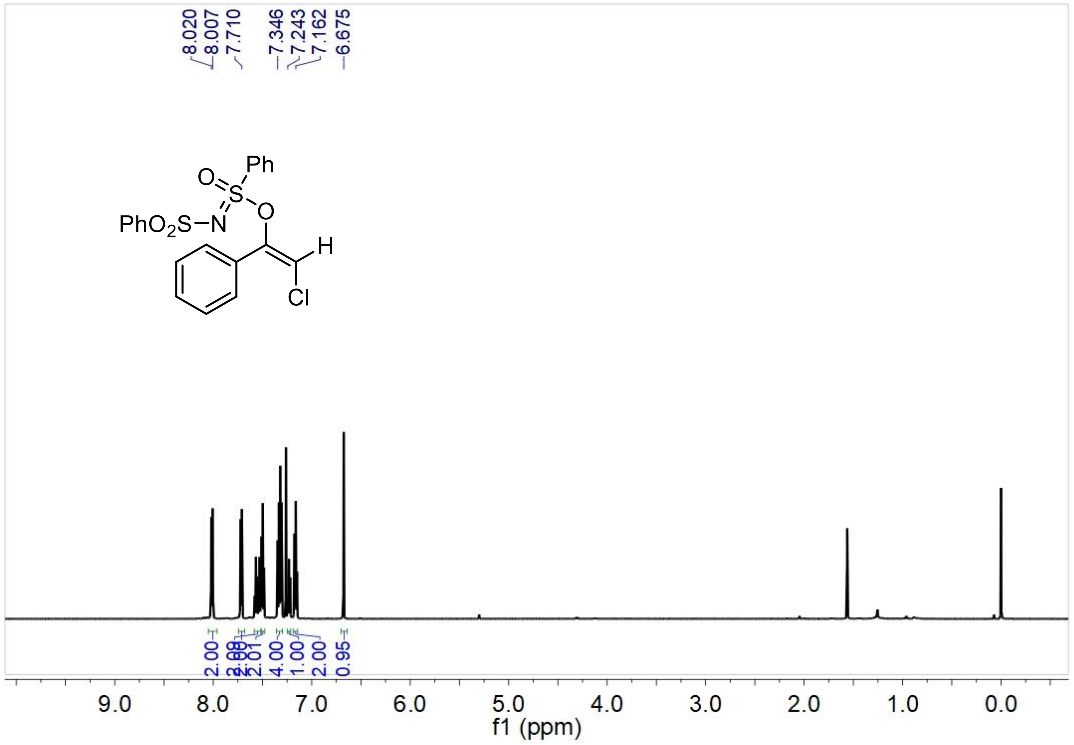

^{1}H NMR (600 MHz, CDCl3) spectrum of 5g.

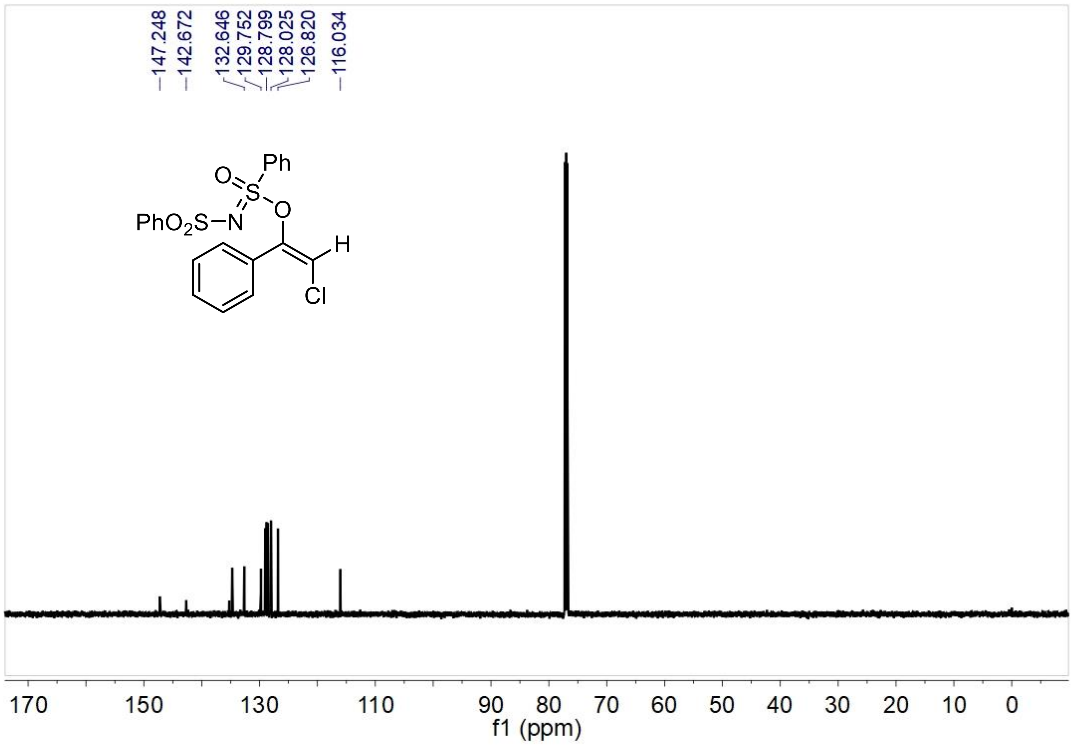

^{13}C NMR (150 MHz, CDCl3) spectrum of 5g.

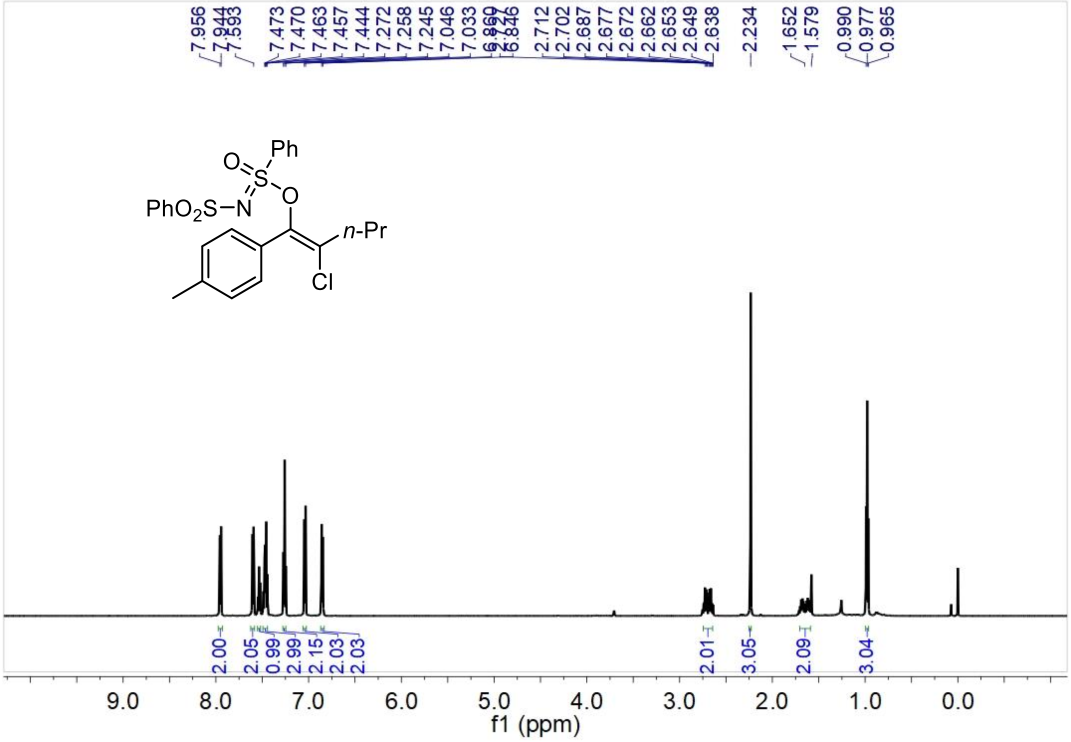

^{1}H NMR (600 MHz, CDCl3) spectrum of 5h.

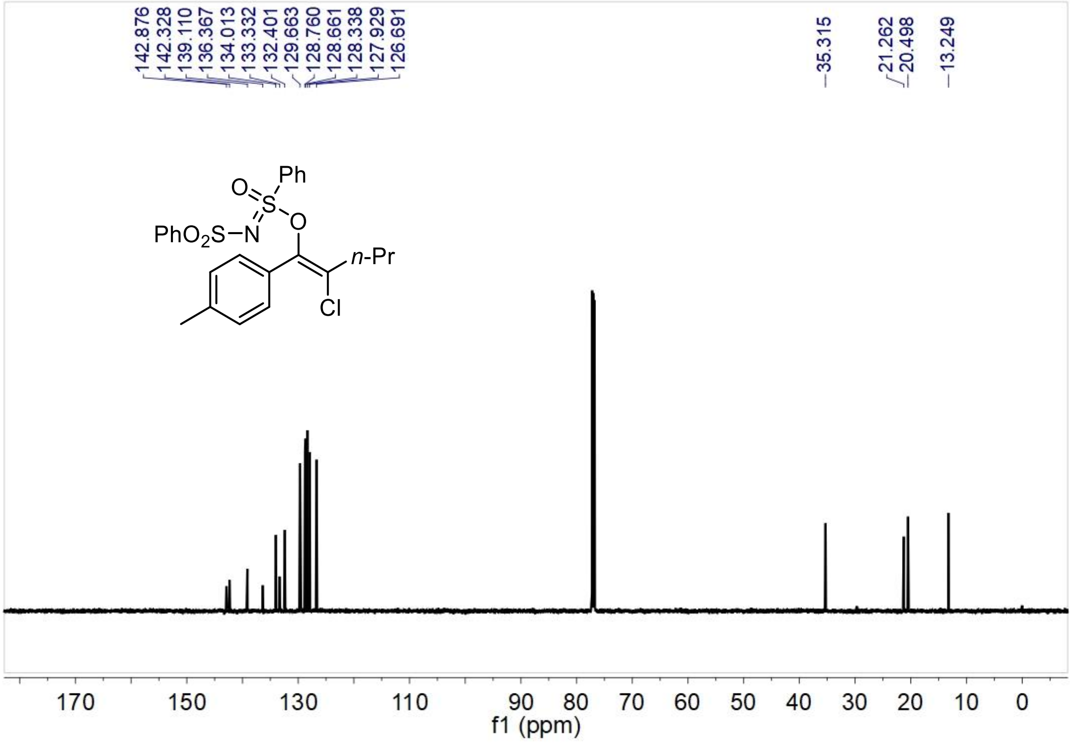

^{13}C NMR (150 MHz, CDCl3) spectrum of 5h.

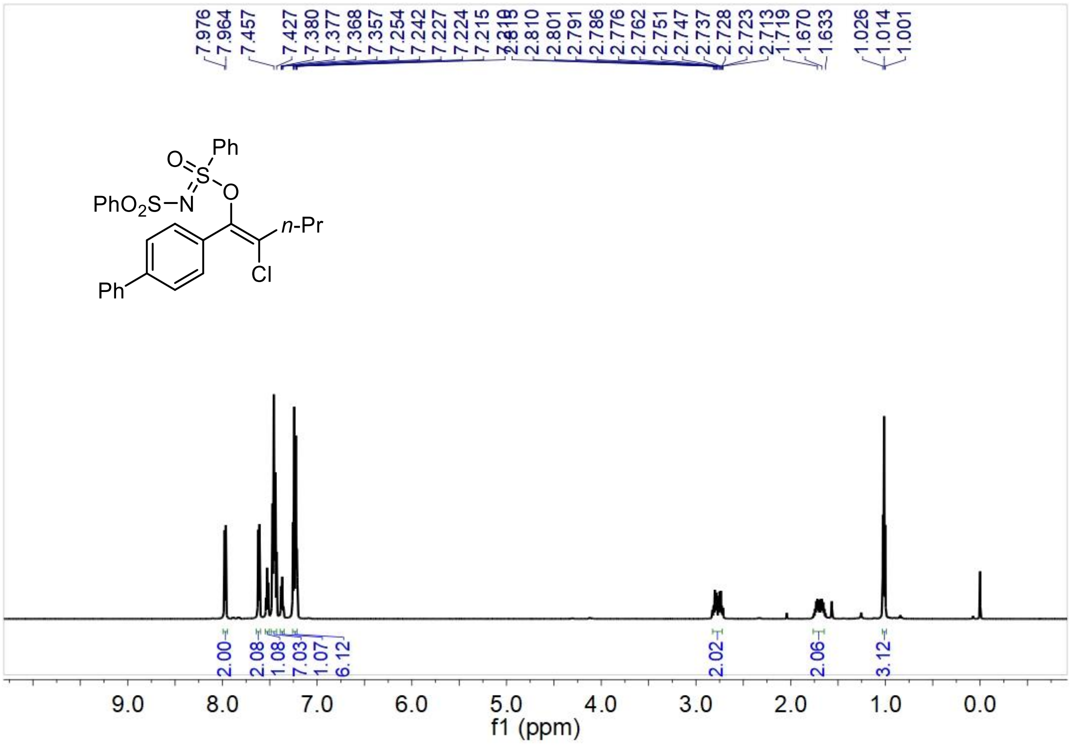

^{1}H NMR (600 MHz, CDCl$_3$) spectrum of 5i.

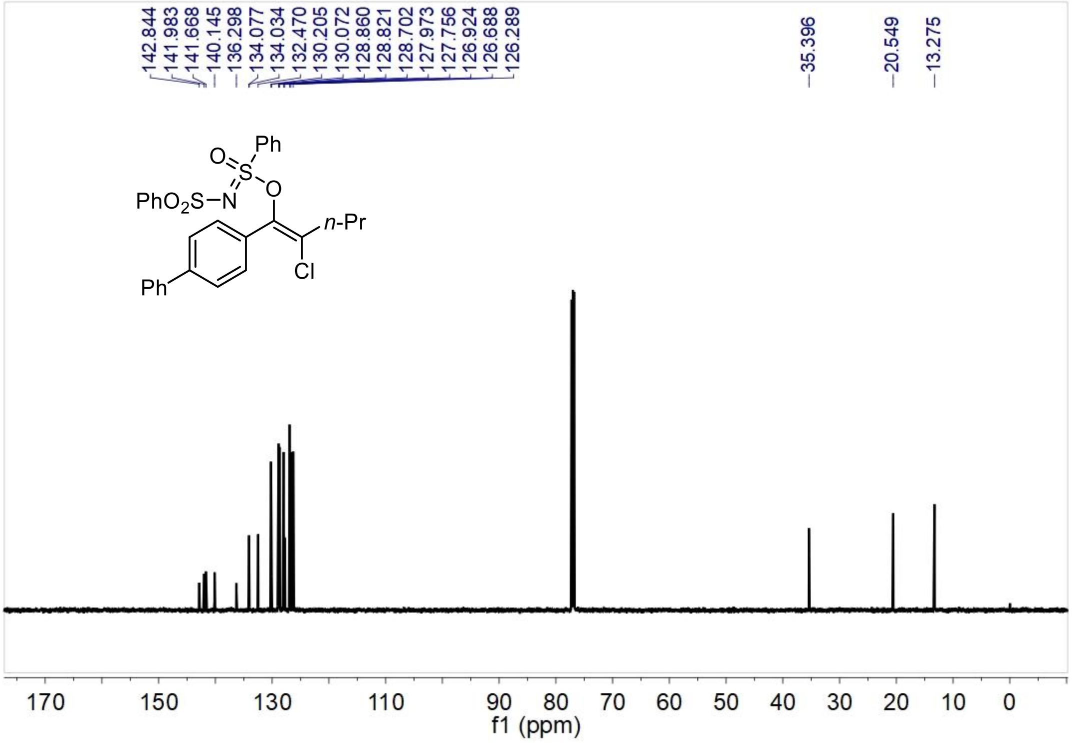

^{13}C NMR (150 MHz, CDCl$_3$) spectrum of 5i.

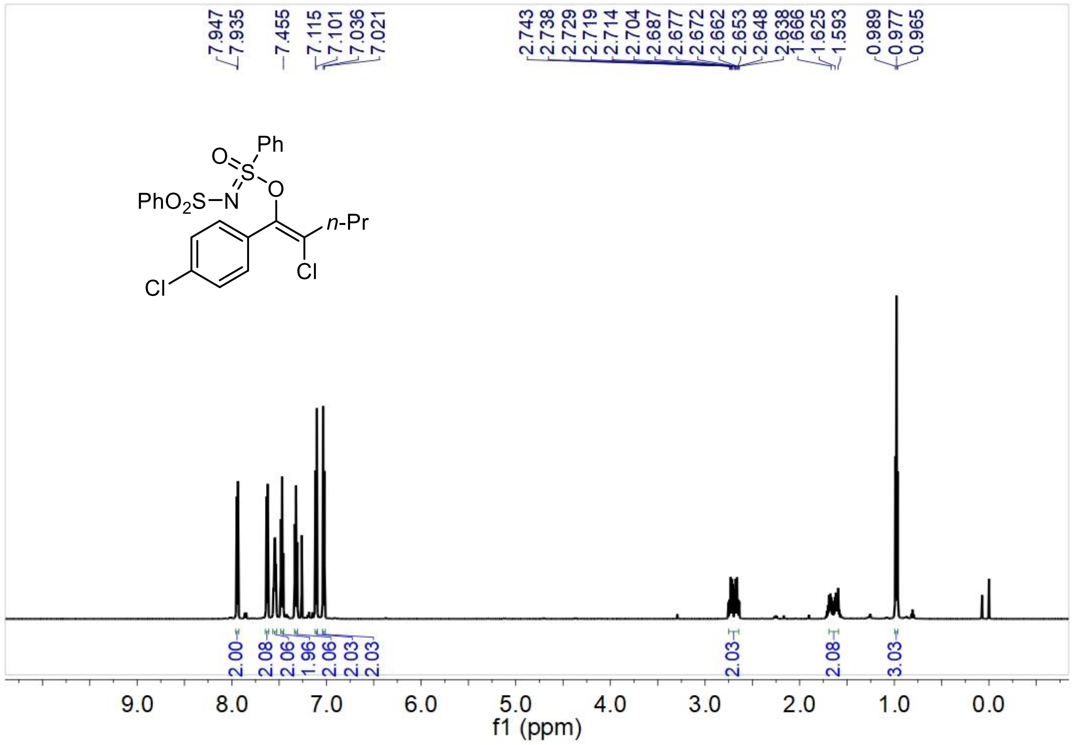

^{1}H NMR (600 MHz, $CDCl_3$) spectrum of 5j.

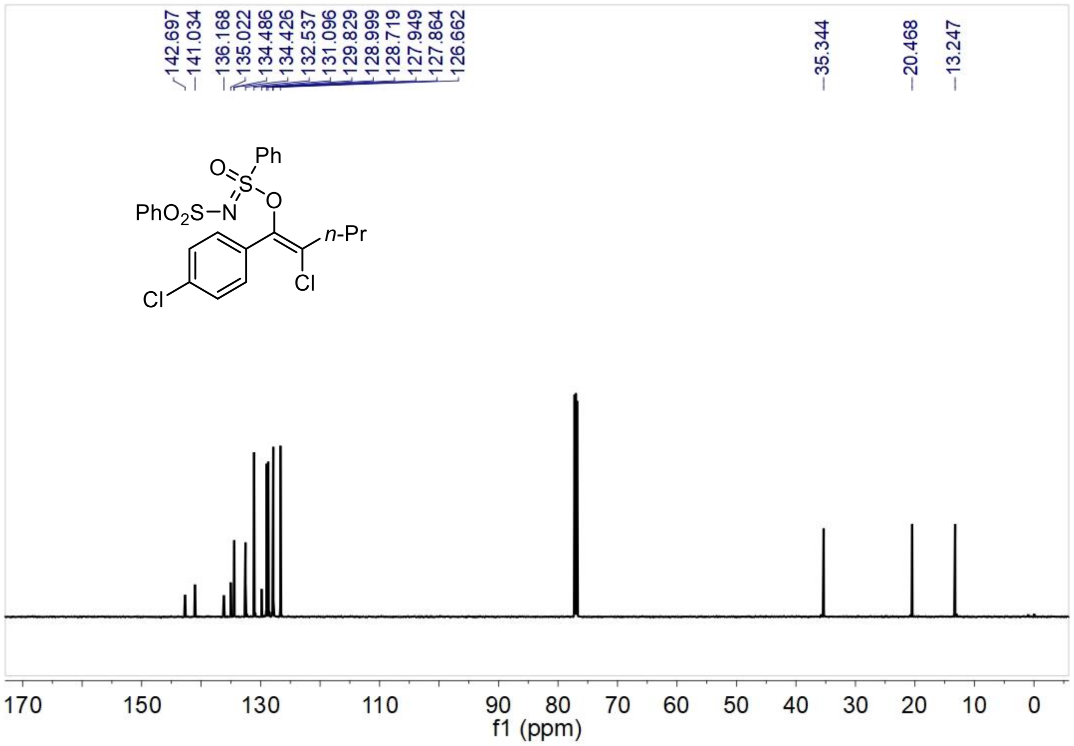

^{13}C NMR (150 MHz, $CDCl_3$) spectrum of 5j.

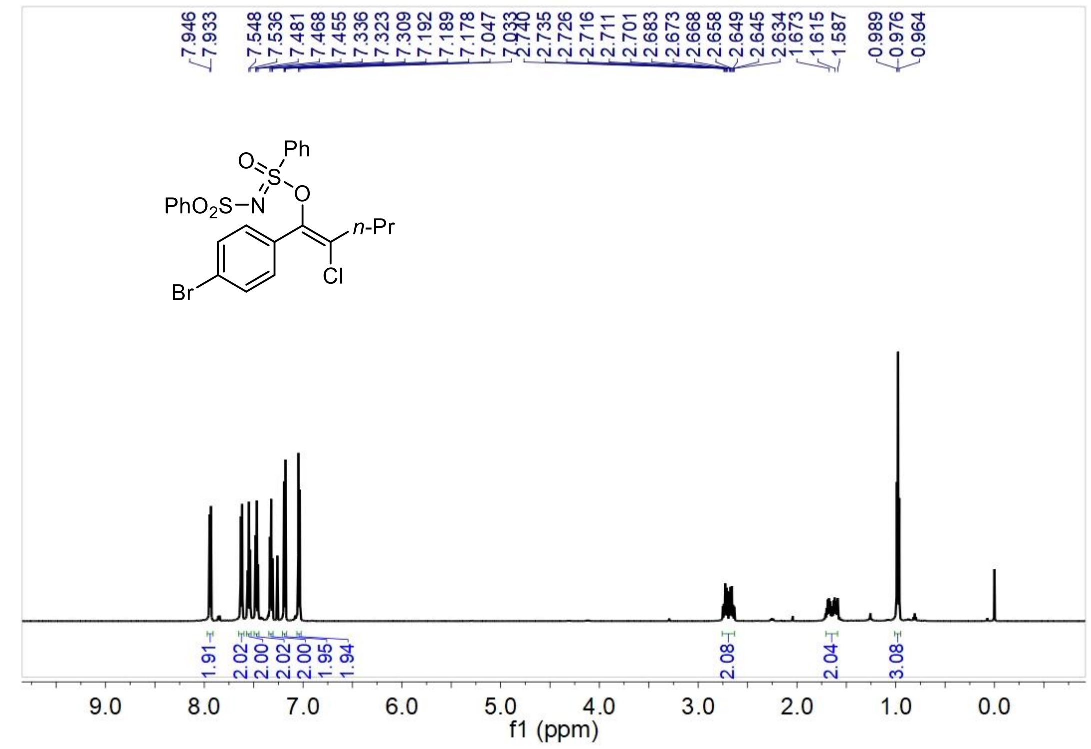

^{1}H NMR (600 MHz, CDCl3) spectrum of 5k.

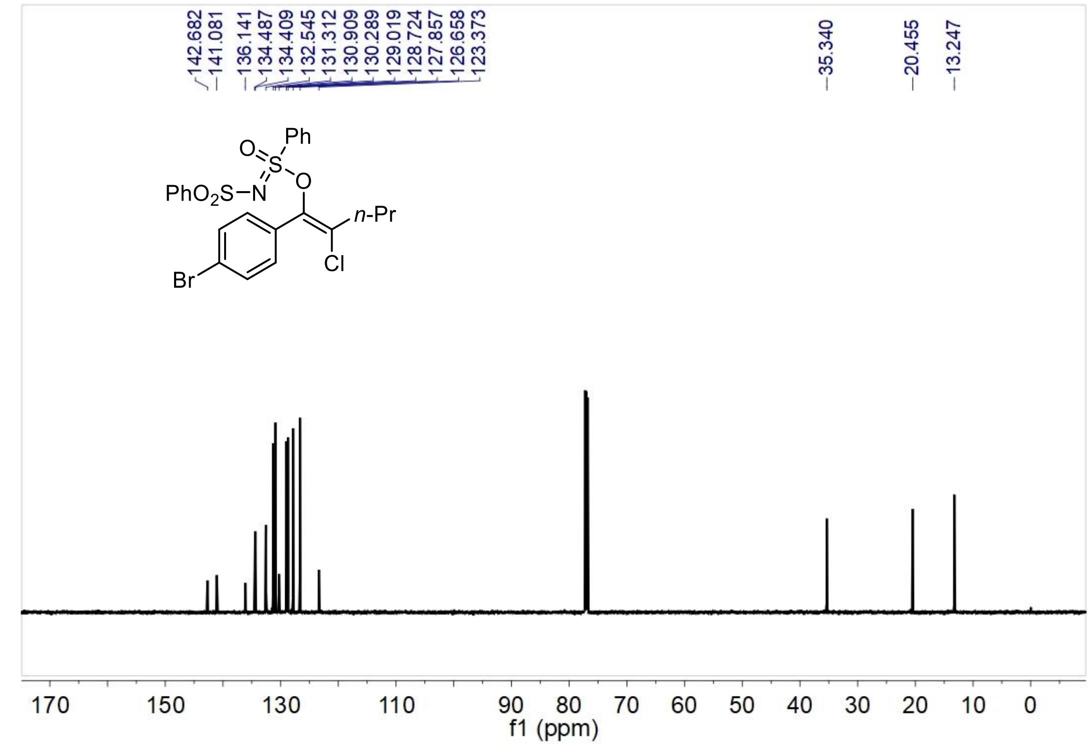

^{13}C NMR (150 MHz, CDCl3) spectrum of 5k.

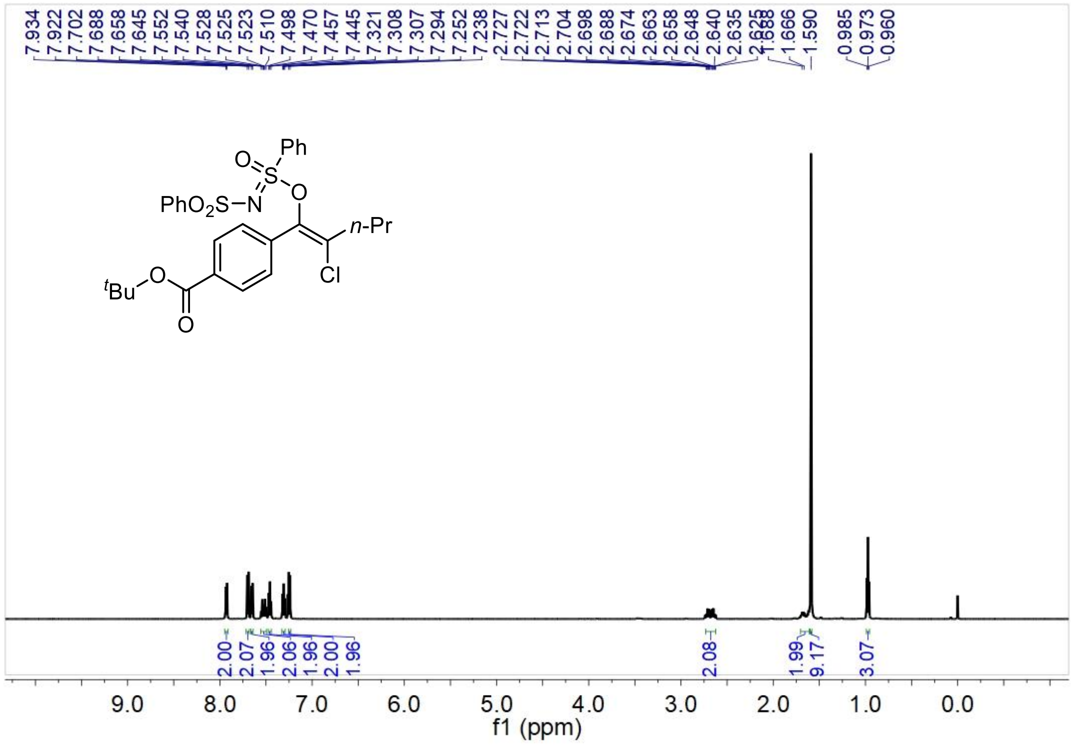

^{1}H NMR (600 MHz, CDCl3) spectrum of 5l.

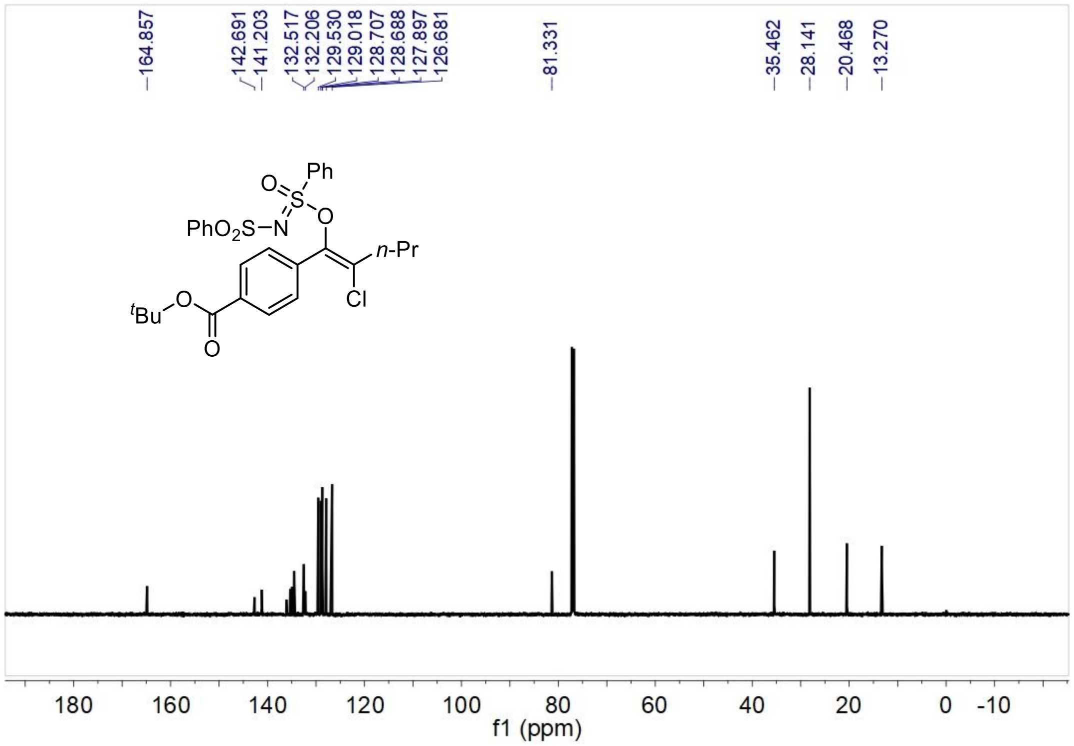

^{13}C NMR (150 MHz, CDCl3) spectrum of 5l.

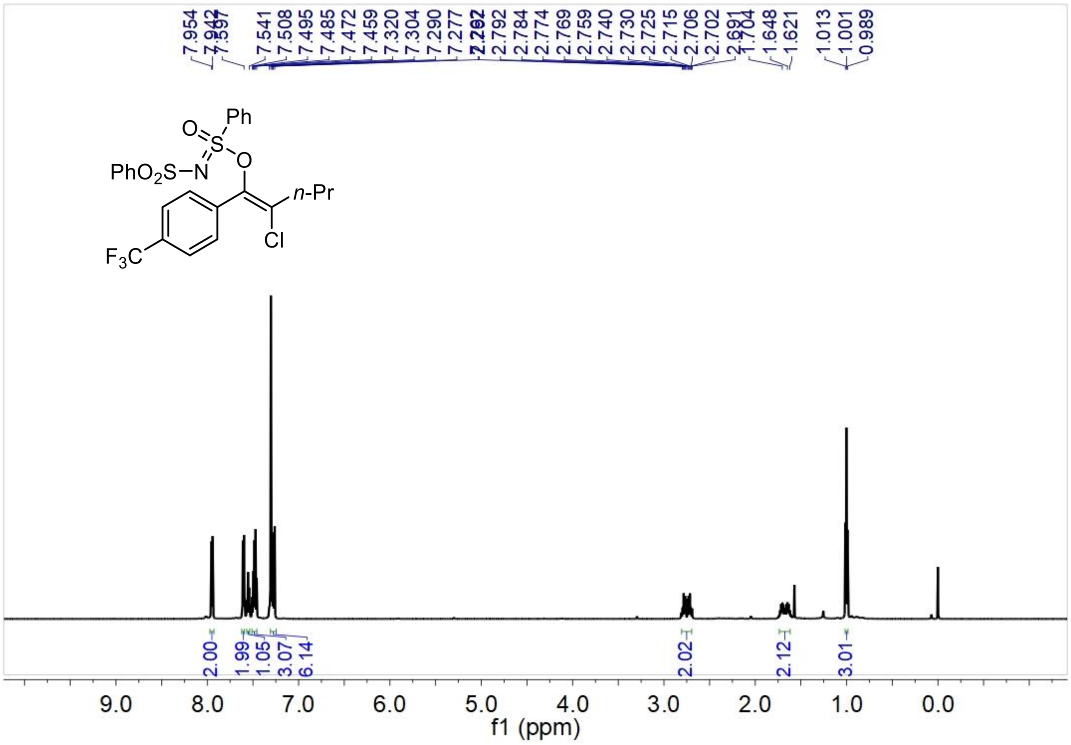

^{1}H NMR (600 MHz, CDCl3) spectrum of 5m.

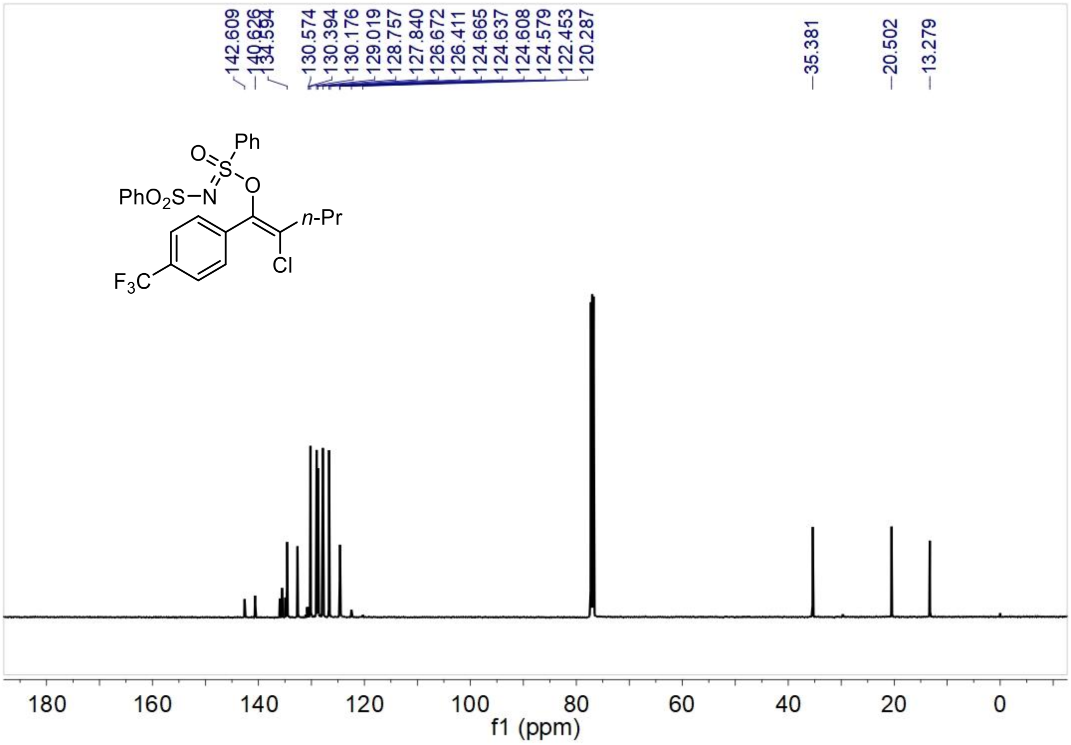

^{13}C NMR (125 MHz, CDCl3) spectrum of 5m.

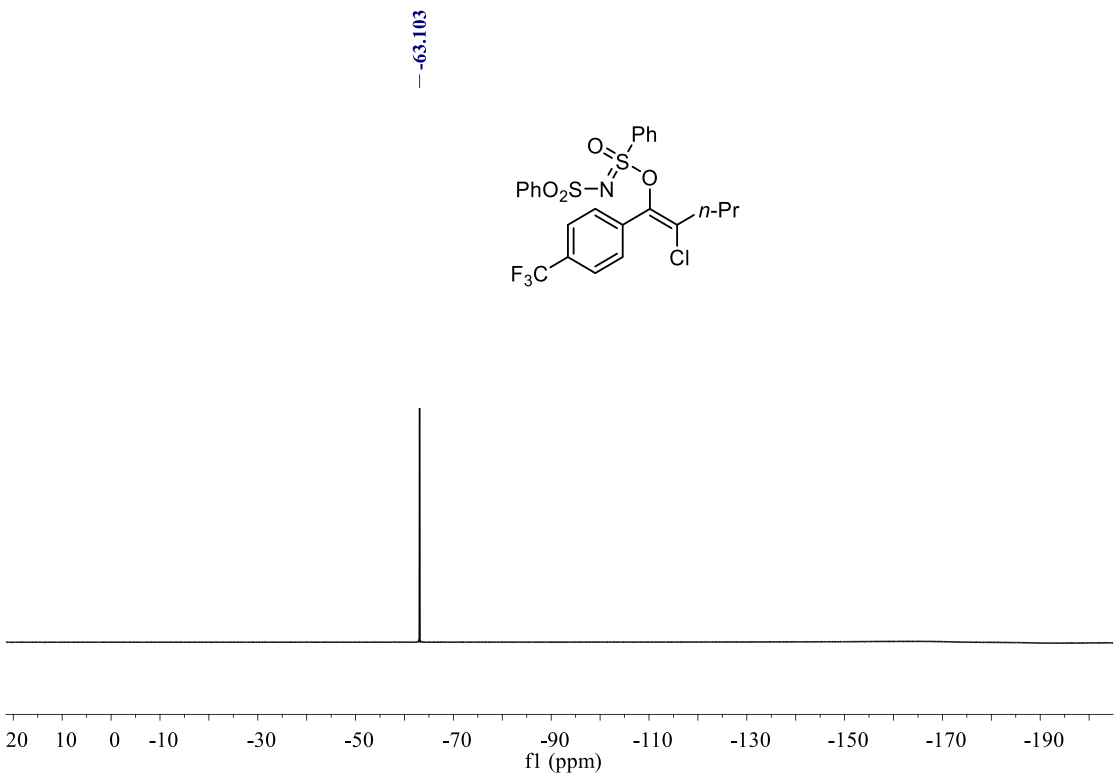

^{19}F NMR (565 MHz, CDCl$_3$) spectrum of 5m.

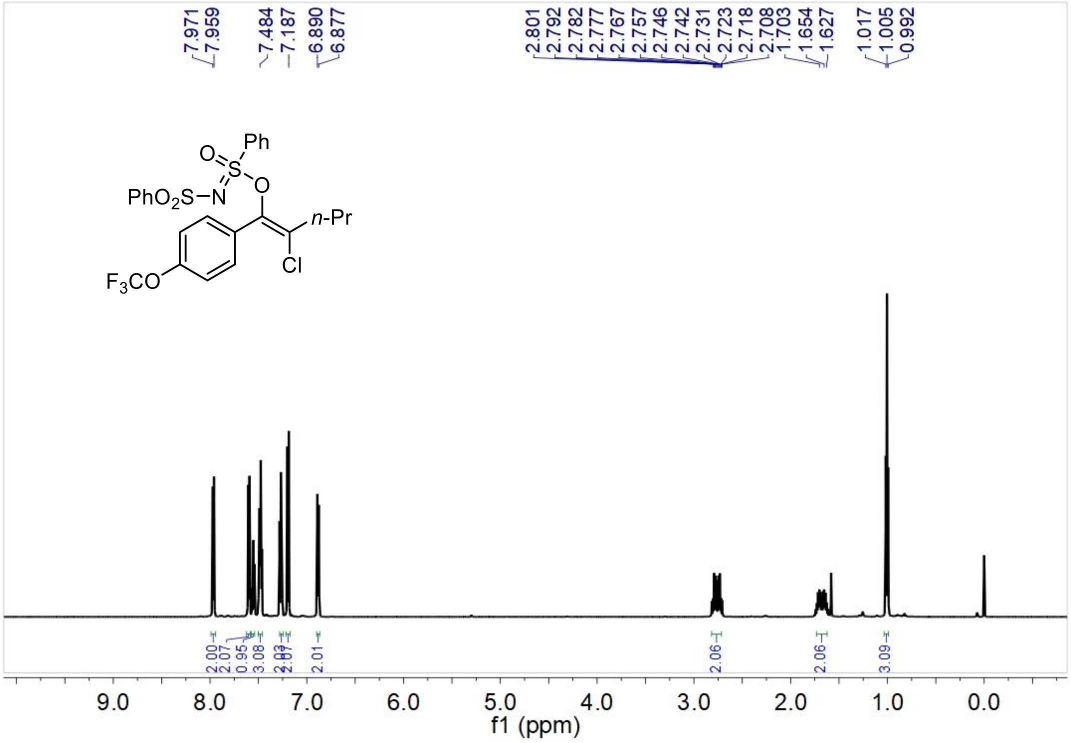

^{1}H NMR (600 MHz, CDCl3) spectrum of 5n.

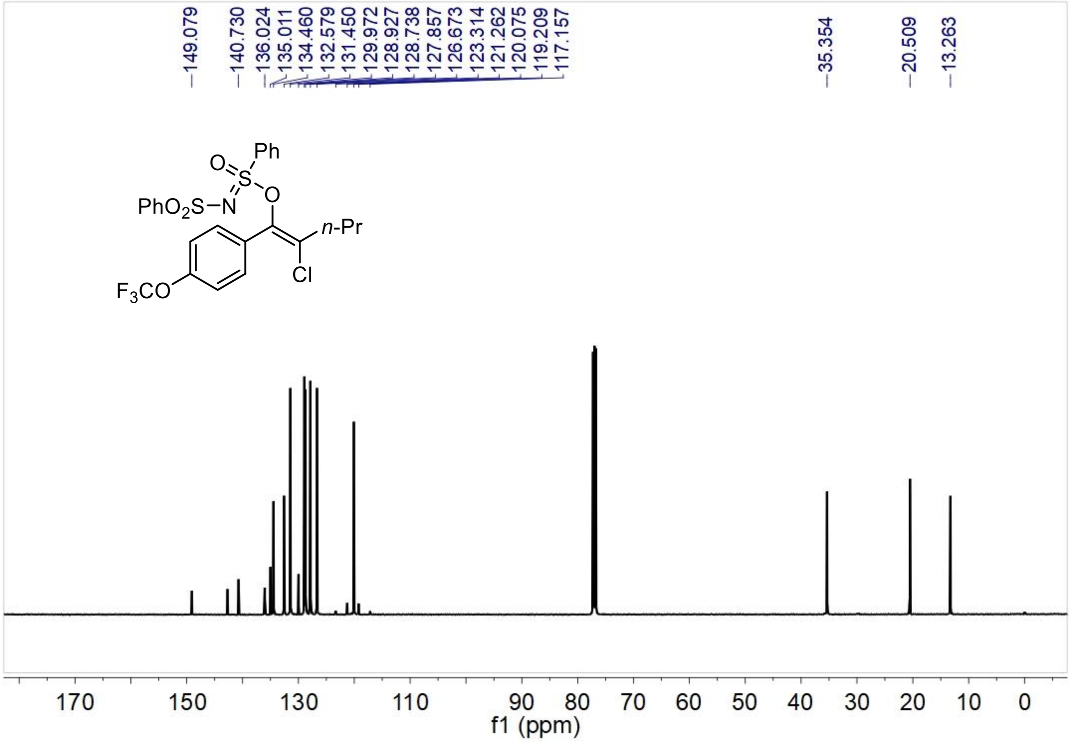

^{13}C NMR (125 MHz, CDCl3) spectrum of 5n.

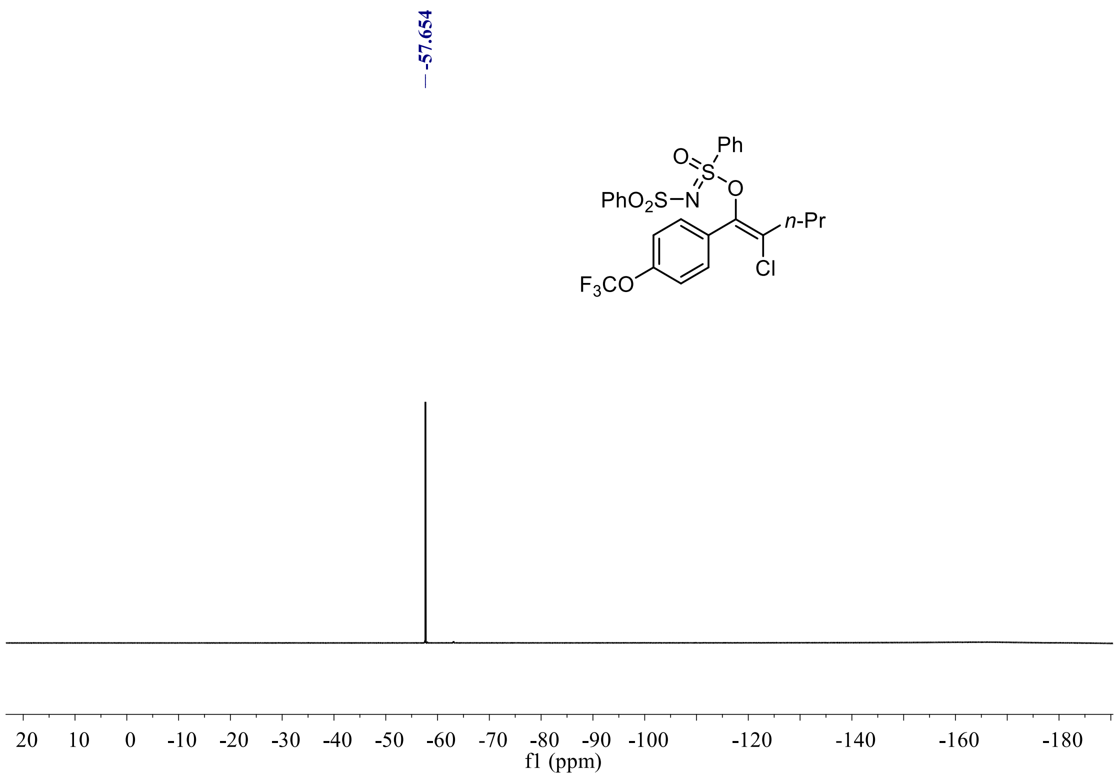

^{19}F NMR (565 MHz, $CDCl_3$) spectrum of 5n.

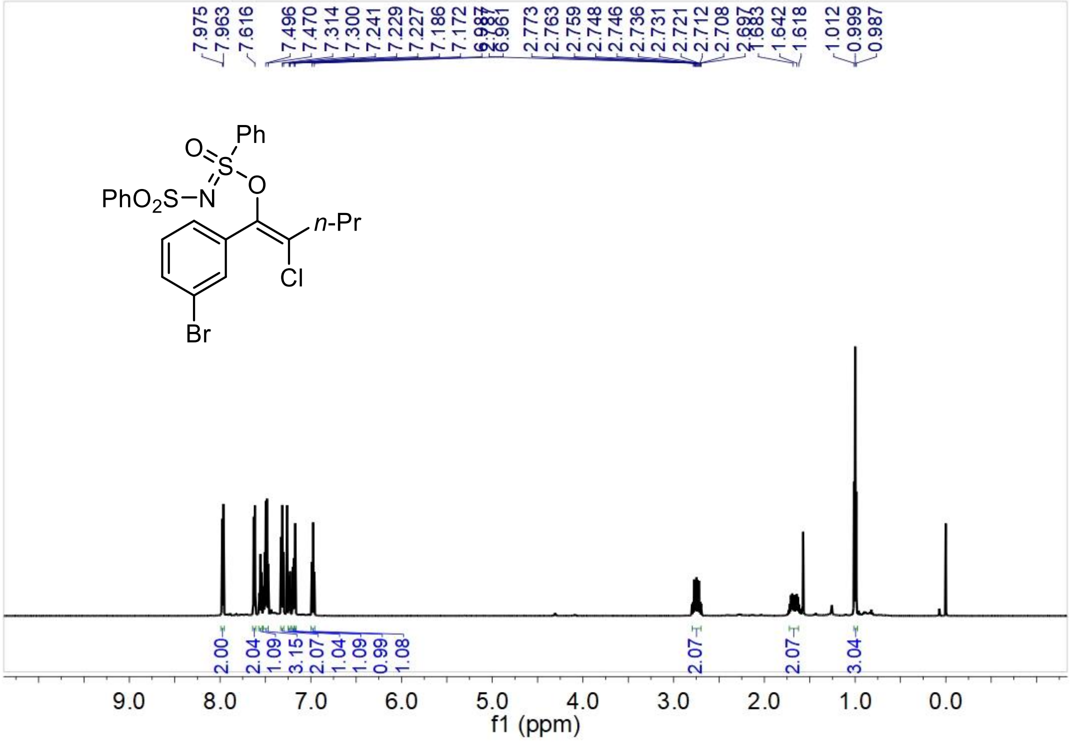

^{1}H NMR (600 MHz, CDCl3) spectrum of 5o.

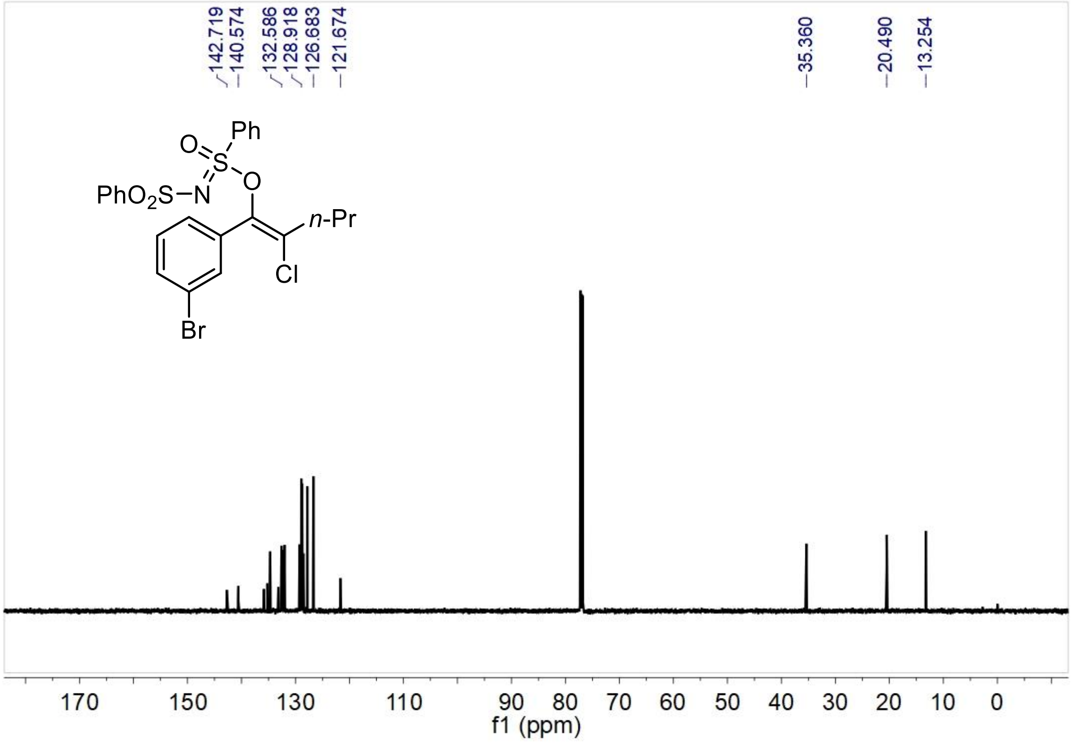

^{13}C NMR (150 MHz, CDCl3) spectrum of 5o.

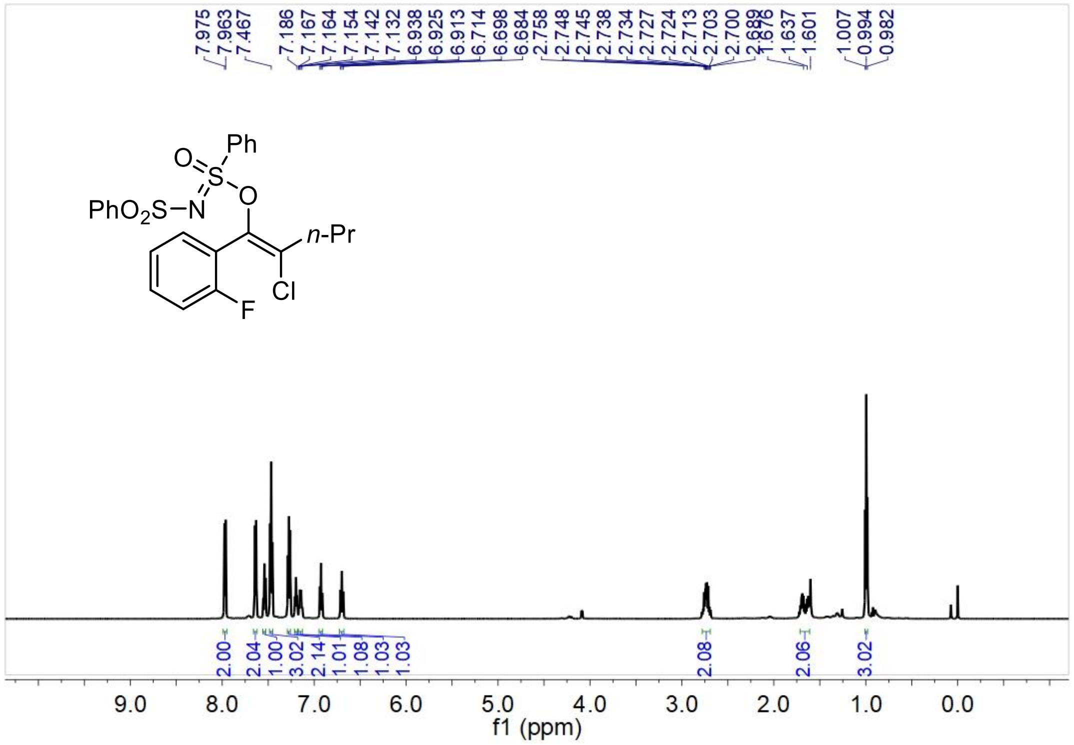

^{1}H NMR (600 MHz, CDCl3) spectrum of 5p.

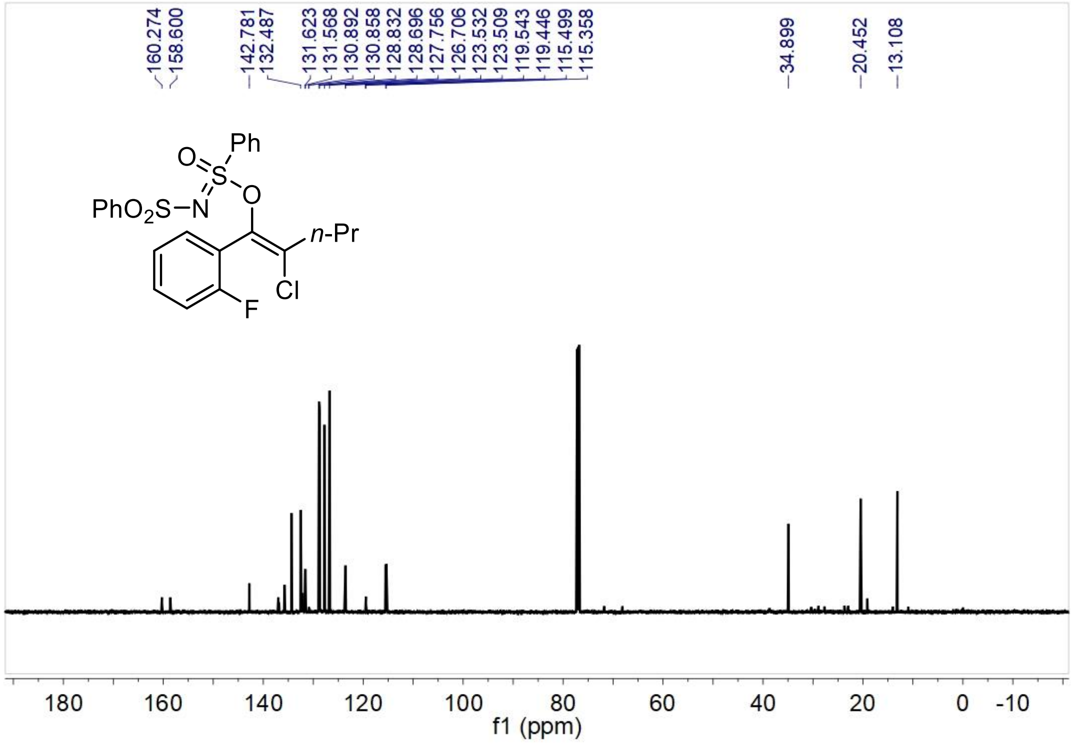

^{13}C NMR (150 MHz, CDCl3) spectrum of 5p.

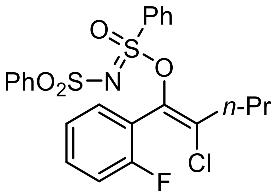

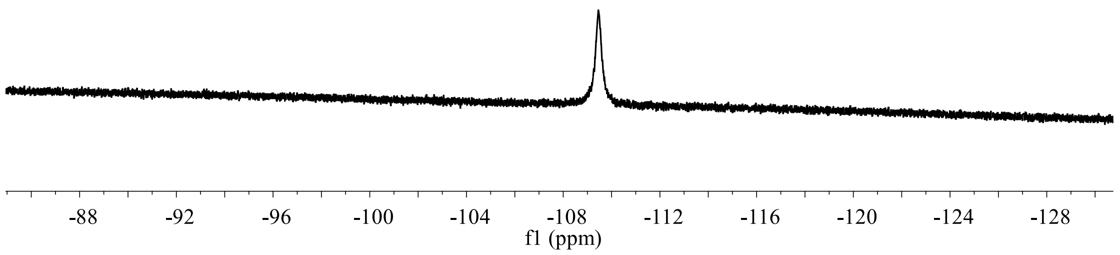

^{19}F NMR (565 MHz, $CDCl_3$) spectrum of 5p.
